# Proposal for a mechanical model of mobile shales

**DOI:** 10.1038/s41598-021-02868-x

**Published:** 2021-12-10

**Authors:** Juan I. Soto, Mahdi Heidari, Michael R. Hudec

**Affiliations:** 1 Bureau of Economic Geology, Jackson School of Geosciences, The University of Texas at Austin, University Station, Box X, Austin, TX 78713-8924 USA; 2 Departamento de Geodinámica, Universidad de Granada, Avenida de Fuente Nueva s/n, 18071 Granada, Spain

**Keywords:** Tectonics, Geophysics, Rheology, Mechanical properties

## Abstract

Structural systems involving mobile shale represent one of the most difficult challenges for geoscientists dedicated to exploring the subsurface structure of continental margins. Mobile-shale structures range from surficial mud volcanoes to deeply buried shale diapirs and shale-cored folds. Where mobile shales occur, seismic imaging is typically poor, drilling is hazardous, and established principles to guide interpretation are few. The central problem leading to these issues is the poor understanding of the mechanical behaviour of mobile shales. Here we propose that mobile shales are at critical state, thus we define mobile shales as “bodies of clay-rich sediment or sedimentary rock undergoing penetrative, (visco-) plastic deformation at the critical state”. We discuss how this proposition can explain key observations associated with mobile shales. The critical-state model can explain the occurrence of both fluidized (no grain contact) shales (e.g., in mud volcanoes) and more viscous shales flowing with grain-to-grain contact (e.g., in shale diapirs), mobilization of cemented and compacted shales, and the role of overpressure in shale mobility. Our model offers new avenues for understanding complex and fascinating mobile-shale structures.

## Introduction

**Figure 1:**
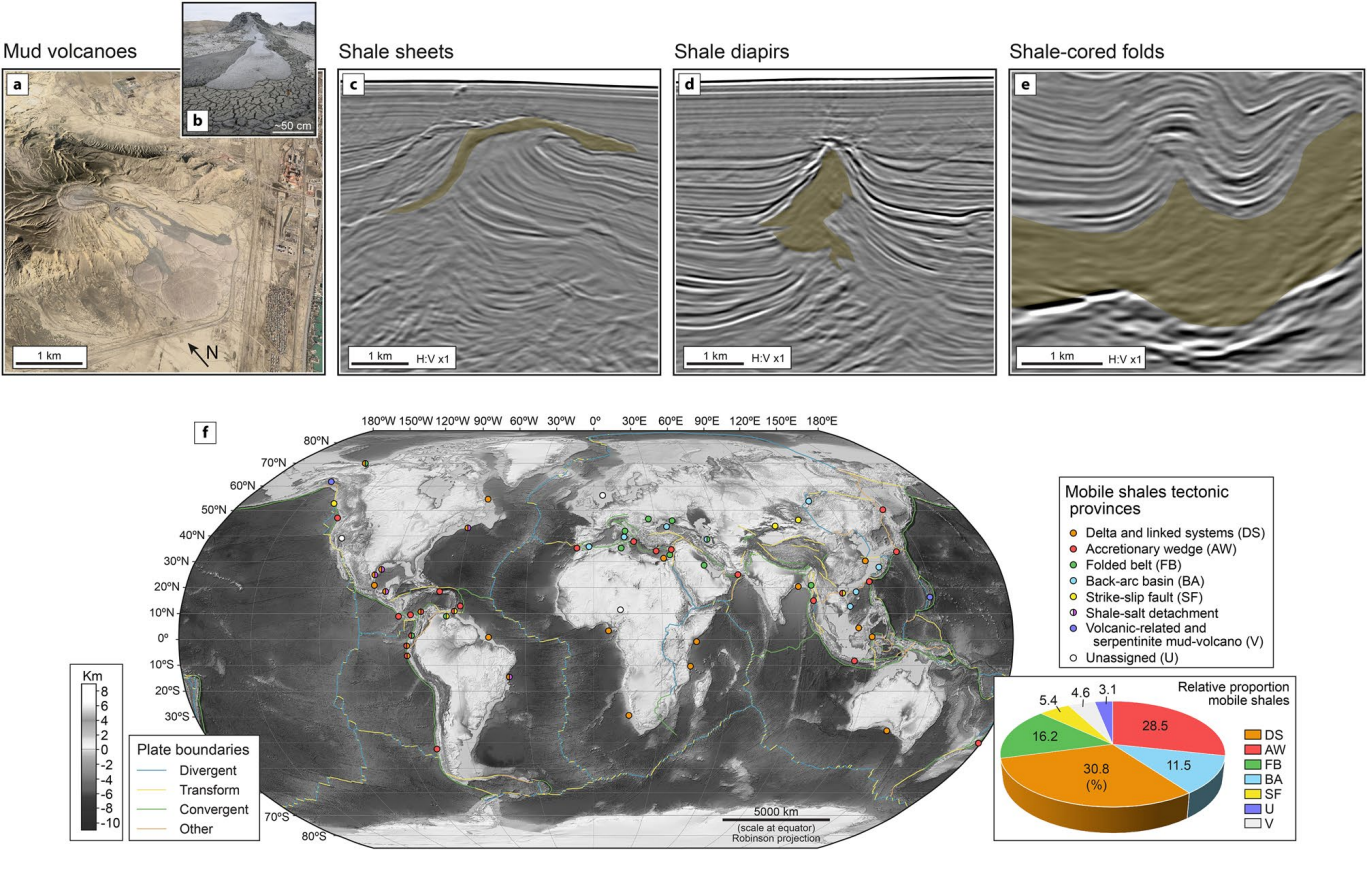
Structures formed by mobile shales. (**a**) Garadagh mud volcano in onshore Azerbaijan (40.24°N and 49.51°E). (**b**) Gryphon structure within the Dashgil mud volcano^1^ in onshore Azerbaijan (39.99°N and 49.47°E). (**c**–**e**) Variety of buried mobile shales, as seen in seismic profiles (offshore, north-western Gulf of Mexico): (**c**) shale sheet; (**d**) shale diapir, and (**e**) shale-cored folds. Seismic images extracted from a 3D depth seismic cube (seismic data courtesy of PGS). (**f**) Global distribution of mobile-shale structures over plate-tectonic map of Earth. Surface-elevation model and complete details of compilation in Supplementary Information 1 (Fig. s1 and Table s1). Map plotted using Robinson projection (using ArcGIS 10.8; https://www.esri.com/en-us/arcgis/products/arcgis-desktop/overview) and drafted using Adobe Illustrator 2021 (https://www.adobe.com/products/illustrator.html). Inset pie diagram shows relative proportion (in %) of mobile shales according to their tectonic setting (for total population of 65 regions; Supplementary Table s1).

Mobile shales are bodies of highly sheared shale that lack coherent reflections in seismic images. Mobile-shale structures range from surficial mud volcanoes to deeply buried shale diapirs and shale-cored folds (Fig. 1a–e). Although they exist in all tectonic regimes (Fig. 1f), they are mostly found in shortening settings (40%) or delta systems on continental margins (31%) (detailed information in Supplementary Information 1).

Mobile shales create challenges for seismic processing, seismic interpretation, drilling, and geohazards analysis. A primary source of uncertainty in interpreting these structures is a poor understanding of the mechanics of mobile shale. Although mobile shales appear to deform through some sort of ductile flow that strongly modifies previous structures like fractures, dissolution and precipitation micro-structures, and even the shape and dimensions of the voids^2–7^, we have little insight into how shales become mobile and flow. Most authors suggest that shales become mobile simply by becoming highly overpressured^8–11^, but this simple explanation fails to account for the full range of mobile-shale occurrence. For example, shales (defined broadly, sensu Aplin et al.^12^) can apparently become mobile even after significant burial, consolidation, and cementation^13, 14^. There are also examples of organic-rich shales that can form complex flow structures under contraction, even though they have low porosity^15^. Overpressure can mobilize unconsolidated mud, but an increase in pore pressure alone cannot cause a cemented shale to flow.

Our lack of a viable mechanical model for mobile shale is due partly to the difficulty in sampling subsurface mobile shales and measuring their mechanical properties. Some information can be extrapolated from wells penetrating clay-rich shear faults in seismogenic zones and oceanic-wedge detachments^16, 17^. Mud volcanoes have been extensively studied^18–22^, and progress has been made in studying plastic flow of the extruded sediments using sampling observations^23–27^ and some drilling data^28–31^. Nevertheless, we have little knowledge of the physical properties of plastically flowing shales in the subsurface and even less knowledge of the mechanical processes by which they become mobile or stop moving.

This uncertain understanding is reflected in the existing definition of *mobile shale*. The term was introduced by Morley and Guerin^4^, who defined them as “any shales deforming complexly by a combination of ductile deformation and brittle failure in the presence of a fluid phase.” This definition was an excellent starting point because it recognized the roles of lithology, deformation style, and fluids in shale mobility. However, it suffers from several shortcomings. First, the definition is overly broad because it includes almost any type of deformation involving shales. Second, it provides little insight into what makes a mobile shale mobile or how it deforms.

In this paper, we summarize observations concerning mobile shales and, using concepts coming from soil mechanics and available information on the mechanics of consolidated shales, we then propose a mechanical model for mobile shales. We explore the implications of this model and its answers to key questions in mobile-shale research: How do shales become mobile? Is shale mobilization a brittle or ductile process? Why do some mobile shales become immobile? Can shales with diagenetic cements become mobile? Does depth of burial play a role in shale mobilization? Do all mobile shales have to be highly overpressured? If so, how high must the overpressure be? How does shale mobilization affect its seismic properties?

## What do we know about the mechanics of mobile shales?

There are some observations about mobile shales that are widely accepted. We use these observations as a framework to suggest a mechanical model for mobile shales.

**Figure 2:**
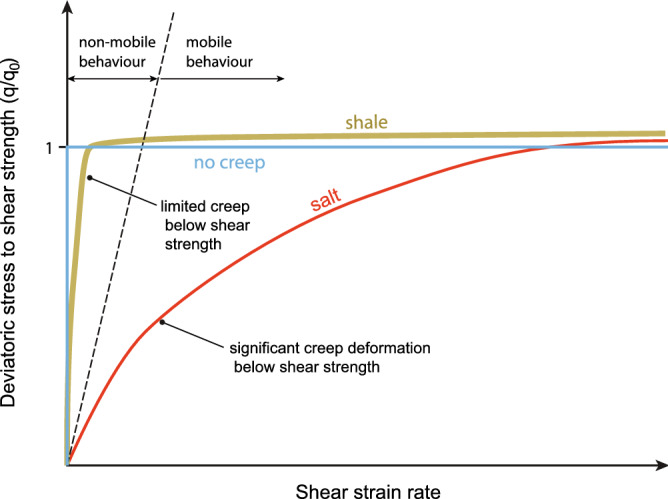
Viscous behaviour of salt versus shales. Plot showing schematically the viscous behaviour of salt (red curve) and ductily-deformed shales (brown curve) in a diagram with shear strain rate against the ratio between the shear stress (q = σ_1_ − σ_3_) and the shear strength at critical state (q_0_) (Fig. 3). In this case, it is assumed that the shale sample experiences a limited amount of creep before peak strength. This part of the deformation is contained in the sub-vertical portion of the shale path. Once the sample surpasses the peak (or yield) strength and ductile deformation occurs, the path approaches the horizontal unity line. The intersection between the shale path and this line marks critical state, and any further deformation occurs without any significant increase in the deviatoric stresses and progress under critical-state conditions; i.e., the path follows a sub-horizontal trend above the unity line. By comparison, salt mostly deforms by creep under deviatoric stresses lower than the shear strength at critical state, and is even mobile from very low strain rates and deviatoric stresses. It is also included for comparison, the path of a rock without creep deformation (blue line). Mobility can also be achieved by this type of material under larger shear strain rates. The approximate limit between non-mobile and mobile behaviour is marked by a discontinuous line, which would have a variable slope depending on the viscosity of the material.

First, the main difference between salt and sedimentary rocks like shales is their contrasting mechanical behaviour (Fig. 2). Salt can experience large-scale deformation by creep at stresses below its yield strength^32^. By contrast, only limited subcritical (pre-peak strength) creep deformation is documented to occur in shales, although creep seems to be more important in clay- and organic- rich shales^33^. Creep in shales is promoted by particle dissolution, grain rotation and sliding, pore reduction, and the expulsion of fluids from pores and the clay-bound water^34, 35^. Most of the deformation in shales occurs after stresses surpass peak strength.

Secondly, there are two distinct types of mobile-shale structures^7^. The first occurs when the shale behaves as a fluid suspension without grain-to-grain contact^36–40^. Because of their low viscosity (ca. 10^1^–10^6^ Pa s^26, 27^), this type of mobile shales forms smaller features like mud volcanoes (Fig. 1a,b) in which the shale moves at high velocities (up to tens of meters per second)^20–22, 41^. In the second type, shale behaves as a viscous-plastic solid involving brittle and ductile fracturing, and grain-to-grain frictional flow^5, 6, 8, 15, 42^. Because of their high viscosity (ca. ≥ 10^15^ Pa s^43^), this type of mobile shale forms large-scale bodies like shale diapirs (Fig. 1c–e) that move at lower velocities than in mud volcanoes.

Third, bedding and other fabrics in mobile-shale structures are strongly disrupted, recording large and extensive deformation, and possibly fracturing. This disruption is one factor causing the loss of seismic signal in mobile shale (Fig. 1c–e)^11, 14, 15, 30, 44–46^.

Fourth, mobile shales are typically associated with high overpressures; i.e., there is a large difference between the pore pressure in the rock and the hydrostatic pressure gradient^28, 29, 31^. Overpressure is obvious in the fluidized material erupted from mud volcanoes, where there is almost no contact between grains^18–20, 22, 23^. High overpressure in other types of mobile shales such as shale diapirs can be inferred from the low seismic velocities of these bodies^4, 7, 14, 30, 45^. Most researchers attribute these overpressures to a combination of increase in the volume of the pore fluid (due for example to hydrocarbon generation and cracking, diagenetic transformations, or thermal expansion) and in total compressive stresses^47, 48^. For example, based on the abundance of methane and other gases (e.g., CO_2_, N_2_, other alkanes, He, Rn) expelled during mud-volcano eruptions^18–23, 41, 49, 50^, the mobility of shale in mud volcanoes is also attributed to hydrocarbon transformations and the depressurization of a gas-charged source layer.

Fifth, given the stratigraphic position of blocks ejected from mud volcanoes, some mobile shales are sourced from depths of 9 to 10 km^20, 22, 41, 51, 52^ (detailed information in Supplementary Information 1). Units sourced from these depths were cemented prior to incorporation in mud volcanoes. This observation represents a challenge for workers in mobile shales, because it is difficult to explain how these cemented blocks were transported.

Any viable mechanical model for mobile shales must be able to explain and be consistent with these observations: fluidization and plastic flow of shales, disruption of fabrics, existence of high overpressure, and mobilization of cemented units.

## Mechanical model for mobile shales: deformation at the critical state

Field observations of subsurface mobile shales are scarce owing to the understandable reluctance of drillers to penetrate them^29^. The model that we propose for mobile shales is based on experimental deformation of shale shear behaviour. Because these tests are typically conducted at low stresses and on poorly lithified soils, they do not directly mimic subsurface conditions. The principles governing the mechanical behaviour of soils^53, 54^ have also been used to analysed the behaviour of consolidated shales, although important differences exist between the mechanical behaviour of soils and shales^55–57^. According to these studies, shales differ from soils in that they have a major cohesion (stiffness), develop some degree of cementation and anisotropy, and their mechanical characteristics change with depth and temperature (detailed information in Supplementary Information 2, Table s2 and Figs. s2–s5). Principles of soil mechanics have also begun to be extrapolated to depths at which diagenetic transformations operate in shales so that the mechanical behaviour of cemented shales deformed under contraction might be modelled^8, 24, 25, 58–61^.

We use the behaviour observed in laboratory tests to infer the mechanical behaviour of mobile shales. In our description, the effective stress (σʹ) is the total stress (σ) less some portion of the pore pressure (u), following the Terzaghi’s equation:

$$\sigma^{\prime } = \sigma - \alpha \cdot u.$$

The Biot’s pore pressure coefficient (α) is usually assumed to equal 1 in soft and unconsolidated muds, although in low porosity shales, α lies between 0.3 and 0.9, decreasing with increasing stress^48, 62^.

**Figure 3:**
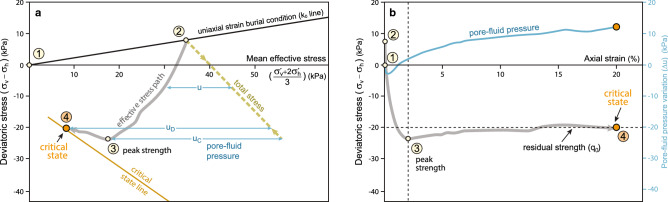
Mechanical behaviour of shales. (**a**) Stress paths of an anisotropic clay (Norrköping clay) as depicted in the p′–q space (mean effective stress vs. deviatoric stress), according to experimental results of undrained triaxial compression tests^63^ (using results with high pre-consolidation stress). (**b**) Stress–strain curve of sample (axial strain vs. deviatoric stress) and variation of pore-fluid excess pressure during experiment. Clay sample with an effective friction angle (ϕ) of 30° and an effective cohesion of 14.2 kPa^63^. Additional experiments documenting how shales can achieve critical state conditions in Supplementary Information 2 (Fig. s2). Stages (encircled numbers): 1: sedimentation; 1 to 2: uniaxial strain consolidation; 2 to 3: undrained shearing deformation, sample exhibiting strain-hardening until peak strength (3); and 3 to 4: undrained-shearing, sample exhibiting strain-softening due to fabric collapse at critical state (4).

Figure 3 illustrates the total and effective stress paths and the stress–strain response obtained from an undrained triaxial test on Norrköping clays^63^ (Fig. 3). In these tests, the clay sample, retrieved from a core, was first consolidated under uniaxial-strain condition to the in situ vertical effective stress (point 2, Fig. 3a). This represents the loading on the clay as it was buried to its final depth. Then, the sample was compressed horizontally in undrained conditions; during this period, the total vertical stress (σ_v_ʹ) was kept constant (path from 2 to 4, Fig. 3a). This represents the loading that the clay would undergo if it was in a region with horizontal shortening. The stress path in this test represents the dominant stress path in fold cores, which is a common setting for mobile shales (Fig. 1f). The test was conducted under undrained conditions because shales have very low permeability, therefore, pore fluid could barely drain out of buried shales during shortening.

The clay response during undrained shortening includes two distinct phases (path from 2 to 4, Fig. 3b). First is a period of strain hardening (path 2 to 3), during which deviatoric stress (q, ordinate axis in Fig. 3a) increases as the clay is compressed horizontally and the shear deformation increases. During this period, the effective mean stress (p′, abscissa axis in Fig. 3a) decreases. The sample tends to compact as it is sheared, but this compaction is prevented by pore fluid that cannot escape, leading to an increase in pore pressure (shear-induced overpressure^48, 62^) and decrease in effective mean stress. The strain-hardening phase occurs at relatively low strains, until the peak strength of the sample is achieved and continuous fractures are formed (point 3 in Fig. 3b).

**Figure 4:**
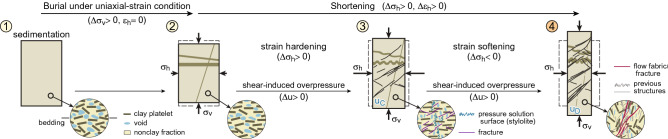
Evolution of the shale fabric. Schematic variation in an idealized shale sample of fabric, structures, main stresses, and strain during evolution from sedimentation (1) to critical state (4). Numbers correspond to the stages differentiated in Fig. 3. Within the circles, it is schematized the shale fabric including the orientation of the clay platelets, a possible geometry of voids, different fractures, and stylolites that are due to dissolution and pressure solution processes. Associated, sub-perpendicular dilatant micro-structures and fractures are omitted for the sake of clarity (Fig. 5c). These sketches illustrate common observations in deformed shales, like how: (i) clay platelets orientation with respect to bedding and the void space change progressively during consolidation and deformation (1 to 3)^25, 66^, (ii) fractures are initiated in domains with organic-matter particles^67﻿]^ and authigenic quartz cements^68^ (3), and (iii) the previous fabric in sample is finally disrupted and a new set of anastomosing shear zones and fractures are formed when rock achieves critical state (4).

The second stage, after peak strength, consists of strain-weakening behaviour (path 3 to 4, Fig. 3b), during which deviatoric stress (q) decreases. Softening is attributed to penetrative fracturing and the collapse of rock fabric or breakage of cementation/bonds, also known as destructuration^64^ (Fig. 4c). Destructuration entails elevated rock compression, which, in undrained conditions, translates into a significant increase in pore pressure (u_D_, Fig. 3a**–**b) and decrease in effective mean stress^54, 65^.

The clay reaches a state where stresses almost stop changing (point 4, Figs. 3, 4), even though the sample is still being shortened and deformed. This is the critical state, at which unlimited (plastic) shear deformation occurs without any changes in stresses or volume^53, 69, 70^ (plateau, Fig. 3b).

In a perfectly homogeneous medium, materials at the critical state flow everywhere, destroying all material fabrics and cement. In the real world, geologic materials are never perfectly homogeneous. Weak zones (like organic-rich domains and regions with authigenic quartz cements^68, 67^) fail first, producing an anastomosing network of highly sheared rock surrounding lenses in which earlier structures are preserved^5, 6, 71, 72^ (Fig. 4).

Vane tests show that at critical state the clay flow is viscous, that is, the strain rate at critical state varies with shear stress (Fig. s6 in Supplementary Information 2). As such, the clay behaves as a Herschel–Bulkley material: it is solid (no flow) when shear stress is smaller than the static shear strength, and when shear stress exceeds the strength, it flows as a fluid material at a strain rate that increases with the excess shear stress (Fig. 2).

Destructuration of sedimentary and tectonic fabrics, breaking of cement, large shear deformation, high overpressure, and plastic flow associated with the critical state tie this state to mobile shales. We therefore propose that mobile shales are at the critical state and suggest the following definition for mobile shales: “bodies of clay-rich sediment or sedimentary rock undergoing penetrative, (visco-) plastic deformation at the critical state”.

## Implications of a critical-state model for mobile shales

### Fluid-supported versus grain-supported flow

The critical-state model can explain not only the viscous-plastic behaviour of shales in structures such as shale anticlines and diapirs (Fig. 1c‒e), but also the initiation of the fluid-like behaviour of shales in mud volcanoes. At critical-state, all cements, bonds, and sedimentary and tectonics fabrics are destroyed by pervasive shearing, and the material flow is purely frictional (failure envelope has no cohesion and passes through the origin in a p′–q diagram). Thus, when pore pressure increases in mud volcanoes so much as to bring the effective stresses to zero, the grains lose contact, and the shear strength becomes zero (Figs. 3 and s2 in Supplementary Information 2). In this case, the Herschel–Bulkley behaviour converges to a viscous-fluid model. In this case, the shale is mobile even at small shear stresses, which corresponds to the behaviour of the fluidized^73^ shale in mud volcanoes (further details in Supplementary Information 2 and Fig. s6).

The drop in effective stress leading to fluidization of shales in mud volcanoes may result from an increase in fluid pressure or a drop in total confining stress. One important scenario producing fluidization occurs when mobile shales rise up a fracture system below a mud volcano^74^. Total confining stress decreases as the material approaches the surface. This drop in total stress during rise leads to gas phase exsolution (e.g., methane and CO_2_) in organic-rich shales, for example, elevating the pore pressure and promoting fracturing^8, 22, 75^. This combination can bring effective stresses to zero, leading to the highly fluidized *ejecta* sourced from mud volcanoes.

### Brittle versus ductile behaviour

**Figure 5:**
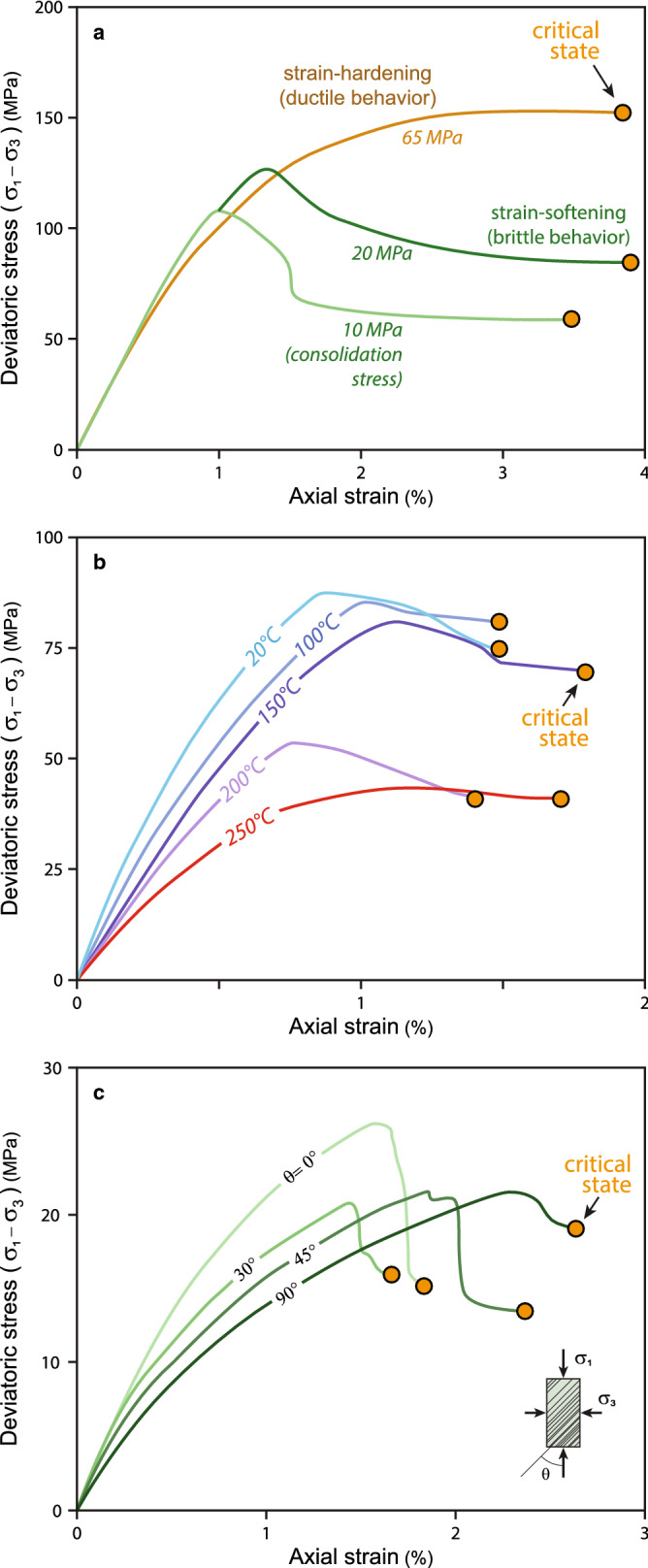
Experimental stress–strain curves and critical-state conditions in shales. (**a**) Mudstone samples from Kuqa Depression (Tarim Basin, China) deformed at different consolidation stresses^78^. Brittle behaviour characterized by strain-softening (experiments at consolidation stresses of 10 and 20 MPa), ductile behaviour showing no difference between peak and residual strengths (consolidation stress of 65 MPa). Tests with compression perpendicular to bedding. (**b**) Stress–strain curves for Tournemire shale (Massif Central, France) deformed at different temperatures^77^. Tests under constant consolidation stress (20 MPa) and compression perpendicular to bedding. (**c**) Stress–strain curves for Pierre-1 shale (USA) deformed with different orientations (θ) of bedding^79^. Tests under constant consolidation stress (25 MPa). Other symbols like in Fig. 3. Detailed information about these experimental tests and about shale samples in Supplementary Information 2 (Tables s2–s3). Additional experiments documenting how confining pressure, temperature, and fabric anisotropy affect geomechanical behaviour of shales in Figs. s3, s4, and s5, respectively (Supplementary Information 2).

There is a longstanding debate concerning the relative importance of brittle and ductile behaviour in mobile shales^4, 5, 7, 76^. Brittle behaviour is seen in a stress–strain plot with a strain-softening behaviour; i.e., residual strength at the critical state is significantly lower than peak strength (Figs. 3b, 5a). Conversely, ductile behaviour is associated with strain hardening, having similar residual and peak strengths (Fig. 5). The critical-state model suggests that shales with either behaviour can reach the critical state (plateau at the end of the curves) and become mobile (Fig. 5a). In Fig. 3, for example, the sample reaches the critical state through brittle behaviour. A shale can behave in a brittle or ductile way, depending in part on confining stress, temperature, and strain rate^42, 77, 78^ (Figs. 5a,b and s3–s4 in Supplementary Information 2).

Where shales have reached the critical state through a brittle behaviour, any brittle structures formed during approach to the critical state may be preserved and severely reworked in lenses between anastomosing shear zones during critical-state flow, resulting in a mixture of structural styles^5, 6, 15, 72^.

### Pore-fluid pressure

Low velocity of mobile shales suggests overpressure is high in these shales^4, 7, 9–11, 13, 14, 30, 31, 45–52^. The critical-state-model provides insight into the role that overpressure plays in shale mobilization. In principle, it is possible to mobilize shales at any pore pressure by purely increasing shear stress to reach the shear strength (see the critical state line in the p′–q diagram in Fig. 3b). However, increase in pore pressure decreases effective confining (mean) stress (p′), making it possible to reach critical state at lower shear stress. Without high overpressure, forces driving shear stress in mobile shales may not be enough to bring the shales to critical state and make them mobile. This explains the observed correlation between shale mobility and high pore pressures.

Several sources have been suggested to increase pore pressure in mobile shales, including disequilibrium compaction or generation of hydrocarbons^48, 62, 47^. The critical state model suggests shear-induced overpressure as another mechanism for increasing pore pressure in mobile shales as they deform toward critical state (cf. blue curve in Fig. 3c, and Figs. s2–s4 in Supplementary Information 2). This is because, under undrained conditions, any increase in either deviatoric or mean stress promotes rock compaction and as trapped fluids are incompressible, they must bear the load^48, 47^.

### Degree of consolidation

**Figure 6:**
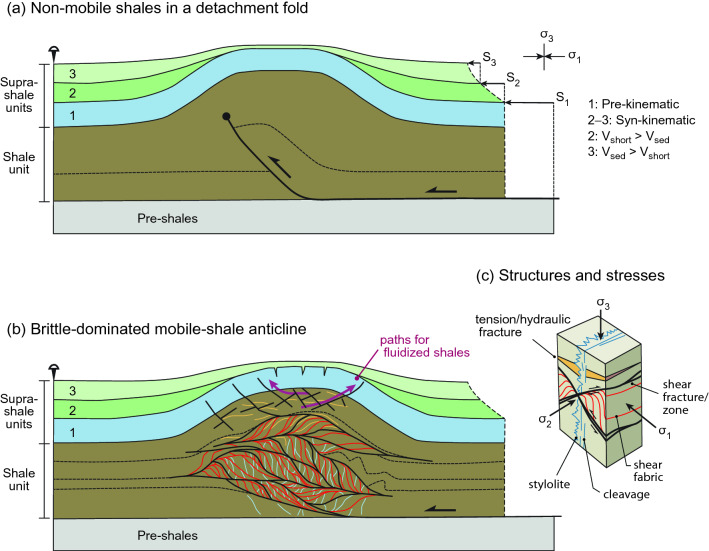
Structural sketch to differentiate mobile shales in a shale-cored anticline. (**a**) Detachment fold involving non-mobile shales. Notice that deformation occurs along a single fault surface, and there is a limited amount of shale inflation in the fold core. (**b**) Mobile shales originated in the core of a similar detachment fold. In this case deformation at critical state is mostly accommodated by penetrative brittle deformation, producing a large shale inflation in the anticline. A large variety of structures accompanies mobile shales, like parasitic folds, anastomosing shear zones and fractures, cleavage and stylolites in the inner core, and conjugate shear fractures in the outer arc of the shale fold. It is also indicated the possible pathways for fluidized shales to pierce the supra-shale sequences finally forming extruded shale sheets and mud volcanoes^74^ (Fig. 1a–c). (**c**) Scheme showing the simplified geometry of the main structures (faults, foliations, and hydraulic fractures^80^) and their orientation with respect to principal stresses. Shortening occurs by horizontal compression after the deposition of the layer no. 1. The general geometry of the syn-tectonic sequences (layers 2 and 3) when shortening rate (V_short_) is larger than sedimentation rate (V_sed_) or vice versa coinciding with deposition of layers no. 2 and 3, respectively, is also shown schematically.

The critical-state concept can explain mobilization of both consolidated and unconsolidated materials^55–57, 59–61^. Consolidated or cemented shales have a higher shear strength than unconsolidated materials, particularly when they are compressed parallel to the fabric or stratification^79^ (Figs. 6c and s5 in Supplementary Information 2). It thus takes higher shear stress to break cements and drive these shales to the critical state. However, once this disaggregation occurs, critical-state flow can occur just as in any other shale.

In contrast to previous interpretations^13^, critical-state mechanics suggests that there is no depth or temperature limit on the formation of mobile shales (Figs. 6 and s2–s4 in Supplementary Information 2), Even if cementation has occurred, mobilization in deep shales remains possible—it just takes a higher shear stress to overcome shale strength.

### End of shale mobility

In many areas, stratal patterns on seismic data suggest that some formerly mobile shales became later inactive (Figs. 1c–e). The critical-state model provides an explanation for stabilization of formerly mobile shales. According to our model, mobile shales stop mobility when shear stress becomes smaller that the shear strength (Fig. s6 in Supplementary Information 2). This may occur through either a drop in shear stress (e.g., by the ending or lessening of the tectonic activity) or an increase in shear strength (e.g., by consolidation due to pore fluid drainage or cementation due to diagenetic transformations in shale); in either case the shale would depart from critical state (Figs. 3, 5). For example, in shales that have become mobile due to regional shortening, shear stresses may drop if shortening stops.

### Seismic properties

The critical-state model also gives insight into seismic properties of mobile shales. First, it predicts that overpressure increases in mobile shales due to shear deformation^48^ (Figs. 2 and s2–s4 in Supplementary Information 2) and seismic velocity thus decreases, which affects the seismic impedance of the shales^14^. Second, shear stiffness of a material drops significantly approaching the critical state. Therefore, our model predicts that mobile shales should have a lower S-wave velocity than immobile shales at the same porosity^30, 31, 81^. Third, although flow at the critical state destroys preexisting rock fabrics, it may create new flow fabrics^5, 6, 71, 72^. Seismic anisotropy is therefore affected.

An important consideration in seismic imaging of mobile shales is whether the shales are presently mobile—that is, whether they are presently at the critical state. Once shales leave the critical state, overpressures and shale stiffness may return to normal values. However, any changes in anisotropy will remain.

To illustrate the application of the critical-state concept to a particular structure, Fig. 6 schematizes how the deformation would occur throughout the fold core of an anticline with mobile shales (Fig. 6b), in contrast to the same fold formed by slip along a discrete fault without any shale mobility (Fig. 6a). The overall fold geometry is the same, but the occurrence in the fold core of a penetrative deformation, the superposition of brittle and ductile structures, the abundance of tensional fractures and veins (formed possibly by hydraulic fracturing^80^; Fig. 6c), the notable inflation of the shale layer, together with the possible occurrence of extruded mud flows above the fold crest^74^, are indicative of mobile-shales deforming at critical state.

## Conclusions

We propose that the critical-state model is a viable hypothesis for the mechanical behaviour of mobile shales. This proposal helps to answer many of the key questions related to mobile shales:

- *Is deformation in mobile shales brittle, ductile, or both?* Prior to reaching critical state and becoming mobile, shales can experience either brittle or ductile deformation. At critical state, flow in shales generates an anastomosing network of highly-sheared material, surrounding lenses in which previous structures are preserved. Thus, both brittle and ductile structures may exist in mobile shales.
- *Can shales with diagenetic cements become mobile? Does depth of burial play a role in shale mobilization?* We have demonstrated that there is not a diagenetic threshold for shale mobility. Deeper, more consolidated and cemented shales can achieve critical state and flow, although it takes higher shear stresses to break cements and drive these shales to the critical state.
- *Do all mobile shales have to be highly overpressured?* In theory, it is possible to reach the critical state purely through an increase in shear, without any increase in overpressure. However, critical state is much easier to reach if overpressures are present, because overpressures decrease the mean effective stress. This is consistent with information from seismic velocities, which suggest that most (if not all) mobile shales are overpressured.
- *How does shale mobilization affect its seismic properties?* The lack of internal seismic reflectivity in mobile shales is consistent with destructuration of the sedimentary and tectonic fabrics in shales due to large shear deformation and plastic flow associated with the critical state.
- *Why do some mobile shales become immobile?* Mobile shales stop mobility when the shear stress drops below the shear strength, and the shale departs from critical state. This may occur through either a drop in shear stress (by the ending or lessening of the load driving the shear stress in shales; e.g., by the end of tectonic activity) or an increase in shear strength (e.g., by consolidation due to pore fluid drainage or cementation due to diagenetic transformations).

We believe that this model offers many exciting prospects for future research. We look forward to seeing tests of this hypothesis as the study of mobile shales advances into the future.

## Supplementary Information

Supplementary Information.
