# Author Correction: Proposal for a mechanical model of mobile shales

**DOI:** 10.1038/s41598-022-06170-2

**Published:** 2022-01-31

**Authors:** Juan I. Soto, Mahdi Heidari, Michael R. Hudec

**Affiliations:** 1 Bureau of Economic Geology, Jackson School of Geosciences, The University of Texas at Austin, University Station, Box X, Austin, TX 78713-8924 USA; 2 Departamento de Geodinámica, Universidad de Granada, Avenida de Fuente Nueva s/n, 18071 Granada, Spain

Correction to: *Scientific Reports* 10.1038/s41598-021-02868-x, published online 10 December 2021

The original version of this Article contained an error in the legend of Figure 1.

“(**a**) Garadagh mud volcano in onshore Azerbaijan (40.24°N and 49.51°E).”

now reads:

“**(a)** Otmanbozdag mud volcano (within the Garadagh structure) in onshore Azerbaijan (40.24°N and 49.51°E).”

The original Article has been corrected.

