# Supplementary material for: Proposal for a mechanical model of mobile shales
Source: Sci Rep. 2021 Dec 10;11:23785. doi: 10.1038/s41598-021-02868-x (PMC8664881; doi:10.1038/s41598-021-02868-x)
Supplement: Supplementary file 1 — Supplementary Information. [file 41598_2021_2868_MOESM1_ESM.pdf]

SCIENTIFIC REPORTS, NATURE

**Supplementary Material**

# PROPOSAL FOR A MECHANICAL MODEL OF MOBILE SHALES

**Juan I. Soto[(1,2) (*)], Mahdi Heidari[(1)], and Michael R. Hudec[(1)]**

(1) Bureau of Economic Geology, Jackson School of Geosciences, The University of Texas at Austin, Austin, TX, USA

(2) On leave of absence from: Departamento de Geodinámica, Universidad de Granada, Avenida de Fuente Nueva s/n, 18071 Granada, Spain

(*) Contact information:
Juan I. Soto
Bureau of Economic Geology
The University of Texas at Austin
10100 Burnet Rd., Bldg 130
Austin, TX 78758
Email: juan.soto@beg.utexas.edu

*Contents:*

**Supplementary Information 1.** Global distribution of mobile-shale structures …. *(page 2)*

Figure s1, Table s1, and supplementary references

**Supplementary Information 2.** Composition and geomechanical-test conditions in shales …. *(page 36)*

Tables s2−s3, Figures s2−s6, and supplementary references

*(Supplementary Information 1)*

# 1. Global Distribution of Mobile−Shale Structures – Tectonic Settings and References

*Contents:*

Figure s1 ׀ Global distribution of mobile-shale structures according to tectonic setting …. *(page 3)*
Table s1 ׀ List of regions with mobile shales …. *(page 4)*
Supplementary references for regions with mobile shales …. *(page 7)*

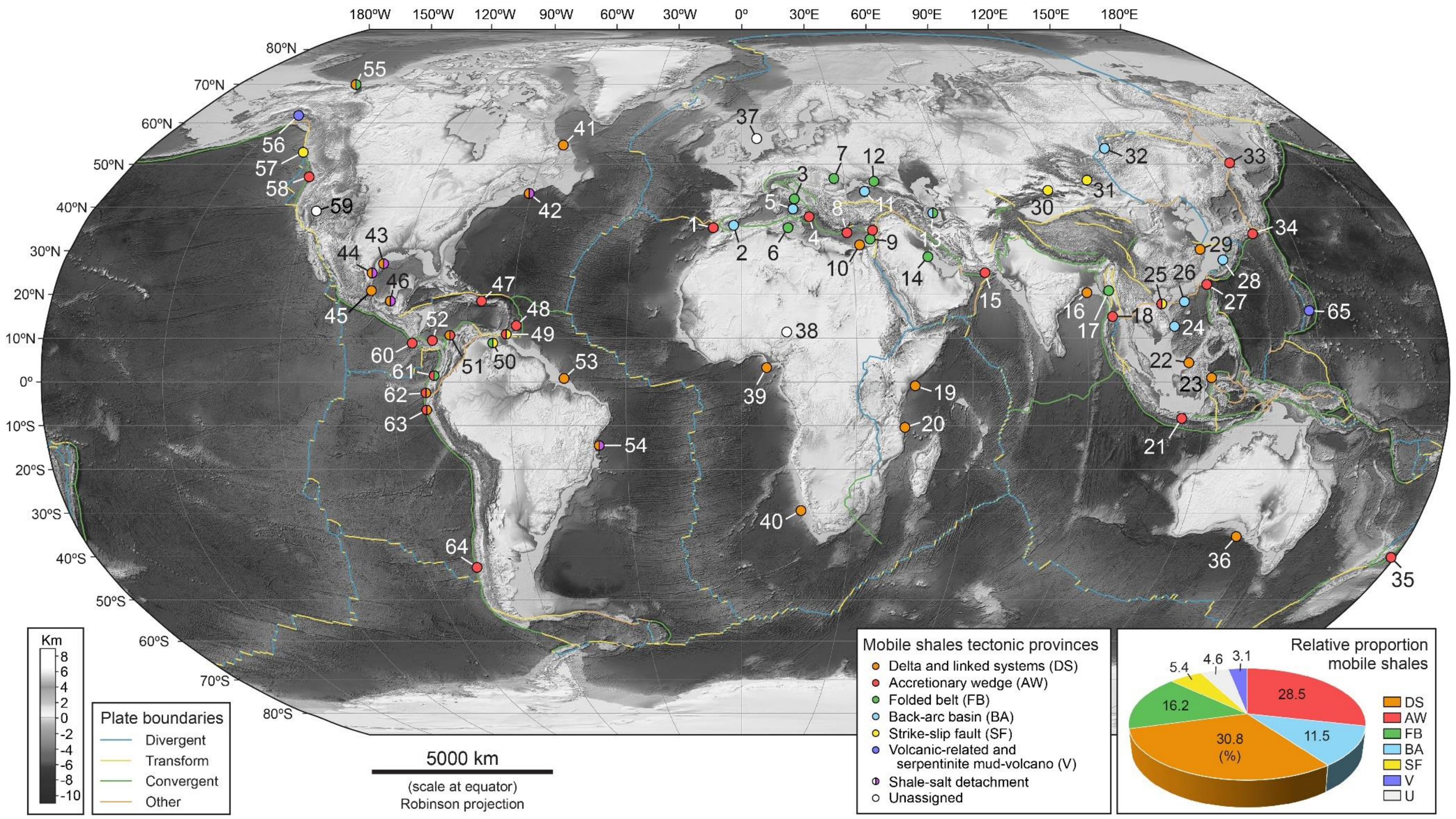


**Fig. s1 ׀ Global distribution of mobile-shale structures according to tectonic setting.** Numbers refer to geographic areas listed in this document (Table s1). Inset pie diagram shows relative proportions (in %) of mobile-shale structures in different tectonic settings (for total population of 65 regions or categories). Plate boundaries according to Bird (2003) and USGS (2020). Topographic elevation using GEBCO (2020) (dataset with 15 arc-second global grid). Map plotted with Robinson projection.

**Table s1 |** List of regions with mobile shales.

| No. | Region | Tectonic Setting (1) | |
|---|---|---|---|
| 1 | Gulf of Cadiz | AW | |
| 2 | Alboran Sea (W Mediterranean) | BA | |
| 3 | Appenines System (Italy and Sicily) | FB | |
| 4 | Calabrian Arc | AW | |
| 5 | Tyrrhenian Sea | BA | |
| 6 | Offshore Tunisia | FB | |
| 7 | Carpathians and Dacian Basin (Romania) | FB | |
| 8 | Eastern Mediterranean Ridge | AW | |
| 9 | Levant Basin (Including Latakia and Cyprus Ridges) | FB | AW |
| 10 | Nile Delta | DS | |
| 11 | Black Sea | BA | |
| 12 | Sea of Azov and Crimea Peninsula | FB | |
| 13 | South Caspian Sea (Azerbaijan, N Iran and Turkmenistan) | BA | FB |
| 14 | Burgan Arch (Kuwait) | FB | |
| 15 | Makran Accretionary Wedge (Onshore and Offshore Iran and Pakistan) | AW | |
| 16 | Ganges-Brahmaputra Delta (Offshore Bangladesh) | DS | |
| 17 | Burma Arc | FB | |
| 18 | Andaman Basin (Offshore Myanmar and Thailand) | AW | FB |
| 19 | Javu-Lamu Basin (Offshore S Somalia-N Kenia) | DS | |
| 20 | Rovuma Delta (Offshore N Mozambique-S Tanzania) | DS | * |
| 21 | Banda Arc Orogen (Java to Sumba and Timor) | AW | FB |
| 22 | Baram Delta (NW Brunei And Sarawak) | DS | |
| 23 | Mahakam Delta (SE Indonesia) | DS | |
| 24 | Zhongjiannan Basin (Offshore Vietnam) | BA | |
| 25 | Yinggehai-Hanoi Depression (South China Sea and Offshore Vietnam) | AW | SF |
| 26 | Xisha Trough and Pearl River Mouth Basin (South China Sea, SE Hainan Island) | BA | |
| 27 | Manila Trench (SW Taiwan) | AW | |
| 28 | Okinawa Trough (East China Sea) | BA | |

**Table s1** (continuation) ❘ List of regions with mobile shales.

| No. | Region | Tectonic Setting (1) | |
|---|---|---|---|
| 29 | Yangtze Delta (E China Sea) | DS | |
| 30 | Junggar Basin (Onshore China) | SF | FB |
| 31 | Gobi Altay (Mongolia) | SF | FB |
| 32 | Lake Baikal | BA | |
| 33 | Okhotsk Sea (Sakhalin Island, E Russia) | AW | |
| 34 | Nankai Accretionary Wedge (Offshore Japan) | AW | |
| 35 | Hikurangi Accretionary Wedge (N New Zealand) | AW | |
| 36 | Ceduna Sub-Basin (Offshore S Australia) | DS | |
| 37 | North Sea (Håkon Mosby Mud Volcano) | U | |
| 38 | Upper Benue Trough (Onshore Nigeria) | U | |
| 39 | Niger Delta | DS | |
| 40 | Orange Basin (Offshore Namibia and South Africa) | DS | |
| 41 | Labrador Margin (Saglek and Hopedale Basins) and Baffin Bay Basin (E Canada) | DS | |
| 42 | Offshore Nova Scotia (Sable Subbasin, E Canada) | DS | Salt |
| 43 | Port-Isabel Folded Belt and Northern Gulf of Mexico | DS | Salt |
| 44 | Delta del Bravo (W Gulf of Mexico, USA and Mexico) | DS | Salt |
| 45 | Mexican Ridges (W Gulf of Mexico, Mexico) | DS | |
| 46 | Comalcalco, Macuspana and Salina del Istmo Basins (S Gulf of Mexico, Mexico) | DS | Salt |
| 47 | Puerto Rico and Hispaniola Accretionary Wedges | AW | |
| 48 | Barbados Accretionary Wedge | AW | |
| 49 | Trinidad and Tobago | AW | SF |
| 50 | Maturin Basin (Onshore Venezuela) | FB | SF |
| 51 | Magdalena and Orinoco Deltas. Sinu Folded Belt (Offshore N Colombia) | DS | AW |
| 52 | N Panamá Accretionary Wedge and Limón Folded Belt (N Panamá and Costa Rica) | AW | |
| 53 | Foz Do Amazonas, Barreirinhas and Pará-Maranhão Basins (Equatorial Margin, Brazil) | DS | |
| 54 | Camamu-Almada Basin (Brazil) | DS | Salt |

**Table s1** (continuation) | List of regions with mobile shales.

| No. | Region | Tectonic Setting (1) | |
|---|---|---|---|
| 55 | Beaufort-Mackenzie Basin (Offshore Artic Canada) | DS | FB |
| 56 | Wrangell Mountains (Onshore Alaska) | V | |
| 57 | The Queen Charlotte–Fairweather Fault System (Western British Columbia, Canada) | SF | |
| 58 | Cascadia Margin (W USA and Canada) | AW | |
| 59 | Salton Sea (Onshore California) | U | |
| 60 | Costa Rica-Nicaragua Accretionary Wedge (Pacific) | AW | |
| 61 | Tumaco Basin (Pacific Colombia) | AW | FB |
| 62 | Esperanza-Guayaquil Basin (Offshore Ecuador) | AW | DS |
| 63 | Talara-Tumbes Basin (Offshore Peru) | AW | DS |
| 64 | Chile Forearc (Offshore Chile) | AW | |
| 65 | Serpentinite Mud Volcanoes (Mariana Forearc) | V | |

Abbreviations and notes:

AW: Accretionary wedge
BA: Back-arc basin (including rifts and extensional regimes in other tectonic settings)
DS: Delta system, normally developing a linked-system (with updip marginal extension and downdip deepwater contraction)
FB: Fold-and-thrust belt (mostly as contractional folded belts in continental margins and other contraction-dominated margins, with few additional examples in continental orogenic systems)
Salt: Mobile-shale structures combined with salt (forming in most of the cases mixed detachment systems and complex diapiric structures)
SF: Strike-slip faulting regime (including transform faults)
U: Unassigned tectonic setting (due to unprovided information in the references or to highly-spectulative propositions)
V: mud volcanoes related to igneous volcanism and serpentinite-mud volcanoes in forearc settings

Numbers refer to the regions listed in the Supplementary References (see Figure s1).
(1) Main tectonic setting in the first column, which has been used to produce the pie plot in Figure s1. Second column includes secondary information about the tectonic conditions.
(*): Region with some discussion about the nature of the mobile layer (salt or mobile-shales).

## Supplementary references for regions with mobile shales

Hereafter, list of references grouped by numbered regions (Fig. s1 and Table s1) provided. Selected references document occurrence of mobile-shale structures in every region and detail their tectonic characteristics. Mobile-shale structures documented in these works range from mud volcanoes to different types of shale diapirs (Fig. 1). Information provided by these studies used to reconstruct tectonic regime dominating every region (Table s1) and to derive pie diagram shown in Figures 1 and s1.

### References for the map in Figure s1

Bird, P. An updated digital model of plate boundaries. Geochem. Geophys. Geosyst. 4, doi:10.1029/2001GC000252 (2003).

GEBCO Compilation Group. GEBCO 2020 Grid. doi:10.5285/a29c5465-b138-234d-e053-6c86abc040b9. (https://www.gebco.net/data_and_products/gridded_bathymetry_data/gebco_2020/, accessed in August, 2020) (2020)

USGS. Plate tectonic boundaries. (https://www.usgs.gov/media/files/plate-boundaries-kmz-file, accessed in October 2020) (2020)

### References describing mobile-shales structures according to numbered regions (Fig. s1 and Table s1)

### 1. Gulf of Cadiz

Depreiter, D., Poort, J., Van Rensbergen, P. & Henriet J. P. Geophysical evidence of gas hydrates in shallow submarine mud volcanoes on the Moroccan margin. *J. Geophys. Res.* **110**, B10103 (2005).

Díaz-del-Río, et al. Vast fields of hydrocarbon-derived carbonate chimneys related to the accretionary wedge/olistostrome of the Gulf of Cádiz. *Mar. Geol.* **195**, 177-200 (2003).

Foubert, A., et al. Carbonate mounds in a mud volcano province off north-west Morocco: Key to processes and controls. *Mar. Geol*., **248**, 74-96 (2008).

Gardner, J. M. Mud volcanoes revealed and sampled on the Western Moroccan continental margin. *Geophys. Res. Lett.* **28** 339-342 (2001).

Henriet, J. P., et al. The mud volcano province on the Atlantic Moroccan margin: towards a neutral laboratory for joint European-Maghreb research, in *Fluid Seepages—Mud Volcanism in the Mediterranean and Adjacent Domains* 21-26 (*CIESM Work. Mono.* **29**, 2005).

Hensen, C., et al. Strike-slip faults mediate the rise of crustal-derived fluids and mud volcanism in the deep sea. *Geology* **43**, 339-342 (2015)

Ivanov, M. K., et al. Goals and principal results of the TTR-9 cruise. *IOC/Unesco Work. Rep.* **168**, 3-4 (2000).

Judd, A. Gas emission from mud volcanoes: significance to global climate change, in *Mud Volcanoes, Geodynamics and Seismicity* (eds. Martinelli, G. & Panahi, B.) 147-157 (*NATO Sci. Ser. IV*, **51**, 2005).

Maignien, L., et al. Anaerobic oxidation of methane in a cold-water coral carbonate mound from the Gulf of Cadiz. *Int. J. Earth Sci.* (*Geol. Rundsch*.) (2010).

Martín-Puertas, C., et al. A comparative mineralogical study of gas-related sediments of the Gulf of Cádiz. *Geo-Mar. Lett.* **27** 223-235 (2007).

Matias, H., et al. Salt tectonics in the western Gulf of Cadiz, southwest Iberia. *AAPG Bulletin*, **95**, 1667-1698 (2011) doi:10.1306/01271110032.

Mazurenko, L. L., Soloviev, V. A., Gardner, J. M., & Ivanov, M. K. Gas hydrates in the Ginsburg and Yuma mud volcano sediments (Moroccan Margin): results of chemical and isotopic studies of pore water. *Mar. Geol.*, **195**, 201-210 (2003).

Murton, B. J. & Biggs, J. Numerical modelling of mud volcanoes and their flows using constraints from the Gulf of Cadiz. *Mar. Geol.*, **195**, 223-236 (2003).

Ovsyannikov, D. O., Sadekov, A. Y. & Kozlova, E. V. Rock fragments from mud volcanic deposits of the Gulf of Cadiz: an insight into the Eocene-Pliocene sedimentary succession of the basin. *Mar. Geol.* **195**, 211-221 (2003).

Palomino, D., et al. Multidisciplinary study of mud volcanoes and diapirs and their relationship to seepages and bottom currents in the Gulf of Cádiz continental slope (northeastern sector). *Mar. Geol.* **378**, 196-212 (2016).

Pinheiro, L., et al. Mud volcanism in the Gulf of Cádiz: results from the TTR-10 cruise. *Mar. Geol.* **195,** 131-151 (2003).

Pinheiro, L. M., et al. Structural control of mud volcanism and hydrocarbon-rich fluid seepage in the Gulf of Cádiz: recent results from the TTR-15 cruise, in *Fluid Seepages/Mud Volcanoes in the Mediterranean and Adjacent Domains* (eds. Mascle, J., Sakellariou, D., & Briand, F.) 53-58 (*CIESM Work. Mono.* **29**, 2006.

Pinheiro, L. M., et al. Seismic data: preliminary results of investigations during the TTR-15 of R.V. "Prof. Logachev" Cruise Report, in *Deep-Water Cold Seeps, Sedimentary Environments and Ecosystems of the Black and Thyrrenian Seas and the Gulf of Cadiz Intergovernmental Oceanographic Commission Technical Series*, 59-66 (*UNESCO Paris* **72**, 2007).

Schwenk, T., et al. Imaging mud volcanoes and tectonic structures in the Gulf of Cádiz—first seismic results of TTR15/4. Workshop Report. (*Intergov. Ocean. Comm.*, Moscow, **89**, 2006).

Somoza, L., et al. Seabed morphology and hydrocarbon seepage in the Gulf of Cádiz mud volcano area: acoustic imagery, multibeam and ultrahigh resolution seismic data. *Mar. Geol.* **195**, 153-176 (2003).

Somoza, L., & MVSEIS_08 Team. New discovery of mud volcanoes related to active strike-slip faults and thrusting ridges in the Moroccan margin (Gulf of Cadiz, Eastern Central Atlantic), in *9th International Conference on Gas in Marine Sediments, Bremen* (2008).

Vanneste, H., et al. Spatial variation in fluid flow and geochemical fluxes across the sediment-seawater interface at the Carlos Ribeiro mud volcano (Gulf of Cadiz). *Geochim. Cosmochim. Acta*, **75**, 1124-1144 (2011).

Van Rensbergen, P., et al. The El Arraiche mud volcano field at the Moroccan Atlantic slope, Gulf of Cadiz. *Mar. Geol.*, **219**, 1-17 (2005).

## 2. Alboran Sea (W Mediterranean)

Blinova, V. N., et al. Active mud volcanism in the West Alboran Basin: Geochemical evidence of hydrocarbon seepage. *Mar. Petr. Geol.* **28**, 1483-1504 (2011).

Comas, M. C., García-Dueñas, V., & Jurado, M. J. Neogene tectonic evolution of the Alboran Basin from MCS data. *Geo-Mar. Lett.* **12**, 157-164 (1992).

Comas, M. C., Platt, J. P., Soto, J. I., & Watts, A. B. The origin and tectonic history of the Alboran Basin: insights from ODP Leg 161 results, in *Proceedings of Ocean Drilling Program* (eds. Zahn, R., Comas, M. C. & Klaus, A.) 555-580 (*Scientific Results* **161**, 1999).

Comas, M. C., Soto, J. I., Talukder, A. R. & TTR-12 Leg 3 (MARSIBAL 1 Scientific Party). 20Discovering active mud volcanoes in the Alboran Sea, western Mediterranean), in Geological and Biological Processes at Deep-Sea European Margins and Oceanic Basins (eds. Marani, M., Akhmanov, G. & Suzyumov A.) *Intergov. Ocean. Comm. Work. Rep.* **187**, 14-16 (2003).

Fernández-Ibánez, F., & Soto, J. I. Pore pressure and stress regime in a thick extensional basin with active shale diapirism (western Mediterranean). *AAPG Bull*., **101**, 233-264 (2017).

Gennari, G., et al. Sedimentary sources of the mud-breccia and mud volcanic activity in the Western Alboran Basin. *Mar. Geol.*, **339**, 83-95 (2013).

Jurado, M. J., & Comas, M. C. Well log interpretation and seismic character of the Cenozoic sequence in the northern Alboran Sea. *Geo-Mar. Lett.*, **12**, 129-136 (1992).

López-Rodríguez, C., et al. Recent, deep-sourced methane/mud discharge at the most active mud volcano in the western Mediterranean. *Mar. Geol.*, **408**, 1-17 (2019).

Margreth, S., et al. Growth and demise of cold-water coral ecosystems on mud volcanoes in the West Alboran Sea: the messages from the planktonic and benthic foraminifera. *Mar. Geol.*, **282**, 26-39 (2011).

Morley, C. K., Notes on Neogene basin history of the western Alboran Sea and its implications for the tectonic evolution of the Rif-Betic orogenic belt. *Afr. J. Earth Sci.*, **14**, 57-65 (1992).

Pérez-Belzuz, F., Alonso, B. & Ercilla, G. History of mud diapirism and triggering mechanisms in the western Alboran Sea. *Tectonophysics*, **282**, 399-423 (1997).

Sautkin, A., et al. Mud volcanoes in the Alboran Sea: evidence from micropaleontological and geophysical data. *Mar. Geol.*, **195**, 237-261 (2003).

Somoza, L., et al. Structure of mud volcano systems and pockmarks in the region of the Ceuta contourite depositional system (Western Alborán Sea). *Mar. Geol.*, **332-334**, 4-26 (2012).

Soto, J. I., Fernández-Ibáñez, F. & Talukder, A. R. Recent shale tectonics and basin evolution of the NW Alboran Sea. *Lead. Edge* **31**, 768-775 (2012).

Soto, J. I., Fernández-Ibáñez, F., Talukder, A. R., Martínez-García, P. Miocene shale tectonics in the northern Alboran Sea, Western Mediterranean, in *Shale Tectonics* (ed. L. Wood) 119-144 (*AAPG Mem.* **93**, 2010).

Talukder, A. R., Comas, M. C. & Soto, J. I. Pliocene to Recent mud diapirism and related mud volcanoes in the Alboran Sea, western Mediterranean, in *Subsurface Sediment Mobilization* (eds. Van Rensbergen, P., Hills, R. R., Maltman, A. & Morley, C.) 443-459 (*Geol. Soc., London, Spec. Pubs.* **216,** 2003).

## 3. Appenines System (Italy and Sicily)

Accaino, F., et al. Fluid seepage in mud volcanoes of the northern Apennines: an integrated geophysical and geological study. *J. Appl. Geophys.* **63**, 90-101 (2007).

Bonini, M. Elliptical mud volcano caldera as stress indicator in an active compressional setting (Nirano, Pede-Apennine margin, northern Italy). *Geology* **36**, 131-134 (2008).

Bonini, M. Interrelations of mud volcanism, fluid venting, and thrust-anticline folding: examples from the external northern Apennines (Emilia-Romagna, Italy). *J. Geophys. Res.* **112**, B08413 (2007).

Bonini, M. Mud volcano eruptions and earthquakes in the Northern Apennines and Sicily, Italy. *Tectonophysics* **474**, 723-735 (2009).

Capozzi, R. & Picotti, V. Fluid migration and origin of a mud volcano in the Northern Apennines (Italy): the role of deeply rooted normal faults. *Terra Nova* **14**, 363-370 (2002).

Capozzi, R. & Picotti, V. Genesis of cold seeps and mud volcanoes of the Northern Apennine foothills, in *Fluid Seepages/Mud Volcanism in the Mediterranean and Adjacent Domains,* 59-64 (*Comm. Int. pour l'Expl. Sci. de la Mer Work. Mono.* **29**, 2006).

Capozzi, R. & Picotti, V. Spontaneous fluid emissions in the Northern Apennines: geochemistry, structures and implications for the petroleum system, in *Hydrocarbons in Contractional Belts* (eds. Goffey, G. P., Craig, J., Needham, T. & Scott, R.) 115-135 (*Geol. Soc., London, Spec. Pubs.* **348**, 2010).

Carminati, E., Scrocca, D. & Doglioni, C. Compaction-induced stress variations with depth in an active anticline: Northern Apennines, Italy. *J. Geophys. Res.,* **115**, B02401 (2010).

Clari, P., Cavagna, S., Martire, L., & Hunziker, J., A Miocene mud volcano and its plumbing system: a chaotic complex revisited (Monferrato, NW Italy). *J. Sed. Res.* **74**, 662-676 (2004).

Etiope, G., et al. Methane emission from the mud volcanoes of Sicily (Italy). *Geophys. Res. Lett.* **29**, 1215, (2002).

Etiope, G., Martinelli, G., Caracausi, A. & Italiano, F. Methane seeps and mud volcanoes in Italy: Gas origin, fractionation and emission to the atmosphere. *Geophys. Res. Lett.* **34**, L14303 (2007).

Klasek, S. A., et al. Deep-sourced fluids from a convergent margin host distinct subseafloor microbial communities that change upon mud flow expulsion. *Front. Microbiol.* **10**, article 1436.

Martinelli, G., Mud volcanoes of Italy: a review. *Giorn. di Geol.*, 3rd series, **61**, 107-113 (1999).

Martinelli. G., & Judd. A. Mud volcanoes of Italy. *Geol. J.* **39**, 49-61 (2004) doi:10.1002/gj.943.

Morlotti, E., et al. Chaotic deposits from the external Calabrian Arc (Ionian Sea, eastern Mediterranean). *Mem., Soc. Geol. Ital*., **24**, 261-275 (1982).

Panieri, G., et al. Mud volcanoes along the inner deformation front of the Calabrian Arc accretionary wedge (Ionian Sea). *Mar. Geol.*, **336**, 84-98 (2013).

Rainone, M. L., Rusi, S., & Torrese, P. Mud volcanoes in central Italy: subsoil characterization through a multidisciplinary approach. *Geomorphology*, **234**, 228-242 (2015).

## 4. Calabrian Arc

Ceramicola, S., et al. Seafloor distribution and last glacial to postglacial activity of mud volcanoes on the Calabrian accretionary prism, Ionian Sea. *Geo-Mar. Lett.* **34**, 111-129 (2014).

Fusi, N., Savini, A. & Corselli, C. Evidence of mud diapirism and coral colonies in the Ionian Sea (Central Mediterranean) from high resolution chirp sonar survey. *Ann. Geophys*. **49**, 751-765 (2006).

Holland, C. W. G. et al. Mud volcanoes discovered offshore Sicily, *Mar. Geol.* **199**, 1-6 (2003).

Loher, M., et al. Mud volcanism in a canyon: morphodynamic evolution of the Active Venere Mud Volcano and its interplay with Squillace Canyon, Central Mediterranean. *Geochem., Geophys., Geosys.,* **19**, 356-378 (2018).

Praeg, D., et al. Tectonically-driven mud volcanism since the late Pliocene on the Calabrian accretionary prism, central Mediterranean Sea. *Mar. Petr. Geol.,* **26**, 1849-1865 (2009).

Savini, A., et al. Shallow seep-related seafloor features along the Malta plateau (Sicily channel—Mediterranean Sea): morphologies and geo-environmental control of their distribution. *Mar. Petr. Geol.*, **26**, 1831-1848 (2009).

## 5. Tyrrhenian Sea

Gamberi, F. & Rovere, M. Mud diapirs, mud volcanoes and fluid flow in the rear of the Calabrian Arc Orogenic Wedge (southeastern Tyrrhenian sea). *Bas. Res.*, **22**, 452–464 (2010).

## 6. Offshore Tunisa

Bedir, M. New seismic Neogene clay diapirs and the hydrocarbon implications in the North-Eastern African Margin of Tunisia, in *Mud Volcanoes, Geodynamics and Seismicity* (eds. Martinelli, G. & Panachi, B.) 1-15 (Springer 2005).

## 7. Carpathians and Dacian Basin (Romania)

Baciu, C., Caracausi, A., Etiope, G. & Italiano, F. Mud volcanoes and methane seeps in Romania: main features and gas flux. *Ann. Geophys.* **50**, 501-511 (2007).

Baciu, C., & Etiope, G. Mud volcanoes and seismicity in Romania, in *Mud Volcanoes, Geodynamics and Seismicity* (eds. Martinelli, G. & Panahi, B.) 77-87 (Springer, 2005)

Baciu, C., Ionescu, A. & Etiope, G. Hydrocarbon seeps in Romania: gas origin and release to the atmosphere. *Mar. Petr. Geol.* **89**, 130-143 (2018).

Brustur, T., Stănescu, I., Macaleţ, R., & Melinte-Dobrinescu, M. C. The mud volcanoes from Berca: A significant geological patrimony site of the Buzău Land Geopark (Romania). *Geo-Eco-Marina* **21**, 1-22 (2015).

Dicea, O. The structure and hydrocarbon geology of the Romanian East Carpathian border from seismic data. *Petr. Geosci.* **1/2**, 135-143 (1995).

Dicea, O. Tectonic setting and hydrocarbon habitat of external Carpathian basins in Romania, in *Structure and Prospects of Alpine Basins and Forelands*, *Peri-Tethys Memoirs 2*. (eds. Ziegler, V. A. & Horvath, E.) 403-425 (*Mém. du Mus. Nat. d'Hist. Natur.* **170**, 1996).

Dicea, O. & Enachescu, M. E. The hydrocarbon past, present, and future of Romania—the world's first oil producer. *Lead. Edge* **March**, 321-324 (2000).

Dicu, A. *Vulcanii Noroiosi din Zona Buzăului (The Mud-Volcanoes of the Buzău Region)* (ed. Victor, V. B.) (Bucuresti, 2005).

Doglioni, G., et al. Structural evolution of the eastern Balkans (Bulgaria). *Mar. Petr. Geol.*, **13**, 225-251 (1996).

Engulescu, P. Câteva Gloduri (ochiuri) din podişul Moldovei dintre Prut şi Siret (Some mud-volcanoes within the Moldova plateau between Prut and Siret valleys). *Inst. Geol. Rom.*, **II**, 133-142 (1911).

Etiope, G., et al. Gas flux to the atmosphere from mud volcanoes in Eastern Romania. *Terra Nova* **16**, 179-184 (2004).

Etiope, G., Baciu, C. L., & Schoell, M. Extreme methane deuterium, nitrogen and helium enrichment in natural gas from the Homorod seep (Romania). *Chem. Geo.* **280**, 89-96 (2011).

Frunzeti, N., Baciu, C., Etiope, G., & Pfanz, H., Geogenic emission of methane and carbon dioxide at Beciu mud volcano (Berca-Arbanasi hydrocarbon-bearing structure, eastern Carpathians, Romania). *Carpath. J. Earth. Env.* **7**, 159-166 (2012).

Jipa, D., Dinu, C. & Marinescu, N. Sedimentological significance of subsurface date in the western Dacian basin (upper Neogene, Romania): sedimentary environments, genetic sequence, basinal evolution. *Geo-Eco-Marina* **4**, 147-153 (1999).

Matenco, L., & Bertotti, G. Tertiary tectonic evolution of the external East Carpathians (Romania), *Tectonophysics* **316**, 255-286 (2000).

Nikishin, A.M., et al. Scythian platform, Caucasus and Black Sea region: Mesozoic–Cenozoic tectonic and dynamics, in *Stratigraphy and Evolution of Peri-Tethyan Platforms*, 177 (eds. Crasquin-Soleau, S., & Barrier, E.) 163-176 (*Mem. du Mus. Nat. d'Hist. Natur., Peri-Tethys Memoir* **3**, 1998).

Nikishin, A.M., et al. Mesozoic and Cenozoic evolution of the Scythian Platform–Black Sea–Caucasus domain, in *Peri-Tethys Memoir 6: Peri-Tethyan Rift/Wrench Basins and Passive Margins* (eds. Ziegler, P. A., Cavazza, W., Robertson, A. H. F. & Crasquin-Soleau, S.) 295-346 (*Mem. du Mus. Nat. d'Hist. Natur.* **186**, 2001).

Popescu, B. M. Romania's petroleum systems and their remaining potential. *Petr. Geosci.*, **1**, 337-350 (1995).

Roure, F., Rocca, E. & Sassi, W. The Neogene evolution of the Outer Carpathian flysch units (Poland, Ukraine and Romania): kinematics of a foreland fold-and-thrust belt. *Sed. Geol.*, **86**, 177-203 (1992).

Schniukov, E. F., et al. Mud-volcanoes of Romania. Preliminary data on the mineralogy of Paclele Mari and Paclele Mici Mud-Volcanoes. *Geo-Eco-Marina*, **15**, 131-137 (2009).

Spulber, L., et al. Methane emissions from natural gas seeps and mud volcanoes in Transylvania (Romania). *Geofluids* **10**, 463-475 (2010).

Tari, G., et al. Cimmerian and Alpine stratigraphy and structural evolution of the Moesian Platform (Romania, Bulgaria), in Regional and petroleum geology of the Black Sea and surrounding region. *AAPG Mem.* **68**, 63-90 (1997).

## 8. Eastern Mediterranean Ridge

Akhmanov, G. G. & Woodside, J. M. Mud volcanic samples in the context of Mediterranean Ridge Mud Diapiric Belt, in *Proc. ODP Sci. Results* **160** (eds. Robertson, A. H. F., Emeis, K. C., Richter, C. & Camerlenghi, A.) 597-605 (1998).

Belderson, R. H., Kenyon, N. H., & Stride, A. H. Local submarine salt-karst formation on the Hellenic outer ridge, Eastern Mediterranean. *Geology*, **6**, 716-720 (1978).

Brown, M., The nature and hydrogeologic significance of mud diapirs and diatremes for accretionary systems. *J. Geophys. Res.* **95**, 8969-8982 (1990).

Camerlenghi, A., et al. Geophysical evidence of mud diapirism on the Mediterranean Ridge accretionary complex. *Mar. Geophys. Res.* **17**, 115-141 (1995).

Camerlenghi, A., Cita, M. B., Hieke, W. & Ricchiuto, T.S., Geological evidence for mud diapirism on the Mediterranean Ridge accretionary complex. *Earth Planet. Sci. Lett.*, **109**, 493-504 (1992).

Camerlenghi, A. & Pini, G. A, Mud volcanoes, olistostromes and Argille scagliose in the Mediterranean region. *Sedimentology*, **56**, 319-365 (2009).

Casas, D. G., et al. Physical properties and their relationship to sedimentary processes and texture in sediments from mud volcanoes in the Anaximander Mountains (Eastern Mediterranean), *Scient. Mar.* **70**, 643-659 (2006).

Chamot-Rooke, N., Rabaute, A. & Kreemer, C. Western Mediterranean Ridge mud belt correlates with active shear strain at the prism-backstop geological contact: *Geology* **33**, 861–864 (2005).

Charlou, J. L., et al. Evidence of methane venting and geochemistry of brines on mud volcanoes of the eastern Mediterranean Sea. *Deep Sea Res., Part I*, **50**, 941-958 (2003) doi:10.1016/S0967-0637(03)00093-1.

Cita, M. B., et al. Discovery of mud diapirism in the Mediterranean Ridge: a preliminary report. *Boll. Soc. Geol. Ital.* **108**, 537-543 (1989).

Cita, M. B., et al. Fluid venting, mud volcanoes and mud diapirs on the Mediterranean Ridge. *Rend. Fis. Acc. Lin. Roma* **5**, 161-169 (1994).

Cita, M.B., et al. Stratigraphy and sedimentation in the Mediterranean Ridge diapiric belt. *Mar. Geol.* **132**, 131-150 (1996a).

Cita, M. B., Ivanov, M. K. & Woodside, J. (Eds.) The Mediterranean Ridge diapiric belt: introduction. *Mar. Geol.* **132**, 1-6 (1996b).

Cita, M. B. & Ryan, W. F. B. l. Paggi, Prometheus mud breccia: an example of shale diapirism in the western Mediterranean ridge. *Ann. Géolog. des Pays Hell.* **3**, 543-570 (1981).

Dählmann, A. & De Lange, G. J. Fluid-sediment interactions at Eastern Mediterranean mud volcanoes: a stable isotope study from ODP Leg 160. *Earth Planet. Sci. Lett.* **212**, 377-391 (2003).

Huguen, C. J., et al. Structural setting and tectonic control of mud volcanoes from the Central Mediterranean Ridge (Eastern Mediterranean). *Mar. Geol.* **209**, 245-263 (2004).

Ivanov, M. K., Limonov, A. F., & van Weering, T. C. E. Comparative characteristics of the Black Sea and Mediterranean Ridge mud volcanoes. *Mar. Geol.* **132**, 253-271 (1996).

Kopf, A. J., Clennell, B. & Brown, K.M. Physical properties of muds extruded from mud volcanoes: Implications for episodicity of eruptions and relationship to seismicity in *Mud Volcanoes, Geodynamics and Seismicity* (eds. Martinelli, G. & Panahi, B.) 263-283 (Springer, 2005).

Kopf, A., Mascle, J. & Klaeschen, D. The Mediterranean Ridge: a mass balance across the fastest growing accretionary complex on Earth. *J. Geophys. Res.* **108**, 2372 (2003) doi:10.1029/2001JB000473.

Kopf, A., Robertson, A. H. F. & Volkmann, N. Origin of mud breccia from the Mediterranean Ridge accretionary complex based on evidence of maturity of organic matter and related petrographic and regional tectonic evidence. *Mar. Geol.* **166**, 65-82 (2000).

Kioka, A. & Ashi, J. Episodic massive mud eruptions from submarine mud volcanoes examined through topographical signatures. *Geophys. Res. Lett.* **42,** 8406-8414 (2015).

Kioka, A., et al. Possible mechanism of mud volcanism at the prism-backstop contact in the western mediterranean ridge accretionary complex. *Mar. Geol.* **363**, 52-64 (2015).

Kioka, A., Tsuji, T., Otsuka, H. & Ashi, J. Methane concentration in mud conduits of submarine mud volcanoes: A coupled geochemical and geophysical approach. *Geochem., Geophys., Geosys.* **20**, 792-813 (2019).

Lykousis, V., et al. Mud volcanoes and gas hydrates in the Anaximander mountains (Eastern Mediterranean Sea). *Mar. Petr. Geol.* **26**, 854-872 (2009).

Perissoratis, C., et al. Thessaloniki mud volcano, the shallowest gas hydrate-bearing mud volcano in the Anaximander mountains, Eastern Mediterranean. *J. Geol. Res.* **2011**, Article ID 247983 (2011).

Perissoratis, C., Papadopoulos, G. & Zimianitis, E. Occurrence of mud diapirism in the marine sector between Kos and Kalimnos islands, SE Aegean: preliminary results (in Greek). *Bull. Geol. Soc. Greece* **32**, 217-222 (1998).

Robertson, A. & Ocean Drilling Program Leg 160 Scientific Party. Mud volcanism on the Mediterranean Ridge: initial results of Ocean Drilling Program Leg 160. *Geology*, **24**, 239-242 (1996).

Robertson, A. H. F. & Kopf, A. Origin of clasts and matrix within Milano and Napoli mud volcanoes, Mediterranean Ridge accretionary complex, in *Proc. ODP (Ocean Drilling Program) College Station, TX* (eds. Robertson, A.H.F., et al.) 575-596 (*Sci. Results* **160**, 1998a).

Robertson, A. H. F. & Kopf, A. Tectonic setting and processes of mud volcanism on the Mediterranean Ridge Accretionary Complex: evidence from Leg 160, in *Proc. ODP (Ocean Drilling Program) College Station, TX* (eds. Robertson, A.H.F., et al.) 665-680 (*Sci. Results* **160**, 1998a).

Shnyukov, Y. F., Aliyev, A. A. & Rahmanov, R. R. Mud volcanism of Mediterranean, Black and Caspian seas: specificity of development and manifestations (in Russian). *Geol. Min. Res. World Ocean* **13**, 5-25 (2017).

Staffini, F., Spezzaferri, S., & Aghib, F. Mud diapirs of the Mediterranean Ridge: sedimentological and micropaleontological study of mud breccia. *Riv. Ital. Paleontol. Stratigr.* **99**, 225-254 (1993).

Zitter, T. A. C. *Mud Volcanism and Fluid Emissions in Eastern Mediterranean Neotectonic Zones* (Vrije University, 2004).

Zitter, T. A. C., Huguen, C., ten Veen, J. & Woodside, J. M. Tectonic control on mud volcanoes and fluid seeps in the Anaximander Mountains, eastern Mediterranean Sea, in *Postcollisional Tectonics and Magmatism in the Mediterranean Region and Asia* (eds. Dilek, Y. & Pavlides, S.) 615–631 (*GSA Spec. Paper* **409**, 2006).

Zitter T. A .C., Huguen C. & Woodside J. M. Geology of mud volcanoes in the eastern Mediterranean from combined sidescan sonar and submersible surveys. *Deep-Sea Res. I*, **52**, 457-475 (2005).

Zitter, T. A. C., Van Der Gaast, S. J. & Woodside, J. M.. New information concerning clay mineral provenance in mud volcanoes, in *Proc. 36th CIESM Congress, Monaco,* 46-47 (*Rapp. Comm. Inter. Mer Médit*. **36**, 2001).

## 9. Levant Basin (Including Latakia and Cyprus Ridges)

Bertoni, C., et al. Seismic indicators of focused fluid flow and cross-evaporitic seepage in the Eastern Mediterranean. *Mar. Petr. Geol.* **88**, 472-488 (2017).

Hübscher, C., et al. Salt tectonics and mud volcanism in the Latakia and Cyprus Basins, eastern Mediterranean. *Tectonophysics*, **470**, 173-182 (2009).

Huguen, C., et al. Menes caldera, a highly active site of brine seepage in the Eastern Mediterranean Sea: “in situ” observations from the NAUTINIL expedition (2003). *Mar. Geol.* **261**, 138-152 (2009).

Kirkham, C., Cartwright, J., Hermanrud, C. & Jebsen, C. The genesis of mud volcano conduits through thick evaporite sequences. *Bas. Res.* **30**, 217-236 (2018).

Mascle, J., et al. Distribution and geological control of mud volcanoes and other fluid/free gas seepage features in the Mediterranean Sea and nearby Gulf of Cadiz. *Geo-Mar. Lett.* **34**, 89-110 (2014).

Mascle, J., et al. Marine geological evidence for a Levantine–Sinai plate, a missing piece of the Mediterranean puzzle. *Geology* **28**, 779-782 (2000).

## 10. Nile Delta

Aal, A.A., et al. Tectonic evolution of the Eastern Mediterranean Basin and its significance for the hydrocarbon prospectivity of the Nile Delta deepwater area. *GeoArabia* **6**, 363-384 (2001).

Bellaiche, G., et al. Le cône sous-marin du Nil et son réseau de chenaux profonds: Nouveaux résultats (campagne Fanil). *CRAS, Paris* **333**, 399-404 (2001).

Dolson, J .C., Boucher, P. J., Siok, J. & Heppard, P. D. Key challenges to realizing full potential in an emerging giant gas province: Nile Delta/Mediterranean offshore, deep water, Egypt, in *Petroleum Geology: North-West Europe and Global Perspectives* (eds. Doré, A. G. & Vining, B. A.), 607-624 (*Proc. 6th Petroleum Geology Conference Series* **6**, 2005).

Dupré S., et al. Seafloor geological studies above active gas chimneys off Egypt (Central Nile Deep Sea Fan). *Deep-Sea Res., A, Oceanogr. Res. Pap.* **54**, 1146-1172 (2007).

Dupré, S., et al. Warm brine lakes in craters of active mud volcanoes, Menes caldera off NW Egypt: evidence for deep-rooted thermogenic processes. *Geo-Mar. Lett.* **34**, 153-168 (2014).

Dupré, S., et al. Widespread active seepage activity on the Nile Deep Sea Fan (offshore Egypt) revealed by high-definition geophysical imagery. *Mar. Geol.* **275**, 1-19 (2010).

Feseker, T., et al. Active mud volcanoes on the upper slope of the western Nile deep-sea fan—first results from the P362/2 cruise of R/V Poseidon. *Geo-Mar. Lett.* **30**, 169-186 (2010).

Giresse, P., et al. Nature and origin of sedimentary clasts associated with mud volcanoes in the Nile deep-sea fan: relationships with fluid venting. *Sed. Geol.* **228**, 229–245 (2010).

Loncke L., et al. The Nile deep-sea fan: an example of interacting sedimentation, salt tectonics, and inherited subsalt paleotopographic features. *Mar. Petr. Geol.* **23**, 297-315 (2006).

Loncke, L., Mascle, J. & Fanil Scientific Parties. Mud volcanoes, gas chimneys, pockmarks and mounds in the Nile deep-sea fan (Eastern Mediterranean): geophysical evidences. *Mar. Petr. Geol.* **21**, 669-689 (2004).

Mascle, J., et al. Distribution and geological control of mud volcanoes and other fluid/free gas seepage features in the Mediterranean Sea and nearby Gulf of Cadiz. *Geo-Mar. Lett*. **34**, 89-110 (2014)

Mascle, J., et al. Marine geological evidence for a Levantine–Sinai plate, a missing piece of the Mediterranean puzzle. *Geology*, **28**, 779-782 (2000).

Mastalerz V., de Lange, G. J., Dählmann, A. & Feseker, T. Active venting at the Isis mud volcano, offshore Egypt: origin and migration of hydrocarbons. *Chem. Geol.* **246**, 87-106 (2007).

Pierre, C., et al. Authigenic carbonates related to active seepage of methane-rich hot brines at the Cheops mud volcano, Menes caldera (Nile deep-sea fan, eastern Mediterranean Sea). *Geo-Mar. Lett.* **34**, 253-267 (2014).

Prinzhofer, A. & Deville, E. Origins of hydrocarbon gas seeping out from offshore mud volcanoes in the Nile delta. *Tectonophysics* **591**, 52-61 (2013).

Tari, G., et al. Play types of the deep-water Matruh and Herodotus basins, NW Egypt. *Petr. Geosci.* **18**, 443-455 (2012).

Vandré, C., Cramer, B., Gerling, P., & Winsemann, J. Natural gas formation in the western Nile delta (Eastern Mediterranean): thermogenic versus microbial. *Org. Geochem.* **38**, 523-539 (2007).

## 11. Black Sea

Afanasenkov, A. P., Nikishin, A. M. & Obukhov, A. N. *Geology of the Eastern Black Sea* [in Russian with English summary] (Scientific World, Moscow, 2007).

Bohrmann, G., et al. Mud volcanoes and gas hydrates in the Black Sea: new data from Dvurechenskii and Odessa mud volcanoes. *Geo-Mar Lett.* **23**, 239-249 (2003).

Derman, A. S. Petroleum systems of Turkish basins. *AAPG Mem.* **106**, 469-504 (2014).

Derman, A. S. & Iztan, Y. H. Results of geochemical analysis of seeps and potential source rocks from Northern Turkey and the Turkish Black Sea, in *Regional and Petroleum Geology of the Black Sea and Surrounding Region* (ed. Robinson, A.G.) 313-330 (*AAPG Mem.* **68**, 1997).

Dimitrov, L., Contribution to atmospheric methane by natural seepages on the Bulgarian continental shelf. *Cont. Shelf Res.* **22**, 2429-2442 (2002a).

Dimitrov, L. I. Mud volcanoes—the most important pathway for degassing deeply buried sediments. *Earth Sci. Rev.* **59**, 49-76 (2002b).

Ivanov, M. K., Konyukhov, A. U., Kulnitskii, L. M., & Musatov, A. A., Mud volcanoes in deep part of the Black Sea (in Russian). *Vestnik MGU Ser. Geol.* **3**, 21-31 (1989).

Ivanov, M. K., Limonov, A. F., & Cronin, B. Mud volcanism and fluid venting in the eastern part of the Mediterranean Ridge. *Mar. Sci.* **68**, 126 (UNESCO Rep., 1996a).

Ivanov, M. K., Limonov, A. F., & van Weering, T. C. E. Comparative characteristics of the Black Sea and Mediterranean Ridge mud volcanoes. *Mar. Geol.* **132**, 253-271 (1996b).

Khriachtchevskaia, O., Stovba, S. & Popadyuk, I. Hydrocarbon prospects in the Western Black Sea of Ukraine. *Lead. Edge* **28**, 1024-1029 (2009).

Körber, J.-H., et al. Natural oil seepage at Kobuleti Ridge, eastern Black Sea. *Mar. Petr. Geol.* **50** 68-82 (2014).

Krastel, S., et al. Acoustic investigations of mud volcanoes in the Sorokin Trough, Black Sea. *Geo-Mar. Lett.* **23**, 230-238 (2003).

Kruglyakova, R., Gubanov, Y., Kruglyakov, V. &Prokoptsev, G. Assessment of technogenic and natural hydrocarbon supply into the Black Sea and seabed sediments. *Cont. Shelf Res.* **22**, 2395-2407 (2002).

Naudts, L., et al. Geological and morphological setting of 2778 methane seeps in the Dnepr paleo-delta, northwestern Black Sea. *Mar. Geol.* **227**, 177-199 (2006).

Palabiyik, Y., Ozdemir, A. & Karataş, A. The potential targets and drilling locations suggested for hydrocarbon discovery of Turkey in the Black Sea basin. (*International Black Sea Coastline Countries Scientific Research Symposium-IV, Giresun, Turkey*, 2020).

Popov, S. V., et al. Late Miocene to Pliocene palaeogeography of the Paratethys and its relation to the Mediterranean. *Palaeogeogr. Palaeoclimatol. Palaeoecol*. **238**, 91–106 (2006) doi:10.1016/j.palaeo.2006.03.020.

Römer, M., Sahling, H., dos Santos Ferreira, C. & Bohrmann, G. Methane gas emissions of the Black Sea—mapping from the Crimean continental margin to the Kerch Peninsula slope. *Geo-Mar. Lett*. **40**, 467–480 (2019).

Sahling, H., et al. Vodyanitskii mud volcano, Sorokin trough, Black Sea: geological characterization and quantification of gas bubble streams. *Mar. Petr. Geol.* **26**, 1799-1811 (2009).

Shnyukov, E. & Yanko-Hombach, V. *Mud Volcanoes of the Black Sea Region and Their Tnvironmental Significance* (Springer, 2020).

Sipahioglu, N.O., Karahanoglu, N. & Altiner, D. Analysis of Plio-Quaternary deep marine systems and their evolution in a compressional tectonic regime, Eastern Black Sea Basin. *Mar. Petro. Geol.* **43**, 187-207 (2013).

Starostenko, V. I., et al. Methane in the northern Black Sea: characterization of its geomorphological and geological environments in *Sedimentary Basin Tectonics from the Black Sea and Caucasus to the Arabian Platform* (eds. Sosson, M., et al.) (*Geol. Soc., Spec. Pubs.* **340**, 57-75, 2010).

Stovba, S., Khriachtchevskaia, O. & Popadyuk, I. Hydrocarbon bearing areas in the eastern part of the Ukrainian Black Sea. *Lead. Edge* **28**, 1042-1045 (2009).

Tari, G. C. & M. D. Simmons. History of deepwater exploration in the Black Sea and an overview of deepwater petroleum play types, in *Petroleum Geology of the Black Sea* (eds. Simmons, M. D., Tari, G. C. & Okay, A. I.) (*Geol. Soc., Spec. Pubs.* **464**, 439-475, 2018).

Wu, T., et al. Morphology and activity of the Helgoland Mud Volcano in the Sorokin Trough, northern Black Sea. *Mar. Petr. Geol.* **99**, 227-236 (2019).

Xing, J. & Spiess, V. Shallow gas transport and reservoirs in the vicinity of deeply rooted mud volcanoes in the central Black Sea. *Mar. Geol.* **369**, 67-78 (2015).

## 12. Sea of Azov and Crimea Peninsula

Fedorov, S. F. Mud Volcanoes of the Crimean–Caucasian Geological Province and Diapirism, Rezul'taty Issledovaniya Gryazevykh Vulkanov Krymsko-Kavkazskoi Geologicheskoi Provintsii (Results of Studies of Mud Volcanoes in the Crimean–Caucasian Geological Province) (Moscow: USSR Academy of Sciences, 1939).

Goubkin, I. M. & Fedorov, S. F., Грязевые вулканы Советского Союза и их связь с генезисом нефтяных месторождений в Крымско-Кавказской геологической провинции (Mud Volcanoes of the Soviet Union and Their Connection with the Genesis of Petroleum Fields in Crimean-Caucasus Geologic Province) (USSR Academy of Science, Moscow, 1938).

Herbin J. P., et al. Oil seeps from the "Boulganack" mud volcano in the Kerch Peninsula (Ukraine-Crimea), in Study of the Mud and the Gas: Inferences for the Petroleum Potential (Oil & Gas Sci. & Tech.—Rev. IFP **63**, 609-628 (2008).

Popkov, V. I., Fold–thrust dislocations in sedimentary cover of the Sea of Azov. Geotectonics **43**, 324-332 (2009).

Popkov, V. I. Imbricate thrust structure of the northwestern caucasus. Doklady Earth Sci. **411**, 1222-1224 (2006).

Saintot, A. & Angelier, J. Plio-Quaternary paleostress regimes and relation to structural development in the Kertch-Taman peninsulas (Ukraine and Russia). J. Struc. Geol. **22**, 1049-1064 (2000).

Shniukov, E.F., et al. Грязевые вулканы Керченско-Таманского региона (The Mud-Volcanoes of the Kerch-Taman Region) (Glavmedia, Krasnodar, 2006).

## 13. South Caspian Sea (Azerbaijan, N Iran and Turkmenistan)

Aliyev, A. A. Recent eruptions of mud volcanoes in Azerbaijan (geologic-geochemical aspect), in *AAPG Annual Convention* (AAPG Salt Lake City, 2003).

Aliyev, A. A., Guliyev, I. S., & Belov, I. S. *Catalogue of Recorded Eruptions of Mud Volcanoes of Azerbaijan (for Period of Years 1810-2001)* (Nafta-Press, Baku 2002).

Antonielli, B., et al. Pre-eruptive ground deformation of Azerbaijan mud volcanoes detected through satellite radar interferometry (DInSAR). *Tectonophysics* **637**, 163-177 (2014).

Babayev, G., Tibaldi, A., Bonali, F. L. & Kadirov, F. Evaluation of earthquake-induced strain in promoting mud eruptions: the case of Shamakhi–Gobustan–Absheron areas, Azerbaijan. *Nat. Haz.* **72**, 789-808 (2014).

Berner, U., Scheeder, G., Kus, J. & Movsumova, U. Mud volcanoes of Azerbaijan—windows to the subsurface. (*Search & Disc.* **40469**, 2009).

Bolourchi, M. J. Preliminary Report, *East Caspian Sea Mud Volcano and Review to Turkman Region Mud Volcanoes (Golestan Province)* (Geol. Surv. Iran, 2002).

Bredehoeft, J. D., Djevanshir, R. D. & Belitz, K. R. Lateral fluid flow in a compacting sand-shale sequence: South Caspian basin. *AAPG Bull.* **72**, 416-424 (1988).

Davies, R. J. & Stewart S. A. Emplacement of giant mud volcanoes in the South Caspian Basin: 3D seismic reflection imaging of their root zones. *J. Geol. Soc.* **162**, 1-4 (2005).

Dimitrov, L. I. Mud volcanoes—the most important pathway for degassing deeply buried sediments. *Earth-Sci. Rev.* **59**, 49-76 (2002).

Evans, R. J., Davies, R. J. & Stewart, S. A. Internal structure and eruptive history of a kilometre-scale mud volcano system, South Caspian Sea. *Bas. Res.* **19**, 153-163 (2006).

Evans, R. J., Stewart, S. A. & Davies, R. J. Phase-reversed seabed reflections in seismic data: examples related to mud volcanoes from the South Caspian Sea. *Geo-Mar. Lett.* **27**, 203-212 (2007).

Evans, R. J., Stewart, S. A. & Davies, R. J. The structure and formation of mud volcano summit calderas. *J. Geol. Soc*. **165**, 769-780 (2008).

Feyzullayev, A. A. Mud volcanoes in the South Caspian basin: nature and estimated depth of its products. *Nat. Sci.* **4**, 445-453 (2012).

Fowler, S. R., et al. Mud volcanoes and structural development on Shah Deniz. *J. Petr. Sci. Eng.* **28**, 189-206 (2000).

Gherasim, M., et al. Application of an integrated workflow for shallow-hazard characterization using a 3D high-resolution survey offshore Azerbaijan. *Lead. Edge* **April**, 390-396 (2015).

Guliev, I. & Panahi, B. Geodynamics of the deep sedimentary basin of the Caspian Sea region: paragenetic correlation of seismicity and mud volcanism. *Geo-Mar. Lett.* **24**, 169-176 (2004).

Haroon, A., et al. Joint inversion of long-offset and central-loop transient electromagnetic data: Application to a mud volcano exploration in Perekishkul, Azerbaijan. *Geophys. Prosp.* **63**, 478-494 (2015).

Hovland, M., Hill, A. & Stokes D. The structure and geomorphology of the Dashgil mud volcano. *Azerbaijan Geomorph.* **21**, 1-15 (1997).

Huseynov, D. A. & Guliyev I. S. Mud volcanic natural phenomena in the South Caspian Basin: geology, fluid dynamics and environmental impact. *Env. Geol.* **46**, 1012-1023 (2004).

Isaksen, G. H., et al. Regional evaluation of source rock quality in Azerbaijan from the geochemistry of organic-rich rocks in mud-volcano ejecta, in *Oil and Gas of the Greater Caspian Sea* (eds. Yilmaz, P. O. & Isaksen, G. H.) 51-64 (AAPG, Tulsa, 2007).

Khain, V. E., Gadjiev, A. N. & Kengerli, T. N. Tectonic origin of the Apsheron Threshold in the Caspian Sea. *Doklady Earth Sci.* **414**, 552-556 (2007).

Kopf, A., et al. In situ cone penetration tests at the active Dashgil mud volcano, Azerbaijan: evidence for excess fluid pressure, updoming, and possible future violent eruption. *Mar. Petr. Geol.* **26**, 1716-1723 (2009).

Narimanov, A. A. The petroleum systems of the South Caspian Basin, in *Basin Modelling: Advances and Applications*, (eds. Doré, A. G., et al.) (*Norw. Petr. Soc. Spec. Pubs. Els.*, **3**, 599-608 (1993).

Omrani, H. & Raghimi, M. Origin of the mud volcanoes in the South East Caspian Basin, Iran. *Mar. Petr. Geol.* **96**, 615–626 (2018).

Oppo, D. & Capozzi, R. Spatial association of mud volcano and sandstone intrusions, Boyadag anticline, western Turkmenistan. *Bas. Res.* **28**, 827-839 (2016).

Oppo, D., Capozzi, R., Nigarov, A. & Esenov, P. Mud volcanism and fluid geochemistry in the Cheleken peninsula, western Turkmenistan. *Mar. Petr. Geol.*, **57**, 122-134 (2014).

Planke, S., et al. Mud and fluid migration in active mud volcanoes in Azerbaijan. *Geo-Mar. Lett.* **23**, 258-268 (2003).

Ranjbaran, M. & Sotohian, F. Environmental impact and sedimentary structures of mud volcanoes in southeast of the Caspian Sea basin, Golestan Province, Iran. *Caspian J. Env. Sci.* **13**, 391-405 (2015).

Roberts, K. S., Davies, R. J. & Stewart, S. A. Structure of exhumed mud volcano feeder complexes, Azerbaijan. *Bas. Res.* **22**, 439-451 (2010).

Sánchez-Borrego, I. R., Soto, J. I., Rueda, M. & Santos Betancor, I. Nonparametric estimation to reconstruct the deformation history of an active fold in the Caspian Basin. *Math. Geosci.* **48**, 985-1011 (2016).

Santos Betancor, I. & Soto, J. I. 3D geometry of a shale-cored anticline in the western South Caspian Basin (offshore Azerbaijan). *Mar. Petr. Geol.* **67**, 829-851 (2015).

Yakubov, A. A., Alizade, A. A. & Zeinalov, M. M. *Mud Volcanoes of Azerbajan SSR (Atlas)* (Elm, Baku, in Russian, 1971).

Yusifov, M. *Seismic Interpretation and Classification of Mud Volcanoes of the South Caspian Basin, Offshore Azerbaijan* (Texas A&M University, 2004).

Yusifov, M. & Rabinowitz, P.D. Classification of mud volcanoes in the South Caspian Basin, offshore Azerbaijan. *Mar. Petr. Geol.* **21**, 965-975 (2004).

## 14. Burgan Arch (Kuwait)

Duane, M. J., Reinink-Smith, L., Eastoe, C. & Al-Mishwat, A .T. Mud volcanoes and evaporite seismites in a tidal flat of northern Kuwait—implications for fluid flow in sabkhas of the Persian (Arabian) Gulf. *Geo-Mar. Lett.* **35**, 237-246 (2015).

## 15. Makran Accretionary Wedge (Onshore and Offshore Iran and Pakistan)

Ahmed, S. S. Tertiary geology of part of South Makran, Baluchistan, West Pakistan. *AAPG Bull.* **53**, 1480-1499 (1969).

Babadi, M.F., et al. Origin of fluids discharged from mud volcanoes in SE Iran. *Mar. Petr. Geol.* **106**, 190-205 (2019).

Bannert, D., Cheema, A., Ahmed, A. & Schäffer, U. The structural development of the Western Fold Belt. *Pak. Geol. Jahrb.* **B80**, 3-60 (1992).

Calvès, G., et al. Cenozoic mud volcano activity along the Indus Fan: offshore Pakistan. *Bas. Res.* **22**, 398-413 (2010).

Christie, W.A.K. Gas from a mud volcano in Mekran. *Records Geol. Surv. India*, **42**, 279-280 (1912).

Collier, J. S. & White, R. S. Mud diapirism within Indus Fan sediments: Murray Ridge, Gulf of Oman. *Geophys. J. Int.*, **101**, 345-353 (1990).

Ding, F., et al. Interaction between accretionary thrust faulting and slope sedimentation at the frontal Makran accretionary prism and its implications for hydrocarbon fluid seepage. *J. Geophys. Res.* **115**, (2010).

Delisle, G., Mud Volcanoes of Pakistan — an overview, in *Mud Volcanoes, Geodynamics and Seismicity*. (eds. Martinelli, G. & Panahi, B.) (*NATO Sci. Series IV: Earth and Env. Series* **51**, 159-169, Springer, Dordrecht, 2005).

Delisle, G. The mud volcanoes of Pakistan. *Env. Geol.*, **46**, 1024-1029 (2004) doi:10.1007/s00254-004-1089-x.

Delisle, G., et al. Active mud volcanoes on- and offshore eastern Makran, Pakistan. *Int. J. Earth Sci.* **91**, 93-110 (2002).

Ding, F., et al. Interaction between accretionary thrust faulting and slope sedimentation at the frontal Makran accretionary prism and its implications for hydrocarbon fluid seepage. *J. Geophys. Res. Sol. Earth* **115**, (2010).

Ellouz-Zimmermann, N., et al. Impact of sedimentation on convergent margin tectonics: example of the Makran Accretionary Prism (Pakistan), in *Thrust Belts and Foreland Basins: from Fold Kinematics to Hydrocarbon Systems* (eds. Lacombe, O., Roure, F., Lavé, J. & Vergés, J.) 327-350 (Springer, 2007a).

Ellouz-Zimmermann, N., et al. Offshore frontal part of the Makran Accretionary Prism: the Chamak survey (Pakistan), in *Thrust Belts and Foreland Basins: from Fold Kinematics to Hydrocarbon Systems* (eds. Lacombe, O., Roure, F., Lavé, J. & Vergés, J.) 351-366 (Springer, 2007b).

Fowler, S. R., White, R. S. & Louden, K. E. Sediment dewatering in the Makran accretionary prism. *Earth Planet. Sci. Lett.,* **75**, 427-438 (1985).

Grando, G. & McClay, K. Morphotectonics domains and structural styles in the Makran accretionary prism, offshore Iran. *Sed. Geol.* **196**, 157-179 (2007).

Harms, J. C., Cappel, H. N. & Francis, D. C. The Makran coast of Pakistan: its stratigraphy and hydrocarbon potential, in *Marine Geology and Oceanography of Arabian Sea and Coastal Pakistan*. (eds. Haq, B. U. & Milliman, J. D.) 3-26 (Van Nostrand Reinhold, New York, 1984).

Harrison, J. V. Coastal Makran. *Geograph. J.* **97**, 1-15 (1941).

Harrison, J. V. Mud volcanoes on the Makran coast. *Geograph. J.,* **103**, 180-181 (1944).

Hart, S. V .W. Some account of a journey from Kurrachee to Hinglaj, in the Lus Territory, descriptive of the Intermediate Country, and of the Port of Soumeanee. *J. Asiatic Soc. Bengal,* **9**, 134-154 (1840).

Hosseini-Barzai, M. & Talbot, C. J. A tectonic pulse in the Makran accretionary prism recorded in Iranian coastal sediments. *J. Geol. Soc. Lond.* **160**, 903-910 (2003).

Kukowski, N., et al. Morphotectonics and mechanics of the central Makran accretionary wedge of Pakistan. *Mar. Geol.* **173**, 1-19 (2001).

Platt, J. P., Leggett, J. K. & Alam, S. Slip vectors and fault mechanics in the Makran accretionary wedge, southwest Pakistan. *J. Geophys. Res*., **93**, 7955-7973 (1988).

Römer, M., et al. Quantification of gas bubble emissions from submarine hydrocarbon seeps at the Makran continental margin (offshore Pakistan). *J. Geophys. Res. Oceans,* **117** (2012).

Schleder, Z., et al. Structural style in a Messinian (intra-Pontian) gravity-driven deformation system, western Black Sea, offshore Romania. *Petr. Geosci.,* **22**, 400-410 (2016).

Schlüter, H. U., et al. The Makran accretionary wedge: sediment thickness and ages and the origin of mud volcanoes. *Mar. Geol.* **185**, 219-232 (2002).

Skrine, C. P. The Quetta earthquake. *Geogr. J.,* **88**, 414-430 (1936).

Snead, R. J. Active mud volcanoes of Baluchistan, West Pakistan. *Geograph. Rev.* **54**, 545-560 (1964).

Stiffe, A. W. On the mud-craters and geological structure of the Mekran coast. *Quart. J. Geol. Soc. London* **30**, 50-53 (1874).

von Rad, U., et al. Gas and fluid venting at the Makran accretionary wedge of Pakistan: initial results. *GeoMar. Lett.* **20**, 10-19 (2000).

Vredenbur, E. W. Report on the Geology of Sarawan, Jhalawan, Mekran and the State of Las Bela, considered principally from the point of view of economic development. *Rec. Geol. Surv. India,* **38**, 189-215 (1909–1910).

Wiedicke, M., Neben, S. & Spiess, V. Mud volcanoes at the front of the accretionary complex, Pakistan. *Mar. Geol.* **172**, 57-73 (2001).

## 16. Ganges-Brahmaputra Delta (Offshore Bangladesh)

Akhter, S. H. *Structure, Stratigraphy and Sedimentology of the Upper Tertiary Sediments of the Central Part of the Sitakund Hill Range, Chittagong, Bangladesh*. (Dhaka University, Bangladesh, 1979).

Zahid, K. M. & Uddin, A. Influence of overpressure on formation velocity evaluation of Neogene strata from the eastern Bengal Basin, Bangladesh. *J. Asian Earth Sci.* **25**, 419-429 (2005).

## 17. Burma Arc

Steckler, M. S, Akhter, S. H. & Seeber, L. (2008). Collision of the Ganges-Brahmaputra Delta with the Burma Arc: implications for earthquake hazard. *Earth Planet. Sci. Lett.* **273**, 367-378.

## 18. Andaman Basin (Offshore Myanmar and Thailand)

He, W., et al. Study on tectonic and evolution characteristics of basins in Andaman Sea. *Fault-Block & Gas Field* **18**, 178–82 (2011).

He, W. & Zhou, J. Structural features and formation conditions of mud diapirs in the Andaman Sea Basin. *Geol. Mag.* **156**, 659-668 (2019).

Morley, C. K. Evolution from an oblique subduction back-arc mobile belt to a highly oblique collisional margin: the Cenozoic tectonic development of Thailand and eastern Myanmar, in *Earth Accretionary Systems in Space and Time* (eds. Cawood, P. A. & Kröner, A.) (*Geol. Soc. Spec. Pubs.* **318**, 373-403, 2009).

Nielsen, C., Chamot-Rooke, N. & Rangin, C. From partial to full strain partitioning along the Indo-Burmese hyper-oblique subduction. *Mar. Geol.* **209**, 303-27 (2004).

Srisuriyon, K. & Morley, C. K. Pull-apart development at overlapping fault tips: Oblique rifting of a Cenozoic continental margin, northern Mergui Basin, Andaman Sea. *Geosphere* **10**, 80-106 (2014).

## 19. Javu-Lamu Basin (Offshore S Somalia-N Kenia)

Coffin, M. F. & Rabinowitz, P. D. East African continental margin transect, in *Seismic Expression of Structural Styles: A Picture and Work Atlas* (ed. Bally, A. W.) 22-30 (*Stud. Geol*. 15, **2**, 1983).

Coffin, M. F. & Rabinowitz, P. D. Evolution of the conjugate East African-Madagascan margins and the western Somali Basin. *Geol. Soc. Am. Spec. Pap.* **226**, 1-79 (1988).

Coffin, M. F. & Rabinowitz, P. D. Reconstruction of Madagascar and Africa: evidence from the Davie Fracture Zone and the Western Somali Basin. *J. Geophys. Res.* **92**, 9385-9406 (1987).

Coffin, M. F. & Rabinowitz, P. D. The Mesozoic East African and Madagascan conjugate margins: stratigraphy and tectonics, in *Geology and Geophysics of Continental Margins*. (ed. Watkins, J. S. et al.) 207-246 (*AAPG Mem.* **53**, 1992).

Coffin, M. F., Rabinowitz, P. D. & Houtz, R. E. Crustal structure in the Western Somali Basin. *Geophys. J. R. Astron. Soc.* **86**, 331-369 (1986).

Cruciani, F. & Barchi, M. R. The Lamu Basin deepwater fold-and-thrust belt: An example of a margin-scale, gravity-driven thrust belt along the continental passive margin of East Africa. *Tectonics* **35**, 491-510 (2016).

Kearns, H., Berryman, J., Hodgson, N. & Rodriguez, K. Offshore Somalia: East Africa's oil frontier. *GeoExPro* **13** (2016).

Rabinowitz, P. D., Coffin, M. F. & Falvey, D. A. Salt diapirs bordering the continental margin of northern Kenya and southern Somalia. *Science* **215**, 663-665 (1982).

## 20. Rovuma Delta (Offshore N Mozambique-S Tanzania)

Fletcher, T. The Windjammer discovery: Play opener for offshore Mozambique and East Africa. *Giant Fields of the Decade 2000–2010* (eds. Merrill, R. K. & Sternbach, C. A.) 273-304 (*AAPG Mem.* **113**, 2017).

Mahanjane, E. S. & Franke, D. The Rovuma Delta deep-water fold-and-thrust belts, offshore Mozambique. *Tectonophysics* **614**, 91-99 (2014).

Salman, G. & Abdula, I. Development of the Mozambique and Ruvuma sedimentary basins, offshore Mozambique. *Sediment. Geol.* **96**, 7-41 (1991).

## 21. Banda Arc Orogen (Java to Sumba and Timor)

Barber, A. J., Tjokrosapoetro, S. & Charlton T. R. Mud volcanoes, shale diapirs, wrench fault and melanges in accretionary complexes, eastern Indonesia. *AAPG Bull.* **70**, 1729-1741 (1986).

Breen, N. A., Silver, E. A. & Hussong, D. M. Structural styles of an accretionary wedge south of the island of Sumba, Indonesia, revealed by SeaMARC II side scan sonar. *GSA Bull.* **97**, 1250-1261 (1986).

Davies, R. J., Swarbrick, R. E., Evans, R. J. & Huuse, M. Birth of a mud volcano: East Java, 29 May 2006. *GSA Today* **17** (2007) doi:10.1130/GSAT01702A.1.

Harris, R. A., Sawyer, R. K. & Audley-Charles, M. G. Collisional melange development: Geologic associations of active melange-forming processes with exhumed mélange facies in the western Banda orogen, Indonesia. *Tectonics* **17**, 458-479 (1998).

Heim, A., Lebende diapire in den südöstlichein Molukken. *Eclo. Geol. Helv.*, **35**, 225–233 (1942).

Mazzini, A., et al. Triggering and dynamic evolution of the LUSI mud volcano, Indonesia. *Earth Planet. Sci. Lett*. **261**, 375-388 (2007).

Satyana, A. H. Mud diapirs and mud volcanoes in depressions of Java to Madura: origins, natures, and implications to petroleum system (*Proc. Indones. Petr. Assoc., 32nd Ann. Conv. & Exhib.*, 2008).

Tingay, M. Initial pore pressures under the Lusi mud volcano. *Indonesia Interp.* **3**, SE33-SE49 (2015).

## 22. Baram Delta (NW Brunei and Sarawak)

Back, S., Jing, T. H., Thang, T. X. & Morley, C. K. Stratigraphic development of synkinematic deposits in a large growth-fault system, onshore Brunei Darussalam. *Geol. Soc. London J.* **162**, 243-258 (2005).

Gee, M. J. R., et al. The Brunei slide: a giant submarine landslide on the North West Borneo margin revealed by 3-D seismic data. *Mar. Geol.* **246**, 9-23 (2007).

Hesse, S., Back, S. & Franke, D. The deep-water fold-and-thrust belt offshore NW Borneo: gravity-driven versus basement-driven shortening. *GSA Bull.* **121**, 939-953 (2009).

King R.C., et al. Balancing deformation in NW Borneo: Quantifying plate-scale vs. gravitational tectonics in a delta and deepwater fold-thrust belt system. *J. Mar. Petr. Geol.* **27**, 238-246 (2010).

Laird, A. P. & Morley, C. K. Development of gas hydrates in a deep-water anticline based on attribute analysis from three-dimensional seismic data. *Geosphere* **7**, 1-20 (2011).

Morley, C. K. Development of crestal normal faults associated with deepwater fold growth. *J. Struc. Geol*. **29**, 1148-1163 (2007).

Morley, C. K. Growth of folds in a deep-water setting. *Geosphere* **5**, 59-89 (2009).

Morley, C. K. Mobile shale related deformation in large deltas developed on passive and active margins. *Subsurface Sediment Mobilization* (eds. Van Rensbergen et al.) 335-357 (*Geol. Soc. Spec. Pubs.* **216**, 2003).

Morley, C. K., Crevello, P. & Ahmad Z. H. Shale tectonics and deformation associated with active diapirism: the Jerudong Anticline, Brunei Darussalam. *J. Geol. Soc.* **155**, 475-490 (1998).

Morley C. K., et al. Characteristics of repeated, detached, Miocene–Pliocene tectonic inversion events, in a large delta province on an active margin, Brunei Darussalam, Borneo. *J. Struc. Geol.* **25**, 1147-1169 (2003).

Morley, C. K., et al. Comparison of modern fluid distribution, pressure and flow in sediments associated with anticlines growing in deepwater (Brunei) and continental environments (Iran). *Mar. Petr. Geol.* **51**, 210-229 (2014).

Morley, C. K. & Leong, L. C. Evolution of deep-water synkinematic sedimentation in a piggyback basin, determined from three-dimensional seismic reflection data. *Geosphere* **4**, 939-962 (2008).

Morley, C. K., Tingay, M., Hillis, R. & King, R. Relationship between structural style, overpressures, and modern stress, Baram Delta Province, northwest Borneo. *J. Geophys Res.* **113**, B09410 (2008).

Tingay, M. R. P., et al. Evidence for overpressure generation by kerogen-to-gas maturation in the northern Malay Basin. *AAPG Bull.* **97**, 639-672 (2013).

Tingay, M. R. P., et al. Origin of overpressure and pore pressure prediction in the Baram Delta Province, Brunei. *AAPG Bull.* **93**, 51-74 (2009a).

Tingay, M. R. P., et al. Pore pressure/stress coupling in Brunei Darussalam—implications for shale injection, in *Subsurface Sediment Mobilization* (eds. Van Rensbergen, P., et al.) 369-379 (*Geol. Soc. Spec. Publ.* **216**, 2003).

Tingay, M. R. P., et al. Present-day stress and neotectonics of Brunei: implications for petroleum exploration and production. *AAPG Bull.* **93**, 75-100 (2009b).

Tingay, M. R. P., et al. Present-day stress orientation in Brunei: a snapshot of "prograding tectonics" in a Tertiary delta. *J. Geol. Soc. London* **162**, 39-49 (2005).

Tingay, M. R. P., et al. Variation in vertical stress in the Baram Basin, Brunei: tectonic and geomechanical implications. *Mar. Petrol. Geol.* **20**, 1201-1212 (2003b).

Tingay, M. R. P., et al. "Vertically transferred" overpressures in Brunei: evidence for a new mechanism for the formation of high magnitude overpressures. *Geology* **35**, 1023-1026 (2007).

Totake, Y., Butler, R. W. H., Bond, C. E. & Aziz, A. Analyzing structural variations along strike in a deep-water thrust belt. *J. Struc. Geol.* **108**, 213-229 (2018).

Van Rensbergen, P., et al. Structural evolution of shale diapirs from reactive rise to mud volcanism: 3D seismic data the Baram delta, offshore Brunei Darussalam. *J. Geol. Soc.* **156**, 633-650 (1999).

Van Rensbergen, P. & Morley, C. K. Re-evaluation of mobile shale occurrences on seismic sections of the Champion and Baram deltas, offshore Brunei, in *Subsurface Sediment Mobilization* (eds. Van Rensbergen, P., et al.) 395-409 (*Geol. Soc. Spec. Publ.* **216**, 2003).

Van Rensbergen, P. & Morley, C. K. 3D Seismic study of a shale expulsion syncline at the base of the Champion delta, offshore Brunei and its implications for the early structural evolution of large delta systems. *Mar. Petr. Geol.* **17**, 861-872 (2000).

Warren, J. K., Cheung, A. & Cartwright, I. Organic geochemical, isotopic, and seismic indicators of fluid flow in pressurized growth anticlines and mud volcanoes in modern deep-water slope and rise sediments of offshore Brunei Darussalam: implications for hydrocarbon exploration in other mud- and salt-diapir provinces, in *Shale Tectonics* (ed. Wood, L.) 163-196 (*AAPG Mem.* **93**, 2010).

### 23. Mahakam Delta (SE Indonesia)

McClay, K., Dooley, T., Ferguson, A. & Poblet, J. Tectonic evolution of the Sanga Sanga Block, Mahakam Delta, Kalimantan, Indonesia. *AAPG Bull.* **84**, 765-786 (2000).

### 24. Zhongjiannan Basin (Offshore Vietnam)

Chen, J., et al. Morphologies, classification and genesis of pockmarks, mud volcanoes and associated fluid escape features in the northern Zhongjiannan Basin, South China Sea. *Deep Sea Res. Pt. II: Top. Stud. Ocean.* **122**, 106-117 (2015).

Sun, Q., et al. The morphologies and genesis of mega-pockmarks near the Xisha Uplift, South China Sea. *Mar. Pet. Geol.* **28**, 1146-1156 (2011).

Sun, Q., et al. Focused fluid flow systems of the Zhongjiannan Basin and Guangle Uplift, South China Sea. *Bas. Res.* **25**, 97-111 (2013).

### 25. Yinggehai-Hanoi Depression (South China Sea and Offshore Vietnam)

Hao, F., Li, S., Gong, Z., & Yang, J. Mechanism of diapirism and episodic fluid injections in the Yinggehai Basin. *Sci. in China Ser. D: Earth Sci.* **45**, 151-159 (2002).

He, J., et al. Origin and distribution of mud diapirs in the Yinggehai Basin and their relation to the migration and accumulation of natural gas. *Geol. in China* **33**, 1337-1344 (2006).

Jin, Y., et al. Risk analysis of natural hydraulic fracturing in an overpressured basin with mud diapirs: A case study from the Yinggehai Basin, South China sea. *J. Petr. Sci. Eng.* **196**, 107621 (2021).

Lei, C., et al. The structure and formation of diapirs in the Yinggehai–Song Hong Basin, South China Sea. *Mar. Petr. Geol.* **28**, 980-991 (2011).

Liu, R., et al. In situ stress analysis in the Yinggehai Basin, northwestern South China Sea: implication for the pore pressure-stress coupling process. *Mar. Petr. Geol.* **77**, 341-352 (2016).

Wang, Z. F. & Huang, B. J. Dongfang 1-1 gas field in the mud diapir belt of the Yinggehai Basin, South China Sea. *Mar. Petr. Geol.* **25**, 445-455 (2008).

Wang, Z. F., He, J. X. & Xie, X. N.. Heat flow action and its control on natural gas migration and accumulation in mud-fluid diapir areas in Yinggehai Basin. *Earth Sci.* **29**, 203-210 (2004).

Xie, X. N., Li, S. T., Dong, W. L. & Hu, Z. Evidence for episodic expulsion of hot fluids along faults near diapiric structures of the Yinggehai Basin, South China Sea. *Mar. Petr. Geol.* **18**, 715-728 (2001).

Xie, X., Li, S., He, H. & Liu, X. Seismic evidence for fluid migration pathways from an overpressured system in the South China Sea. *Geofluids* **3**, 245-253 (2003).

Zhu, M., Graham, S. & McHargue, T. The Red River fault zone in the Yinggehai Basin, south China sea. *Tectonophysics* **476**, 397-41 (2009).

### 26. Xisha Trough and Pearl River Mouth Basin (South China Sea, SE Hainan Island)

Lei, C. & Ren, J. Hyper-extended rift systems in the Xisha Trough, northwestern South China Sea: Implications for extreme crustal thinning ahead of a propagating ocean. *Mar. Petr. Geol.* **77**, 846-864 (2016).

Yu, X., et al. Depositional characteristics and accumulation model of gas hydrates in northern South China Sea. *Mar. Petr. Geol.* **56**, 74-86 (2014).

### 27. Manila Trench (SW Taiwan)

Chao, H .C., You, C.-F. & Sun, C.-H. Gases in Taiwan mud volcanoes: chemical composition, methane carbon isotopes, and gas fluxes. *Appl. Geochem.* **25**, 428-436 (2010).

Chen, S.-C., et al. Gas seepage, pockmarks and mud volcanoes in the near shore of SW Taiwan. *Mar. Geophys. Res.* **31**, 133-147 (2010).

Chen, S., et al. Distribution and characters of the mud diapirs and mud volcanoes off southwest Taiwan. *J. Asian Earth Sci.* **92**, 201-214 (2014).

Chiu, J.-K., Tseng, W.-H. & Liu, C.-S. Distribution of gassy sediments and mud volcanoes offshore southwestern Taiwan. *Terres. Atmos. Ocean. Sci.* **17**, 703–722 (2006).

Doo, W.-B., et al. Gravity anomalies of the active mud diapirs off southwest Taiwan. *Geophys. J. Int.* **203**, 2089–2098 (2015).

Gieskes, J . M., et al. Hydro-geochemistry of mud volcanoes in Taiwan. *Acta Geol. Taiwan.* **30**, 7988 (1992).

Lin, A. T., et al. Tectonic features of the incipient arc-continent collision zone of Taiwan: implications for seismicity. *Tectonophysics* **479**, 28-42 (2009).

Liu, C.-S., Huang, I.-L. & Teng, L.S. Structural features off southwestern Taiwan. *Mar. Geol.* **137**, 305-319 (1997).

Liu, C.-S., Deffontaines, B., Lu, C.-Y. & Lallemand, S. Deformation patterns of an accretionary wedge in the transition zone from subduction to collision offshore southwestern Taiwan. *Mar. Geophys. Res*. **25**, 123-137 (2004).

Liu, C.-C., et al. Geochemical characteristics of the fluids and muds from two southern Taiwan mud volcanoes: Implications for water–sediment interaction and groundwater arsenic enrichment. *Appl. Geochem*. **24**, 1793-1802 (2009).

Shih, T. T. A survey of the active mud volcanoes in Taiwan and a study of their types and character of the mud. *Petrol. Geol. Taiwan* **5**, 259-311 (1967).

Sun, S.-C. & Liu, C.-S. Mud diapir and submarine channel deposits in offshore Kaosiung-Hengchun, southwest Taiwan. *Petrol. Geol. Taiwan* **28**, 1-14 (1993).

Yang, T. F., et al. Composition and exhalation flux of gases from mud volcanoes in Taiwan. *Env. Geol.* **46**, 1003-1011 (2004).

You, C.-F., et al. Geochemistry of mud volcano fluids in the Taiwan accretionary prism. *Appl. Geochem*. **19**, 695-707 (2004).

### 28. Okinawa Trough (East China Sea)

Luan, X., Wang, K., Hyndman, R. & Willoughby, E. Bottom simulating reflector and gas seepage in Okinawa Trough: evidence of gas hydrate in an active back-arc basin. *J. China Univ. Geosci.* **19**, 152-161 (2008).

Ning, X., et al. Gas hydrate associated with mud diapirs in southern Okinawa Trough. *Mar. Petr. Geol.* **26**, 1413-1418 (2009).

Xing, J., Jiang, X. & Li, D. Seismic study of the mud diapir structures in the Okinawa Trough. *Geol. J*. **51**, 203-208 (2016).

Xu, C., et al. Methane seepage inferred from pore water geochemistry in shallow sediments in the western slope of the Mid-Okinawa Trough. *Mar. Petr. Geol.* **98**, 306-315 (2018).

Xu, N., et al. Gas hydrate associated with mud diapirs in southern Okinawa Trough. *Mar. Petrol. Geol.* **26**, 1413-1418 (2009).

### 29. Yangtze Delta (E China Sea)

Chen, Z. & Stanley, D. J. Yangtze delta, eastern China: 2. Late Quaternary subsidence and deformation. *Mar. Geol.* **112**, 13–21 (1993).

Chen, Z., Song, B., Wang, Z. & Cai, Y. Late Quaternary evolution of the sub-aqueous Yangtze Delta, China: sedimentation, stratigraphy, palynology, and deformation. *Mar. Geol.* **162**, 423-441 (2002).

Luo, M., et al. Pockmark activity inferred from pore water geochemistry in shallow sediments of the pockmark field in southwestern Xisha Uplift, northwestern South China Sea. *Mar. Petr. Geol.* **48**, 247-259 (2013).

### 30. Junggar Basin (Onshore China)

Ji, L., et al. Characteristics of mixed sporopollen assemblage from sediments of Dushanzi mud volcano in southern Junggar Basin and indication to the source of mud and debris ejecta. *Mar. Petr. Geol.* **89**, 194-201 (2018).

Li, N., Huang, H. & Chen, D. Fluid sources and chemical processes inferred from geochemistry of pore fluids and sediments of mud volcanoes in the southern margin of the Junggar Basin, Xinjiang, northwestern China. *Appl. Geochem.* **46**, 1-9 (2014).

### 31. Gobi Altay (Mongolia)

RukavIčková, L. & Hanžl, P. Mud volcanoes in the Khar Argalantyn Nuruu, NW Gobi Altay, Mongolia as manifestation of recent seismic activity. *J. Geosci.* **53**, 181-191 (2008).

### 32. Lake Baikal

Van Rensbergen, P., et al. Sublacustrine mud volcanoes and methane seeps caused by dissociation of gas hydrates in Lake Baikal. *Geology* **30**, 631-634 (2002).

### 33. Okhotsk Sea (Sakhalin Island, E Russia)

Ludmann, T. & Wong, H. K. Characteristics of gas hydrate occurrences associated with mud diapirism and gas escape structures in the northwestern Sea of Okhotsk. *Mar. Geol.* **201**, 269-286 (2003).

Shakirov, R., et al. Mud volcanoes and gas vents in the Okhotsk Sea area. *Geo-Mar. Lett.* **24**, 140-149 (2004).

### 34. Nankai Accretionary Wedge (Offshore Japan)

Kioka, A., Tsuji, T., Otsuka, H. & Ashi, J. Methane concentration in mud conduits of submarine mud volcanoes: A coupled geochemical and geophysical approach. *Geochem., Geophys., Geosys.* **20**, 792-813 (2019).

Menapace, W., et al. The role of mud volcanism and deep-seated dewatering processes in the Nankai Trough accretionary prism and Kumano Basin, Japan. *Geochem. Geophys. Geosys.* **18**, 2486-2509.

Pape, T., Patrizia Geprägs, Sebastian Hammerschmidt, Paul Wintersteller, Jiangong Wei, Timo Fleischmann, Gerhard Bohrmann, and Achim J. Kopf, 2014. Hydrocarbon seepage and its sources at mud volcanoes of the Kumano forearc basin, Nankai Trough subduction zone. *Geochem., Geophys., Geosyst.* **15**, 2180-2194 (2017).

### 35. Hikurangi Accretionary Wedge (N New Zealand)

Pettinga, J. R. Mud volcano eruption within the emergent accretionary Hikurangi margin, southern Hawke's Bay, New Zealand. *New Zealand J. Geol. Geophys.* **46**, 107-121 (2003).

Ridd, M. F. Mud volcanoes in New Zealand. *AAPG Bull.* **54**, 601-616, (1970).

Zeyen, H., et al. 3D electrical resistivity imaging of the near-surface structure of mud-volcano vents. *Tectonophysics* **509**, 181-190 (2011).

### 36. Ceduna Sub-Basin (Offshore S Australia)

Brown, B. J., et al. Formation and evolution of Australian passive margins: Implications for locating the boundary between continental and ocean crust, in *Evolution and Dynamics of the Australian Plate* (eds. Hillis, R. R. & Müller, R. D.) 223-243 (*GSA Spec. Papers* **372**, 2003).

Cunneen, J., Grigg, C. & Keating, E. Evolution of the outer basin high, Ceduna Sub-basin, southern Australia. *APPEA J.* **57**, 722-725 (2017).

Espurt, N., et al. Interactions between continental breakup dynamics and large-scale delta system evolution: insights from the Cretaceous Ceduna delta system, Bight Basin, Southern Australian margin. *Tectonics* **28**, TC6002 (2009).

Hill, K. C., Cunneen, J. & Farrington, R. The Bight Basin, evolution and prospectivity II; seismic, structure and balanced sections. *ASEG Ext. Abst.* **2019**, 1-5 (2019).

King, R. C. & Backé, G. A balanced 2D structural model of the Hammerhead Delta–Deepwater Fold-Thrust Belt, Bight Basin, Australia. *Australian J. Earth Sci.* **57**, 1005-1012 (2010).

Kovacevic, M., Cunneen, J. & Elders, C. Evolution of detached listric fault systems in the Ceduna Delta, Bight Basin: insights from 3D seismic data. *ASEG Ext. Abst.* **2015**, 1-4 (2015).

Krassay, A. A. & Totterdell, J. M. Seismic stratigraphy of a large, Cretaceous shelf-margin delta complex, offshore southern Australia. *AAPG Bull.* **87/6**, 203-219 (2003).

Langhi, L., Strand, L. & Stuart Ross, A. Fault-related biogenic mounds in the Ceduna Sub-basin, Australia. Implications for hydrocarbon migration. *Mar. Petr. Geol.* **74**, 47-58 (2016).

Robson, A., King, R. C. & Holford, S. P. Structural evolution of a gravitationally detached normal fault array: analysis of 3D seismic data from the Ceduna Sub-Basin, Great Australian Bight. *Bas. Res.* **29**, 605-624 (2017).

Strand, J., Langhi, L., Stuart Ross, A. & Dyt, C. Coupled stratigraphic and fault seal modelling used to describe trap integrity in the frontier Bight Basin, Australia. *Mar. Petr. Geol.* **86**, 474-485 (2017).

Totterdell, J. M. & Bradshaw, B. E. The structural framework and tectonic evolution of the Bight Basin, in *Eastern Australasian Basins Symposium II (Petr. Expl. Soc. Australia Spec. Pub*.) 41-61 (2004).

Totterdell, J. M., Bradshaw, B. E. & Wilcox, J. B. Structural and tectonic setting, in *The Petroleum of Geology of South Australia Government of South Australia* (*Depart. Energy Mining* **5**: Great Australia Bight, in press).

Totterdell, J. M. & Krassay, A. A. The role of shale deformation and growth faulting in the Late Cretaceous evolution of the Bight Basin, offshore southern Australia, in *Subsurface Sediment Mobilization* (eds. Van Rensbergen, P., et al.) 429-442 (*Geol. Soc. Spec. Publ.* **216**, 2003).

### 37. North Sea (Håkon Mosby Mud Volcano)

Andresen, K. J., Clausen, O. R. & Jørgensen, R. B. A composite mud volcano system in the Chalk Group of the North Sea Central Graben. *J. Geol. Soc.* **167**, 1209–1224 (2010).

Feseker, T., et al. The thermal structure of the Dvurechenskii mud volcano and its implications for gas hydrate stability and eruption dynamics. *Mar. Pet. Geol.* **26**, 1812-1823 (2009).

Feseker, T., Foucher, J. P. & Harmegnies, F. Fluid flow or mud eruptions? Sediment temperature distributions on Håkon Mosby mud volcano, SW Barents Sea slope, *Mar. Geol.* **247**, 194–207 (2008).

Foucher, J.-P., et al. Changes in seabed morphology, mud temperature and free gas venting at the Håkon Mosby mud volcano, offshore northern Norway, over the time period 2003–2006. *Geo-Mar. Lett.* **30**, 157-167 (2010).

Hjelstuen, B. O., Eldholm, O., Faleide, J. I. & Vogt, P. R. Regional setting of Hakon Mosby Mud Volcano, SW Barents Sea margin. *Geo-Mar. Lett.* **19**, 22–28 (1999).

Hovland, M. Large pockmarks, gas-charged sediments and possible clay diapirs in the Skagerrak. *Mar. Petr. Geol.* **8**, 311-316 (1991).

Hovland, M., Nygaard, E. & Thorbjørnsen, S. Piercement shale diapirism in the deep-water Vema Dome area, Voring basin, offshore Norway. *Mar. Petr. Geol.* **15**, 191-201 (1998).

Jackson, C. & Stoddart, D. Temporal constraints on the growth and decay of large-scale mobilized mud masses and implications for fluid flow mapping in sedimentary basins. *Terra Nova* **17**, 580-585 (2005).

Jerosch, K., et al. Spatial distribution of mud flows, chemoautotrophic communities, and biogeochemical habitats at Håkon Mosby Mud Volcano. *Mar. Geol.* **243**, 1-17 (2007).

Pape, T, et al. Distribution and abundance of gas hydrates in near-surface deposits of the Håkon Mosby Mud Volcano, SW Barents Sea. *Geochem., Geophys., Geosys.* **12**, Q09009.

Perez-García, C., Feseker, T., Mienert, J. & Berndt, C. The Håkon Mosby mud volcano: 330,000 years of focused fluid flow activity at the SW Barents Sea slope. *Mar. Geol.* **262**, 105-115 (2009).

Sauter, E.J., et al. Methane discharge from a deep-sea submarine mud volcano into the upper water column by gas hydrate-coated methane bubbles. *Earth Planet. Sci. Lett.* **243**, 354-365 (2006).

### 38. Upper Benue Trough (Onshore Nigeria)

Musa, O. K., et al. Tectonic control on the distribution of onshore mud volcanoes in parts of the Upper Benue Trough, northeastern Nigeria. *Contemp. Trends Geosci.* **5**, 28-45 (2016).

Musa, O. K., Kurowska, E., Schoeneich, K. & Alagbe, S A. Chemistry of groundwater from mud volcanoes in parts of Upper Benue Trough, northeastern Nigeria. *Env. Earth Sci.* **74**, 4897-4906 (2015).

Musa, O. K., Kurowska, E., Schoeneich, K. & Alagbe, S. A. Mud volcanoes on the dry land of Nigeria. *Africa Geosci. Rev.* **21**, 15-22 (2014).

### 39. Niger Delta

Ajakaiye, D. E. & Bally, A. W. Course manual and atlas of structural styles of reflection profiles from Niger Delta. *AAPG Cont. Educ. Course Note Ser.* **41**, 1-107 (2002).

Bilotti, F. & Shaw, J. H. Deep-water Niger Delta fold and thrust belt modeled as a critical-taper wedge: the influence of elevated basal fluid pressure on structural styles. *AAPG Bull*. **89**, 1475-1491 (2005).

Briggs, S. E., Cartwright, J. & Davies, R. J. Crustal structure of the deepwater west Niger Delta passive margin from the interpretation of seismic reflection data. *Mar. Petr. Geol.* **26**, 936-950 (2009).

Briggs, S. E., Davies, R. J., Cartwright, J. A. & Morgan, R. Multiple detachment levels and their control on fold styles in the compressional domain of the deepwater west Niger Delta. *Bas. Res.* **18**, 435-450 (2006).

Butler, R. W. H. & Paton, D. A. Evaluating lateral compaction in deepwater fold and thrust belts: How much are we missing from "nature's sandbox"? *GSA Today* **20**, 4-10 (2010).

Chima, K. I. Seismic stratigraphy and depositional architecture of Neogene intraslope basins, offshore western Niger Delta. *Mar. Petr. Geol.* **109**, 449-468 (2019).

Clark, I. R. & Cartwright, J. A. Interactions between coeval sedimentation and deformation from the Niger delta deepwater fold belt, in *Application of the Principles of Seismic Geomorphology to Continental-Slope and Base-of-Slope Systems: Case Studies from Seafloor and Near-Seafloor Analogues* (eds. Prather, B. E., et al.) (*SEPM Spec. Pub.* **No. 99**, 243-267, 2012).

Cobbold, P. R., Clarke, B. J. & Løseth, H. Structural consequences of fluid overpressure and seepage forces in the outer thrust belt of the Niger Delta. *Petr. Geosci.* **15**, 3-15 (2009).

Cohen, H. A. & McClay, K. Sedimentation and shale tectonics of the northwestern Niger Delta front. *Mar. Petr. Geol.* **13**, 313-328 (1996).

Corredor, F., Shaw, J. H. & Bilotti, F. Structural styles in the deep-water fold and thrust belts of the Niger Delta. *AAPG Bull.* **89**, 753-780 (2005).

Damuth, J. E. Neogene gravity tectonics and depositional processes on the deep Niger Delta continental margin. *Mar. Petr. Geol.* **11**, 320-346 (1994).

Dean, S., Morgan, J. & Brandenburg, J. P. Influence of mobile shale on thrust faults: insights from discrete element simulations. *AAPG Bull.* **99**, 403-432 (2015).

Graue, K. Mud volcanoes in deepwater Nigeria. *Mar. Petr. Geol.* **17**, 954-974 (2000).

Heiniö, P. & Davies, R. J. Degradation of compressional fold belts: deep-water Niger Delta. *AAPG Bull.* **90**, 753-770 (2006).

Hopper, R. J., Fitzsimmons, R. J., Grant, N. & Vendeville, B. C. The role of deformation in controlling depositional patterns in the south-central Niger Delta, West Africa. *J. Struc. Geol.* **24**, 847-859 (2002).

Jolly, B. A., Lonergan, L. & Whittaker, A. C. Growth history of fault-related folds and interaction with seabed channels in the toe-thrust region of the deep-water Niger delta. *Mar. Petr. Geol.* **70**, 58-76 (2016).

Khani, H. F. & Back, S. The influence of pre-existing structure on the growth of syn-sedimentary normal faults in a deltaic setting, Niger Delta. *J. Struc. Geol.* **73**, 18-32 (2015).

Kostenko, O. V., et al. Structural evaluation of column-height controls at a toe-thrust discovery, deep-water Niger Delta. *AAPG Bull.* **92**, 1615-1638 (2008).

Krueger, S. W. & Grant, N. T. The growth history of toe thrusts of the Niger Delta and the role of pore pressure, in *Thrust Fault-Related Folding* (eds. McClay, K., Shaw, J. & Suppe, J.) 357-390 (*AAPG Mem.* **94**, 2011).

Maloney, D., Davies, R., Imber, J. & King, S. Structure of the footwall of a listric fault system revealed by 3D seismic data from the Niger Delta. *Bas. Res.* **24**, 107-123 (2012).

Maloney, D., et al. New insights into deformation mechanisms in the gravitationally driven Niger Delta deep-water fold and thrust belt. *AAPG Bull.* **94**, 1401–1424 (2010).

Mascle, J. R., Bornhold, B. D. & Renard, V. Diapiric structures off Niger Delta. *AAPG Bull.* **57**, 1672-1678 (1973).

Pizzi, M., Lonergan, L., Whittaker, A. C. & Mayall, M. Growth of a thrust fault array in space and time: An example from the deep-water Niger delta. *J. Struc. Geol*. 137, 104088 (2020).

Restrepo-Pace, P. A. "Ductile v. Brittle"—Alternative structural interpretations for the Niger Delta, in *Passive Margins: Tectonics, Sedimentation and Magmatism* (eds. McClay, K. R. & Hammerstein, J. A.) 193-204 (*Geol. Soc. Spec. Publ.* **476**, 2020).

Rouby, D., et al. Gravity driven deformation controlled by the migration of the delta front: the Plio-Pleistocene of the Eastern Niger Delta. *Tectonophysics* **513**, 54-67 (2011).

Swarbrick, R., O'Connors, S. & Lahann, R. Occurrence and prediction of high-pressure sediment along the West African margin. *Lead. Edge* **June**, 682-687 (2011).

Wiener, R. W., Mann, M. G., Angelich, M. T. & Molyneux, J. B. Mobile shale in the Niger Delta: characteristics, structure, and evolution, in *Shale Tectonics* (ed. Wood, L.) 145-161 (*AAPG Mem.* **93**, 2010).

Wu, J. E., McClay, K. & Frankowicz, E. Niger Delta gravity-driven deformation above the relict Chain and Charcot oceanic fracture zones, Gulf of Guinea: insights from analogue models. *Mar. Petr. Geol.* **65**, 43-62 (2015).

Wu, S. & Bally, A. W. Slope tectonics—comparisons and contrasts of structural styles of salt and shale tectonics of the northern Gulf of Mexico with shale tectonics of offshore Nigeria in Gulf of Guinea, in *Atlantic Rifts and Continental Margins* (eds. Mohriak, W. & Talwani, M.) 151-172 (Washington, D.C., AGU, 2000).

## 40. Orange Basin (Offshore Namibia and South Africa)

Ben-Avraham, Z., Reshef, M. & Smith, G. Seismic signature of gas hydrate and mud volcanoes of the South African continental margin, in *Mud Volcanoes, Geodynamics and Seismicity* (eds. Martinelli, G. & Panahi, B.) 17-27 (Springer, 2005).

Ben-Avraham, Z., Smith, G., Reshef, M. and Jungslager, E. H. A. Gas hydrate and mud volcanoes on the southwest African continental margin off South Africa. *Geology* **30**, 927-930 (2002).

Dalton, T. J. S., Paton, D. A., Needham, T. & Hodgson, N. Temporal and spatial evolution of deepwater fold thrust belts: implications for quantifying strain imbalance. *Interpretation* **3**, SAA59-SAA70 (2015).

de Vera, J., Granado, P. & McClay, K. Structural evolution of the Orange Basin gravity-driven system, offshore Namibia. *Mar. Petr. Geol.* **27**, 223-237 (2010).

Hartwig, A., Anka, Z. & di Primio, R. Evidence of a widespread paleo-pockmarked field in the Orange Basin: an indication of an early Eocene massive fluid escape event offshore South Africa. *Mar. Geol.* **332-334**, 222-234 (2012).

Kuhlmann, G., et al. Passive margin evolution and its controls on natural gas leakage in the southern Orange Basin, blocks 3/4, offshore South Africa. *Mar. Petr. Geol.* **27**, 973-992 (2010).

Moss, J. L. & Cartwright, J. 3D seismic expression of km-scale fluid escape pipes from offshore Namibia. *Bas. Res.* **22**, 481-501 (2010).

Paton, D. A., et al. Insights into the petroleum system evolution of the southern Orange Basin, South Africa. *S. African J. Geol.* **110**, 261-274 (2007).

Scarselli, S., McClay, K. & Elders, C. Seismic geomorphology of Cretaceous megaslides offshore Namibia (Orange Basin): insights into segmentation and degradation of gravity-driven linked systems. *Mar. Petr. Geol.* **75**, 151-180 (2016).

Viola, G., et al. Offshore mud volcanoes and onland faulting in southwestern Africa: neotectonic implications and constraints on the regional stress field. *Earth Planet. Sci. Lett.* **231**,147-160 (2005).

### 41. Labrador Margin (Saglek and Hopedale Basins) and Baffin Bay Basin (E Canada)

Carter, J. E., Cameron, D., Wright, R. & Gillis, E. New insights on the slope and deep water region of the Labrador Sea, Canada, in *Proc., 75th EAGE Conference & Exhibition incorporating SPE EUROPEC 2013* (European Association of Geoscientists & Engineers, 2013) cp-348-00990.

Dafoe, L.T., et al. *Report of Activities for the Stratigraphic and Tectonic Framework for the Baffin Bay Petroleum Systems* (*Geol. Surv. Canada* **Open File 8144**, 2016) doi:10.4095/299240.

Dickie, K., Keen, C. E., Williams, G. L. & Dehler, S. A. Tectonostratigraphic evolution of the Labrador margin, Atlantic Canada. *Mar. Petr. Geol.* **28**, 1663-1675 (2011).

Enachescu, M. & Hogg, J. Compression modified extensional structures (CMES) of the Canadian Atlantic Passive Margin. *AAPG Search & Disc*. **90211** (2006).

Funck, T., Gohl, K., Damm, V. & Heyde, I. Tectonic evolution of southern Baffin Bay and Davis Strait: results from a seismic refraction transect between Canada and Greenland. *J. Geophys. Res. Solid Earth* **117** (2012).

Gillis, E., Wright, R., Mitchell, V. & Montevecchi, N. Definition of the Churchill River Delta and its petroleum potential, offshore Labrador, Canada. *Interpretation* **8**, SH19-SH32 (2020).

Harrison, J. C., Brent, T. A. & Oakey, G. N. Baffin Fan and its inverted rift system of Arctic eastern Canada: stratigraphy, tectonics and petroleum resource potential, in *Arctic Petroleum Geology* (eds. Spencer, A. M., et al.) 595-626 (*Geol. Soc. Mem.* **35**, 2011).

Keen, C. E., Dickie, K. & Dafoe, L. T. Structural characteristics of the ocean-continent transition along the rifted continental margin, offshore central Labrador. *Mar. Petr. Geol.* **89**, 443-463 (2018).

Schenk, C. J. Geology and petroleum potential of the West Greenland–East Canada Province, in *Arctic Petroleum Geology* (eds. Spencer, A. M., et al.) 627- 645 (*Geol. Soc. Mem.* **35**, 2011).

Welford, J. K., et al. Crustal structure of Baffin Bay from constrained three-dimensional gravity inversion and deformable plate tectonic models. *Geophys. J. Int.* **214**, 1281-1300 (2018).

### 42. Offshore Nova Scotia (Sable Subbasin, E Canada)

Deptuck, M. E. & and Altheim, B. *Rift Basins of the Central LaHave Platform, Offshore Nova Scotia*. (CNSOPB Geosci. **Open File 2018-001MF**, 2018).

Deptuck, M. E. & Kendell, K. L. A review of Mesozoic-Cenozoic salt tectonics along the Scotian Margin, Eastern Canada, in *Permo-Triassic Salt Provinces of Europe, North Africa and the Atlantic Margins* (eds. Soto, J. I., Flinch, J. F. & Tari, G.) 287-312 (Elsevier, 2017) doi:10.1016/B978-0-12-809417-4.00014-8.

Kendell, K. L. Variations in salt expulsion style within the sable canopy complex, central Scotian margin. *Canadian J. Earth Sci.* **49**, 1504-1522 (2012).

## 43. Port-Isabel Folded Belt and Northern Gulf of Mexico

Camerlo, R. H. & Benson, E. F. Geometric and seismic interpretation of the Perdido fold belt: northwestern deep-water Gulf of Mexico. *AAPG Bull.* **90**, 363-386 (2006).

Camerlo, R., Meyer, D. & Meltz, R. Shale tectonics in the Northern Port Isabel Fold Belt trend, deepwater Gulf of Mexico. *Houston Geol. Soc. Bull.* **February**, 57-59 (2005).

Camerlo, R., Meyer, D. & Meltz, R. Shale tectonism in the northern Port Isabel Fold Belt, in *Salt-Sediment Interactions and Hydrocarbon Prospectivity: Concepts, Applications and Case Studies for the 21st Century* (eds. Post, P. J., et al.) 817-839 (*24th Ann. Gulf Coast Sec. SEPM Foundation, Bob F. Perkins Res. Conf.*, 2004).

Castellini, G., 2004. *Barium Cycling in Shallow Sediment above Active Mud Volcanoes in the Gulf of Mexico.* (Rice University, 2006).

Ellis, M., et al. Electromagnetic surveying of seafloor mounds in the northern Gulf of Mexico. *Mar. Petr. Geol.* **25**, 960-968 (2008).

Ewing, T. E. *Structural Styles of the Wilcox and Frio Growth-Fault Trends in Texas: Constraints on Geopressured Reservoirs* (Bur. Econ. Geol. **RI 154**, 1986).

Galloway, W. E., Hobday, D. K. & Magara, K. Frio Formation of Texas Gulf Coastal Plain: depositional systems, structural framework, and hydrocarbon distribution. *AAPG Bull.* **66**, 649-688 (1982).

Kohl, B. & Roberts, H. H. Fossil foraminifera from four active mud volcanoes in the Gulf of Mexico. *Geo-Mar. Lett.* **14**, 126-134 (1994).

Kohl, B. & Roberts, H. H. Mud volcanoes in the Gulf of Mexico: a mechanism for mixing sediments of different ages in slope environments: *GCAGS Trans.* **45**, 351-359 (1995).

MacDonald, I. R., et al. Chemosynthetic mussels at a brine-filled pock-mark in the northern Gulf of Mexico. *Science* **248**, 1096-1099 (1990).

MacDonald, I. R., et al. Gas hydrate that breaches the sea floor on the continental slope of the Gulf of Mexico. *Geology* **22**, 699-702 (1994).

MacDonald, I. R., et al. Pulsed oil discharge from a mud volcano. *Geology* **28**, 907-910 (2000).

MacDonald, I. R. & Peccini, M. B. Distinct activity phases during the recent geologic history of a Gulf of Mexico mud volcano. *Mar. Petr. Geol.* **26**, 1824-1830 (2009).

McDonnell, A., Hudec, M. R. & Jackson, M. P. A. Distinguishing salt welds from shale detachments on the inner Texas shelf, western Gulf of Mexico, *Bas. Res.* **21**, 47-59 (2009).

McDonnell, A., Jackson, M. P. A. & Hudec, M. R. Origin of transverse folds in an extensional growth-fault setting: evidence from an extensive seismic volume in the western Gulf of Mexico. *Mar. Petr. Geol.* **27**, 1494-1507 (2010).

Morgan, J. P., Coleman, J. M. & Gagliano, S. M. Mudlumps: Diapiric Structures in Mississippi Delta Sediments. *AAPG Mem. (Diapirism and Diapirs)* **8**, 145-161 (1968).

Neurauter, T. W. & Bryant, W. R. Seismic expression of sedimentary volcanism on the continental slope, northern Gulf of Mexico. *Geo-Mar. Lett.* **10**, 225-231 (1990).

Neurauter, T. W. & Roberts, H. H. Three generations of mud volcanoes on the Louisiana continental slope. *Geo-Mar. Lett.* **14**, 120-125 (1994).

Roberts, H .H. & Carney, R. S. Evidence of episodic fluid, gas, and sediment venting on the northern Gulf of Mexico continental slope. *Bull. SEG* **92**, 863-879 (1997).

Roberts, H. H. & Neurauter, T. W. Direct observations of a large active mud vent on the Louisiana continental slope. *AAPG Bull.* **74**, 1508 (1990).

## 44. Delta del Bravo (W Gulf of Mexico, USA and Mexico)

Alzaga-Ruiz, H., et al. Gravitational collapse and Neogene sediment transfer across the western margin of the Gulf of Mexico: insights from numerical models. *Tectonophysics* **470**, 21-41 (2009a).

Alzaga-Ruiz, H., Granjeon, D. & Roure, F. Gravitational collapse and Neogene sediment transfer across the Western Margin of the Gulf of Mexico: insights from numerical models. *GCAGS Trans.* **61**, 535-538 (2011).

Alzaga-Ruiz, H., Lopez, M., Roure, F. & Séranne, M. Interactions between the Laramide Foreland and the passive margin of the Gulf of Mexico: tectonics and sedimentation in the Golden Lane area, Veracruz State, Mexico. *Mar. Petr. Geol.* **26**, 951-973 (2009b).

Echanove, O. Geología petrolera de la Cuenca de Burgos (Parte I): consideraciones geológico-petroleras. *Boletín de la Asociación Mexicana de Geólogos Petroleros* **XXXVIII**, 3-39 (1986).

Echanove, O. Geología petrolera de la Cuenca de Burgos (Parte II): resultado de pozos exploratorios y antecedentes de campos y producción. *Boletín de la Asociación Mexicana de Geólogos Petroleros* **XXXVIII**, 40-74 (1986b).

Eguiluz de Antuñano, S. Sinopsis geológica de la Cuenca de Burgos, noreste de México: producción y recursos petroleros. *Boletín de la Sociedad Geológica Mexicana* **62**, 323-332 (2011).

Eguiluz de Antuñano, S. The Yegua Formation: gas play in the Burgos Basin, Mexico, in *Petroleum Systems in the Southern Gulf of Mexico* (eds. Bartolini, C. & Román Ramos, J. R.) 49-77 (*AAPG Mem.*, 2009).

Flotté, N. et al. The Rio Bravo fault, a major late Oligocene left-lateral shear zone. *Bulletin de la Sociéte Géologique de France* **179**, 147-160 (2008).

Gracia-Marroquín, D. A. *Simulación de Sistemas Gravitacionales Ligados Extensión-Acortamiento en la Zona del Golfo de México Norte, Mediante Modelos Analógicos* (Universidad Nacional Autónoma de México, Ciudad de México, 2013).

Gracia-Marroquín, D., Cerca, M., Carreón-Freyre, D. & Barrientos-García, B. Analogue model of gravity driven deformation in the salt tectonics zone of northeastern Mexico. *Revista Mexicana de Ciencias Geológicas* **35**, 277-290 (2018).

Gutiérrez Moreno, H. & Rodríguez Otero, M. Comparativa estructural, cinemática e implicaciones petroleras de cinturones plegados con despegue en arcilla en aguas profundas del Golfo de México, in *Congreso Mexicano del Petróleo, CMP, León* 1-17 (2019).

Hernández Flores, E. R. Delta del Bravo ¿Tectónica de sal o de arcilla? *Boletín de la Asociación Mexicana de Geólogos Petroleros* **L**, 21-33 (2003).

Hernández-Mendoza, J. J., DeAngelo, M. V., Wawrzyniec, T. F. & Hentz, T. F. Major structural elements of the Miocene section, Burgos Basin, northeastern Mexico. *AAPG Bull.* **92**, 1479-1499 (2008).

Hudec, M. R., Dooley, T. P., Peel, F. J. & Soto, J. I. Controls on the evolution of passive-margin salt basins: Structure and evolution of the Salina del Bravo region, northeastern Mexico. *Geol. Soc. Am. Bull.* **132**, 997–1012 (2020).

Le Roy, C., et al. Neogene crustal shear along the western Gulf of Mexico margin and its implications for gravity sliding processes: evidences from 2D and 3D multichannel seismic data. *Bulletin de la Sociéte Géologique de France* **179**, 175-193 (2008).

Le Roy, C. & Rangin, C. Cenozoic crustal deformation of the offshore Burgos basin region (NE Gulf of Mexico): a new interpretation of deep penetration multichannel seismic reflection lines. *Bulletin de la Sociéte Géologique de France* **179**, 161-174 (2008).

Ortíz-Ubilla, A. & G. Tolson Interpretación estructural de una sección sísmica en la región Arcabuz–Culebra de la Cuenca de Burgos, NE de México. *Revista Mexicana de Ciencias Geológicas* **21**, 226-235 (2004).

Palmes, S. L. Shale-detached deformation along the Texas Shelf: 3D Analyses, recognition criteria, and development of duplex structures in extensional settings, in *Salt-Sediment Interactions and Hydrocarbon Prospectivity: Concepts, Applications and Case Studies for the 21st Century* (eds. Post, P. J., et al.) 779-815 (*24th Ann. Gulf Coast Sec. SEPM Foundation, Bob F. Perkins Res. Conf.*, 2004).

Pérez-Cruz, G. A. *Geologic Evolution of the Burgos Basin, Northeastern Mexico* (Rice University, Houston, 1992).

Peterson Rodríguez, R. H., et al. Structural characterization and tectonic evolutional onshore-offshore model at the Mexican Northwestern portion of the Gulf of Mexico. *GCAGS J.* **61**, 341-352 (2011).

Rangin, C., Le Pichon, X., Flotté, N. & Husson, L. Cenozoic gravity tectonics in the northern Gulf of Mexico induced by crustal extension: a new interpretation of multichannel seismic data. *Bulletin de la Sociéte Géologique de France* **179**, 117-128 (2008a).

Rangin, C., Le Pichon, X., Martínez-Reyes, J. & Aranda-García, M. Gravity tectonics and plate motions: the western margin of the Gulf of Mexico. Introduction. *Bulletin de la Sociéte Géologique de France* **179**, 107-116 (2008b).

Shaker, S. S. & Wornardt, W. W., Jr. The prospective deep Miocene in the Gulf of Mexico shelf: compartmentalization knowledge of the geopressured depositional system sheds light on immense exploration potential, in *Shelf Margin Deltas and Linked Down Slope Petroleum Systems–Global Significance and Future Exploration Potential* (eds. Roberts, H. H., Rosen, N. C., Fillon, R. H. & Anderson, J. B.) 675-692 (*23rd. Ann. GCSSEPM Foundation Bob F. Perkins Res. Conf.* **23**, 2013).

**45. Mexican Ridges (W Gulf of Mexico, Mexico)**

Buffler, R. T., Shaub, F. J., Watkins, J. S. & Worzel, J. L. Anatomy of the Mexican Ridges, Southwestern Gulf of Mexico, in *Geological and Geophysical Investigations of Continental Margin* (eds. Watkins, J. S., Montadert, L. & Dickerson, P. W.) 319-327 (AAPG, 1978).

Fitz-Diaz, E., Huddleston, P. & Tolson, G. Comparison of tectonic styles in the Mexican and Canadian Rocky Mountain fold-thrust belt, in *Kinematic Evolution and Structural Styles of Fold-and-Thrust Belts* (eds. Poblet J. &Lisle, R. J.) 149-167 (Geol. Soc. London, 2011).

Jackson, M. P. A. & Galloway, W. E. Tertiary Gulf Coast gravity-glide folding and faulting: the Mexican Ridges: Unit 31: application to Gulf Coast Tertiary, in *Structural and Depositional Styles of Gulf Coast Tertiary Continental Margins: Application to Hydrocarbon Exploration* (eds. M. P. A. Jackson and W. E. Galloway) 177-182 (*AAPG Cont. Ed. Course Note Series* **25**, 1984).

Pew, E., *Seismic Structural Analysis of Deformation in Southern Mexican Ridges* (The University of Texas at Austin, 1982).

Salomón-Mora, L. E. *Structure and Tectonics of the Salt and Shale Provinces, Western Gulf of Mexico* (University of Aberdeen, 2013).

Salomón-Mora, L. E., Aranda-García, M. & Román Ramos J. R. Contractional growth faulting in the Mexican Ridges, Gulf of Mexico, in *Petroleum Systems in the Southern Gulf of Mexico* (eds. Bartolini, C. & Román Ramos, J. R.) 93-115 (*AAPG Mem.* 2009).

Salomón-Mora, L. E., Cruz Mercado, M. A., Alsop, & Archer, S. G. Tectonics, structure, and hydrocarbon potential of the Mexican Ridges Fold Belt, western Gulf of Mexico. *GCAGS Trans.* **61**, 403-419 (2011).

Tharp, T. M. & Scarbrough, M. G. Application of hyperbolic stress-strain models for sandstone and shale to fold wavelength in the Mexican Ridges foldbelt. *J. Struc. Geol*. **16**, 1603-1618 (1994).

Yarbuh, I. & J. Contreras The interplay between deformation, erosion and sedimentation in the deep-water Mexican Ridges foldbelt, western Gulf of Mexico basin. *Bas. Res.* **29**, 446-464 (2017) doi:10.1111/bre.12157.

**46. Comalcalco, Macuspana and Salina del Istmo Basins (S Gulf of Mexico, Mexico)**

Ambrose, W. A., et al. Geologic framework of upper Miocene and Pliocene gas plays of the Macuspana Basin, southeastern Mexico, *AAPG Bull.* **87**, 1411-1435 (2000).

Ambrose, W., et al. The Macuspana Basin, South Gulf of Mexico: impact of shale tectonics and late contraction of an extensional basin on hydrocarbon accumulation. *AAPG Search & Disc.* **90013©92003** (2003).

Ambrose, W., et al. Neogene tectonic, stratigraphic, and play framework of the southern Laguna Madre–Tuxpan continental shelf, Gulf of Mexico. *AAPG Bull.* **89**, 725-751 (2005).

Ding, F., et al. Shallow sediment deformation styles in north-western Campeche Knolls, Gulf of Mexico and their controls on the occurrence of hydrocarbon seepage. *Mar. Petr. Geol.* **27**, 959-972 (2010).

MacDonald, I. R., et al. Asphalt volcanism and chemosynthetic life in the Campeche knolls, Gulf of Mexico. *Science* **304**, 999-1002 (2004).

Chavez Valois, V. M., et al. A new multidisciplinary focus in the study of the Tertiary plays in the Sureste Basin, Mexico, in *Petroleum Systems in the Southern Gulf of Mexico* (eds. Bartolini, C. & Román Ramos, J. R.) 155-190 (*AAPG Mem.*, 2009).

Chávez Valois, V. M., et al. Un nuevo enfoque multidisciplinario en el estudio de "plays": el Terciario de la Cuenca del Sureste, Tabasco, México. *Boletín de la Sociedad Geológica Mexicana* **LII**, 5-32 (2005).

Gómez-Cabrera, P. T. & Jackson, M. P. A. Regional Neogene salt tectonics in the offshore Salina del Istmo Basin, Southeastern Mexico, in *Petroleum Systems in the Southern Gulf of Mexico* (eds. Bartolini, C. & Román Ramos, J. R.) 1-28 (*AAPG Mem.*, 2009).

Gutiérrez Moreno, H. & Rodríguez Otero, M. Comparativa estructural, cinemática e implicaciones petroleras de cinturones plegados con despegue en arcilla en aguas profundas del Golfo de México, in *Congreso Mexicano del Petróleo* 1-17 (CMP, León, 2019).

Pindell, J., and E. Miranda (2011), Linked kinematic histories of the Macuspana, Akal-Reforma, Comalcalco, and deepwater Campeche Basin tectonic elements, Southern Gulf of Mexico. *GCAGS Trans.* **61**, 353-361.

## 47. Puerto Rico and Hispaniola Accretionary Wedges

Dillon, W. P., et al. Accretionary margin of northwestern Hispaniola: morphology, structure and development of part of the northern Caribbean plate boundary. *Mar. Petr. Geol.* **6**, 70-88 (1992).

Granja Bruña, J. L., et al. Gravity modeling of the Muertos Trough and tectonic implications (north-eastern Caribbean. *Mar. Geophys. Res.* **31**, 263-283 (2010).

Granja Bruña, J. L., et al. Morphotectonics of the central Muertos thrust belt and Muertos Trough (northeastern Caribbean). *Mar. Geol.* **263**, 7-33 (2009).

## 48. Barbados Accretionary Wedge

Biju-Duval, B., et al. Multibeam bathymetric survey and high resolution seismic investigations on the Barbados Ridge complex (Eastern Caribbean): A key to the knowledge and interpretation of an accretionary wedge. *Tectonophysics* **86/1–3**, 275-304 (1982).

Brown, K. M. & Westbrook, G. K. The tectonic fabric of the Barbados Ridge accretionary complex. *Mar. Petr. Geol.* **4**, 71-81 (1987).

Deville, E., et al. From frontal subduction to a compressional transform system: new geophysical data on the structure of the Caribbean-South America plate boundary in southeastern Caribbean. *Mem.VIII Simposio Bolivariano* **Paper No. 6** (2003a).

Deville, E., et al. Lateral changes of frontal accretion and mud volcanism processes in the Barbados accretionary prism and some implications, in *The Circum-Gulf of Mexico and the Caribbean: Mexico and the Caribbean Region: Hydrocarbon Habitats, Basin Formation, Plate Tectonics* (eds. Bartolini, C., Buffler, T. & Blickwede, J. F.) 119 (*AAPG Mem.* **79**, 2003b).

Deville, E., et al. Liquefied vs. stratified sediment mobilization processes: insight from the south of the Barbados accretionary prism. *Tectonophysics* **428**, 33-47 (2006).

Deville, E., et al. Mud volcanism origin and processes: new insights from Trinidad and the Barbados prism, in *Subsurface Sediment Mobilization* (eds. Van Rensbergen, P., et al.) 477-492 (*Geol. Soc. Spec. Publ.* **216**, 2003c).

Deville, E., Guerlais, S.-H., Lallemant, S. & Schneider, F. Fluid dynamics and subsurface sediment mobilization processes: an overview from Southeast Caribbean. *Bas. Res.* **22**, 361-379 (2010).

Deville, E. & Mascle, A. The Barbados ridge: A mature accretionary wedge in front of the Lesser Antilles active margin, in *Phanerozoic Regional Geology of the World* (eds. Bally, A. W. & Roberts, D. G.) 580-607 (Elsevier, 2012).

Griboulard, R., Bobier, C., Faugeres, J. C. & Vernette, G. Clay diapiric structures within the strike-slip margin of the southern Barbados prism. *Tectonophysics* **192**, 383-400 (1991).

Lance, S., et al. Submersible study of mud volcanoes seaward of the Barbados accretionary wedge: sedimentology, structure and rheology. *Mar. Geol.* **145**, 255-292 (1998).

Martin, J. B., et al. Chemical and isotopic evidence for sources of fluids in a mud volcano field seaward of the Barbados accretionary wedge. *J. Geophys. Res.* **101**, 20325-20345 (1996).

Mascle, A., Lajat, D. & Nely, G. Sediment deformation linked to subduction and to argilokinesis in the southern Barbados Ridge from multichannel seismic surveys, in *Trans. 4th Latin Am. Geol. Conf.* 873-882 (Port-of-Spain, Trinidad and Tobago, 1979).

Sullivan, S., Wood, L. J. & Mann, P. Distribution, nature and origin of mobile mud features offshore Trinidad. *GCSSEPM Mem.* **24**, 498-513 (2004a).

Sullivan, S., Wood, L.J. & Mann, P. Distribution, nature and origin of mobile mud features Offshore Trinidad, in *Salt-Sediment Interactions and Hydrocarbon Prospectivity: Concepts, Applications and Case Studies for the 21st Century* (eds. Post, P. J., et al.) 840-867 (*24th Ann. Gulf Coast Sec. SEPM Foundation, Bob F. Perkins Res. Conf.*, 2004b).

Westbrook, G. K. & Smith, M. J. Long decollements and mud volcanoes: evidence from the Barbados Ridge Complex for the role of high pore-fluid pressure in the development of an accretionary complex. *Geology* **11**, 279-283 (1983).

## 49. Trinidad and Tobago

Arnold, R. & MacReady, G. A. Island-forming mud volcano in Trinidad, British West Indies. *AAPG Bull.* **40**, 2748-2758 (1956).

Bower T. H., Mudflow occurrence in Trinidad (BWI). *AAPG Bull.* **35**, 908-912 (1951).

Deville, E., et al. Deep-water erosion processes in the Orinoco turbidite system. *Offshore* **63**, 92-96 (2003).

Deville, E., et al. Processes of shale diapirism and mud volcanism in the Barbados-Trinidad compressional system: integrated structural, thermal and geochemical approach. *GCSSEPM Memoir* **24**, 514-527 (2004).

Deville, E. & Guerlais, S.-H. Cyclic activity of mud volcanoes: evidences from Trinidad (SE Caribbean). *Mar. Petr. Geol.* **26**, 1681-1691 (2009).

Dia, A. N., Castrec-Rouelle, M., Boulègue, J. & Comeau, P. Trinidad mud volcanoes: where do the expelled fluids come from? *Geochim. Cosmochim. Acta* **63**, 1023-1038 (1999).

Heppard, P. D., Cander, H. S. & Eggertson, E. B. Abnormal pressure and the occurrence of hydrocarbons in offshore eastern Trinidad, West Indies, in *Abnormal Pressures in Hydrocarbon Environments* (eds. Law, B. E., Ulmishek, G. F. & Slavin, V. I.) 215-246 (*AAPG Mem*., 1998.

Higgins, G. E. & Saunders, J. B. Mud volcanoes, their nature and origin. *Contr. Geol. Paleobiol. Carib. Adj. Areas* **84**, 101-15 (1974).

Higgins, G. E. & Saunders, J. B. Report on 1964 Chatham Mud Island, Erin Bay, Trinidad, West Indies. *AAPG Bull.* **51**, 55-64 (1967).

Hosein, R., Haque, S. & Beckles, D. M. Mud Volcanoes of Trinidad as astrobiological analogs for Martian environments. *Life* **4**, 566-585 (2014) doi:10.3390/life4040566.

Kerr, P. F., Drew, I. M. & Richardson, D. S. Mud volcano clay, Trinidad, West Indies. *AAPG Bull.* **54**, 2101-2110 (1970).

Kugler, H. G. Contribution to the knowledge of sedimentary volcanism in Trinidad. *J. Inst. Petr. Technols.* **19**, 743-772 (1933).

Suter, H. H. The general and economic geology of Trinidad. B.W.I. *Col. Geol. Min. Res.* **2**, 177-307 (1951).

Suter, H. H. The general and economic geology of Trinidad, B.W.I. *Col. Geol. Min. Res.* **3**, 3-51 (1952).

Suter, H. H. The general and economic geology of Trinidad, B.W.I., 2nd ed. (*HMSO*, London) (1960).

Wood, L. J., Sullivan, S. & Mann. P. Influence of mobile shales in the creation of successful hydrocarbon basins, in *Salt-Sediment Interactions and Hydrocarbon Prospectivity: Concepts, Applications and Case Studies for the 21st Century* (eds. Post, P. J., et al.) 892-930 (*24th Ann. Gulf Coast Sec. SEPM Foundation, Bob F. Perkins Res. Conf.*, 2004).

Yassir, N. A. Mud volcanoes: evidence of neotectonic activity, in *First Sino-British Geological Conference on Geotechnical Engineering and Hazard Assessment in Neotectonic Terrains* (eds. Hung, J.-J., et al.) 513-524 (*Mem. Geol. Soc. China* **9**, 1987).

## 50. Maturin Basin (Onshore Venezuela)

Bennett, J., et al. The Pedernales field: unraveling reservoir complexity, in *Sociedad Venezolana de Ingenieros Geofísicos, Memorias del VII Congreso Venezolano de Geofísica*, 486-493 (1994).

Di Croce, J. *Eastern Venezuela Basin: Sequence Stratigraphy and Structural Evolution* (Rice University Houston, 1995).

Duerto, L. *Shale Tectonics, Eastern Venezuelan Basin* (Royal Holloway, University of London, 2007).

Duerto, L. & McClay, K. Role of the shale tectonics on the evolution of the Eastern Venezuelan Cenozoic thrust and fold belt. *Mar. Petr. Geol.* **28**, 81-108 (2011).

Duerto, L. & McClay, K. The role of syntectonic sedimentation in the evolution of doubly vergent thrust wedges and foreland folds. *Mar. Petr. Geol.* **26**, 1051-1069 (2009).

Hung, E. J. *Foredeep and Thrust Belt Interpretation of the Maturin Sub-Basin, Eastern Venezuela Basin* (Rice University, 1997).

Hung, E. J. Thrust belt interpretation of the Serranía del Interior and Maturín subbasin, eastern Venezuela, in *Caribbean-South American Plate Interactions, Venezuela* (eds. Avé Lallemant, H. G. & Sisson, V. B.) 251-270 (*GSA Spec. Paper* **394**, 2005).

Jácome, M. I., Kusznir, N., Audemard, F. &Flint, S. Formation of the Maturín Foreland Basin, eastern Venezuela: Thrust sheet loading or subduction dynamic topography. *Tectonics* **22** (2003).

Nunez, M., et al. Deformation due to shale tectonic in Northwestern Venezuela, in *AAPG Int. Conf., Paris* (2005).

Parra, M., et al. The Monagas fold–thrust belt of eastern Venezuela: part I, structural and thermal modelling. *Mar. Petr. Geol.* **28**, 40-69 (2011).

Roure, F., Carnevali, J. O., Gou, Y. & Subieta, T. Geometry and kinematics of the North Monagas thrust belt (Venezuela). *Mar. Petr. Geol.* **11**, 347-362 (1994).

## 51. Magdalena and Orinoco Deltas. Sinu Folded Belt (Offshore N Colombia)

Aristizábal, C. O., Ferrari, A. L. & Silva, C.G. Control neotectónico del diapirismo de lodo en la región de Cartagena, Colombia. *Ingeniería Investigación y Desarrollo* **8**, 42-50 (2009).

Aslan, A., et al. Mud volcanoes of the Orinoco Delta, Eastern Venezuela. *Geomorphology* **41**, 323-336 (2001).

Briceño, L.A. & Vernette, G. Manifestaciones del diapirismo arcilloso en el margen colombiano del Caribe. *Earth Sci. Res. J.* **1**, 21-30 (1992).

Duque-Caro, H. Structural style, diapirism and accretionary episodes of Sinú-San Jacinto Terrane, South Western Caribbean border in *The Caribbean-South American Plate Boundary and Regional Tectonics* (eds. Bonini, W., Hargraves, R. B. & Shagam, R.) 303-316 (*GSA Mem.* **162**, 1984).

Galindo, P. A. & Lonergan, L. Basin evolution and shale tectonics on an obliquely convergent margin: The Bahia Basin, offshore Colombian Caribbean. *Tectonics* **39**, e2019TC005787 (2020).

Flinch, J. F. Structural Evolution of the Sinu-Lower Magdalena Area (Northern Colombia), in *The Circum-Gulf of Mexico and the Caribbean: Hydrocarbon Habitats, Basin Formation, and Plate Tectonics* (eds. C. Bartolini, C., Buffler, R. T. & Blickwede, J.) 776-796 (*AAPG Mem.*, 2003).

García-González, M., Bernal-Olaya, R., Fuentes-Lorenzo, J. L. & García-Ceballos, A. M. Mud diapirs and mud volcanoes associated with gas hydrates system in the Sinu Fold Belt of Colombia, South Western Caribbean and its significant in the petroleum system, in *Gas Hydrates—from Potential Geohazard to Carbon-Efficient Fuel* (AAPG Asia Pacific Reg. Geosci. Tech. Workshop, Auckland, New Zeeland, 2019).

Hernández, R. & Guerrero, C. *Expresión Profunda de Dominios Oceánico y Continental, y Propagación de su Deformación hacia la Cobertera Sedimentaria del "Offshore" Caribe* (ACGGP, 2006).

Moreno, O., et al. *Modelo Alternativo para el Desarrollo del Frente Deformado Costafuera del Caribe Colombiano* (ACGGP, 2009).

Ramírez, J. E., Los diapiros del mar Caribe Colombiano, in *Actas I Congreso Colombiano de Geología* 31-39 (Bogotá, 1969).

Ramírez, V., et al. Petroleum systems of the Guajira Basin, Northern Colombia, in *Petroleum Geology and Potential of the Colombian Caribbean Margin* (eds. Bartolini, C. & Mann, P.) 399-430 (*AAPG Mem.* **108**, 2015).

Reistroffer, et al. *Tectonic Interactions at the Northwest Margin of the South American Plate* (ACGGP, 2006).

Rodríguez Álvarez, I., *Estructura de la Parte Sumergida del Cinturón de Sinú y de la Parte Adyacente de la Cuenca de Colombia (Margen Caribeño al NO de Colombia)* (Universidad de Oviedo, 2020).

Shepard, F. P., Dill, R. F. & Heezen, B. C. Diapiric intrusions in foreset slope sediments off Magdalena Delta, Colombia. *AAPG Bull.* **52**, 21972207 (1968).

Vernette, et al. Mud diapirism, fan sedimentation and strike-slip faulting, Caribbean Colombian Margin. *Tectonophysics* **202**, 335-349 (1992).

Vernette, G., *La Plateforme Continentale de Caraibe de Colombie (du Debouche du Magdalena au Golfe de Morrosquillo): Importance du Diapirism Argilleux sur la Morphologie et la Sedimentation* (Universite de Bordeaux, 1986).

## 52. N Panamá Accretionary Wedge and Limón Folded Belt (N Panamá and Costa Rica)

Barboza, G., Barrientos, J. Astorga, A. Tectonic evolution and sequence stratigraphy of the central Pacific margin of Costa Rica. *Rev. Geol. América Central* **18**, 43-63 (1995).

Barboza, G., Fernández, A., Barrientos, J. & Botazzi, G. Costa Rica: petroleum geology of the Caribbean margin. *Lead. Edge* **16**, 1787-1794 (1997).

Brandes, C., et al. Anatomy of anticlines, piggy-back basins and growth strata: a case study from the Limón fold-and thrust belt, Costa Rica, in *Sedimentary Processes, Environments and Basins* (eds. Nichols, G., Williams, E. & Paola, C.) (*IAS Spec. Pub.* **38**, 2007).

Breen, N. A., Tagudin, J. E., Reed, D. L. & Silver, E. A. Mud-cored parallel folds and possible mélange development in the north Panama thrust belt. *Geology* **16**, 207-210 (1988).

Campos, L. Geology and basins history of middle Costa Rica: An intraoceanic island arc in the convergence between the Caribbean and the central pacific plates. *Tübinger Geowiss. Arb., Reihe A* **62**, (2001).

Fernández, J. A., Botazzi, G., Barboza, G. & Astorga, A. Tectónica y estratigrafía de la Cuenca Limón. *Sur. Rev. Geol. América Central, volumen especial Terremoto de Limón* 15-28 (1994).

Reed, D. L., et al. Relations between mud volcanoes, thrust deformation, slope sedimentation, and gas hydrate, offshore north Panama. *Mar. Petr. Geol.* **7**, 44-54 (1990).

Silver, E. A., Galwsky, J. & McIntosh, K. D. Variations in structure, style, and driving mechanism of adjoining segments of the North Panama deformed belt, in *Geologic and Tectonic Development of the Caribbean Plate Boundary in Southern Central America* (eds. Mann, P.) 225-234 (*GSA Spec. Paper* **295**, 1995).

Silver, E. A., Reed, D. L., Tagudin, J. E. & Heil, D. J. Implications of the North and South Panama thrust belts for the origin of the Panama Orocline. *Tectonics* **9**, 261-281 (1990).

## 53. Foz Do Amazonas, Barreirinhas, and Pará-Maranhão Basins (Equatorial Margin, Brazil)

Cobbold, P. R., Mourgues, R. & Boyd, K. Mechanism of thin-skinned detachment in the Amazon Fan: Assessing the importance of fluid overpressure and hydrocarbon generation. *Mar. Petr. Geol.* **21**, 1013-1025 (2004).

Darros De Matos, R. M. Tectonic evolution of the equatorial South Atlantic, in *Atlantic Rifts and Continental Margins* (eds. Mohriak, W.U. & Talwani, M.) (*AGU Geophys. Mono. Series* **115**, 331-354 (2000).

Krueger, A., Murphy, M., Gilbert, E. & Burke, K. Deposition and deformation in the deepwater sediment of the offshore Barreirinhas Basin, Brazil. *Geosphere* **8**, 1606–1631 (2012).

Oliveira, M. J. R., et al. Linked extensional-compressional tectonics in gravitational systems in the Equatorial Margin of Brazil, in (ed. Gao, D.) *Tectonics and sedimentation: Implications for petroleum Systems* 159-178 (*AAPG Mem.* **100**, 2012).

Perovano, R., et al. Modelagem física experimental de mecanismos de deformação gravitacional simulando múltiplos intervalos superpressurizados: aplicação à Bacia da Foz do Amazonas. *Revista Brasileria de Geofísica* **29**, 583-607 (2011).

Petrobrás. Foz do Amazonas Basin, offshore Brazil, in *Seismic Expression of Structural Styles* (ed. Bally, A. W.) 2.2.3-66–2.2.3-69 (*AAPG Stud. Geol.* **15**, 1984).

Reis, A. T., et al. Effects of a regional décollement level for gravity tectonics on late Neogene to recent large-scale slope instabilities in the Foz do Amazonas Basin, Brazil. *Mar. Petr. Geol.* **75**, 29-52 (2016).

Reis, A. T., et al. Two-scale gravitational collapse in the Amazon Fan: a coupled system of gravity tectonics and mass-transport processes. *J. Geol. Soc.* **167**, 593-604 (2010).

Schaller, H. & Dauzacker, M. V. Tectônica gravitacional e sua aplicação na exploração de hidrocarbonetos. *Boletim Técnico da Petrobrás* **29**, 193-206, (1986).

Silva, R., et al. O proceso de colapso gravitacional e a estruturação da seção marinha da bacia da Foz do Amazonas—Margem equatorial brasileira. *Revista Brasileria de Geofísica* **27**, 459-484 (2009).

Silva, S. R. P., Maciel, R. R. & Severino, M. C. G. Cenozoic tectonics of Amazon Mouth Basin. *Geo-Mar. Lett.* **18**, 256-262 (1999).

Tamara, J., McClay, K. R. & Hodgson, N. Crustal structure of the central sector of the NE Brazilian equatorial margin, in *Passive Margins: Tectonics, Sedimentation and Magmatism* (eds. McClay, K. R. & Hammerstein, J. A.) 163-191 (*Geol. Soc. Spec. Pub.* **476**, 2020).

Zalán, P. V. End members of gravitational fold and thrust belts (GFTBs) in the deep waters of Brazil, in *Seismic Interpretation of Contractional Fault-Related Folds* (eds. Shaw, J., Connors, C. & Suppe, J.) 147-156 (*AAPG Stud. Geol.* **53**, 2005).

Zalán, P. V. Fault-related folding in the deep waters of the Equatorial margin of Brazil in *Thrust Fault-Related Folding* (eds. McClay, K., Shaw, J. & Suppe, J.) 335-355 (*AAPG Mem.* **94**, 2011).

## 54. Camamu-Almada Basin (Offshore Brazil)

Blaich, O. A., et al. Structural architecture and nature of the continent-ocean transitional domain at the Camamu and Almada Basins (NE Brazil) within a conjugate margin setting, in *Petroleum Geology: from Mature Basins to New Frontiers* (eds. Vining, B. A. & Pickering, S. C.) 867-883 (*Proc. 7th Petr. Geol. Conf.*, 2010).

Brandão, A., Vidigal-Souza, P. & Holz, M. Evaporite occurrence and salt tectonics in the Cretaceous Camamu-Almada Basin, northeastern Brazil. *J. S. Amer. Earth Sci.* **97**, 102421 (2020).

Cobbold, P. R., et al. Large submarine slides on a steep continental margin (Camamu Basin, NE Brazil). *J. Geol. Soc.* **167**, 583-592 (2010).

Kuchle, J., Holz, M., de Brito, A. F. & Bedregal, R. P. Stratigraphic analysis of rift basins; the Camamu-Almada and Jequitinhonha basins. *Boletim de Geociências da Petrobrás* **13**, 227-244 (2005).

Loureiro, A., et al. Imaging exhumed lower continental crust in the distal Jequitinhonha basin, Brazil. *J. S. Amer. Earth Sci.* **84**, 351-372 (2018).

## 55. Beaufort-Mackenzie Basin (Offshore Artic Canada)

Bergquist, C. L., Graham, P. P., Johnston, D. H. & Rawlinson, K. R. Canada's Mackenzie delta: fresh look at an emerging basin. *Oil & Gas J.* **101**, 42-46 (2003).

Chen, Z., Osadetz, K. G. Issler, D. R. & Grasby, S. E. Hydrocarbon migration detected by regional temperature field variations, Beaufort-Mackenzie Basin, Canada. *AAPG Bull.* **92**, 1639-1653 (2008).

Dinkelman, M., et al. Highlights of petroleum and crustal framework of the Beaufort-Mackenzie Basin: key results from BeaufortSPAN East Phases I and II surveys. *Can. Soc. Expl. Geophys. Rec.* **33**, 22-25 (2008).

Dixon, J. (ed.). *Geological Atlas of the Beaufort-Mackenzie Area.* (*Geol. Surv. Can. Misc. Rep.* **59,** 1996).

Dixon, J., et al. Geology and petroleum potential of Upper Cretaceous and tertiary strata, Beaufort-Mackenzie area, Northwest Canada. *AAPG Bull.* **76**, 927-947 (1992).

Elsley, G. R. & Tieman, H. A comparison of prestack depth and prestack time imaging of the Paktoa complex, Canadian Beaufort MacKenzie Basin, in *Shale Tectonics* (ed. Wood, L.) 79-90 (*AAPG Mem.* **93**, 2010).

Helwig, J., Kumar, N., Emmet, P. & Dinkelman, M. G. Regional seismic interpretation of crustal framework, Canadian Arctic passive margin, Beaufort Sea, with comments on petroleum potential, in *Arctic Petroleum Geology* (eds. Spencer, A. M., et al.) 527-543 (*Geol. Soc. Mem.* **35**, 2011).

Issler, D. R. A new approach to shale compaction and stratigraphic restoration, Beaufort-Mackenzie Basin and Mackenzie Corridor, Northern Canada. *AAPG Bull.* **76**, 1170-1189 (1992).

Kumar, N., Helwig, J. & Dinkelman, M. G. Preliminary evaluation of a potential major petroleum province from BeaufortSPAN seismic data: Canadian Arctic passive margin, Banks Island segment. *Can. Soc. Expl. Geophys. Rec.* **34**, 26-33 (2009).

Lane, L. S. & Dietrich, J. R. Tertiary Structural evolution of the Beaufort Sea—Mackenzie Delta region, Arctic Canada. *Bull. Can. Petr. Geol.* **43**, 293-314 (1995).

McNeil, D. H., et al. A new method for recognizing subsurface hydrocarbon seepage and migration using altered foraminifera from a gas chimney in the Beaufort-Mackenzie Basin, in *Shale Tectonics* (ed. Wood, L.) 197-210 (*AAPG Mem.* **93**, 2010).

Yorath, C. J. Geology of Beaufort-Mackenzie Basin and eastern part of northern interior plains, in *Arctic Geology* (ed. Pitcher, M. G.) 41-47 (*AAPG Mem.*, 1973).

## 56. Wrangell Mountains (Onshore Alaska)

Motyka, R. J., Poreda, R. J. & Jeffrey, A. W. A. Geochemistry, isotopic composition, and origin of fluids emanating from mud volcanoes in the Copper River basin, Alaska. *Geochim. Cosmochim. Acta* **53**, 29-41 (1989).

Nichols, D. R. & Yehle, L. A. Mud volcanoes in the Copper River basin, Alaska, in *Geology of the Arctic* (eds. Raasch, G. D. & Yehle, L. A.) 1063-1087 (vol. **2**, Univ. of Toronto Press, 1961).

Patrick, M., Dean, K. & Dehn, J. Active mud volcanism observed with Landsat 7 ETM+. *J. Volcan. Geotherm. Res.* **131**, 307-320 (2004).

Sorey, M. L., Werner, C., McGimsey, R. G. & Evans, W. *Hydrothermal Activity and Carbon-Dioxide Discharge at Shrub and Upper Klawasi Mud Volcanoes, Wrangell Mountains, Alaska*. (*USGS Water-Res. Inv. Rep. 00-4207,* 2000).

Suess, E., et al. Fluid venting in the eastern Aleutian subduction zone. *J. Geophys. Res. Sol. Earth* **103**, 2597-2614 (1998).

### 57. The Queen Charlotte–Fairweather Fault System (Western British Columbia, Canada)

Barrie, J. V., Conway, K .W. & Harris, P. T. The Queen Charlotte Fault, British Columbia: seafloor anatomy of a transform fault and its influence on sediment processes. *Geo-Mar. Lett.* **33**, 311-318 (2013).

Barrie, J. V., et al. The Queen Charlotte-Fairweather fault zone; a submarine transform fault, offshore British Columbia and southeastern Alaska, cruise report of 2017003PGC CCGS Vector and 2017004PGC CCGS John P. Tully: open-file report. *Geol. Surv. Can.* **161**, 8398 (2018).

Berkowitz, R. Active mud volcano field discovered off southeast Alaska. *EOS* **96** (2015).

### 58. Cascadia Margin (W USA and Canada)

Adam, J., Klaeschen, D., Kukowski, D. N. & Flueh, E. Upward delamination of Cascadia Basin sediment infill with landward frontal accretion thrusting caused by rapid glacial age material flux. *Tectonics* **23**, TC3009 (2004).

Kulm, L. D. & Suess, E. Relationships between carbonate deposits and fluid venting: Oregon accretionary prism. *J. Geophys. Res.* **95**, 8899-8915 (1990).

McNeill, C., et al. Listric normal faulting on the Cascadia continental margin coast range. *J. Geophys. Res.* **102**, 12123-12138 (1997).

Orange, D. L. Criteria helpful in recognizing shear-zone and diapiric mélanges: examples from the Hoh accretionary complex, Olympic Peninsula, Washington. *GSA Bull.* **102**, 935-951 (1990).

Ritger, S., Carson, B. & Suess, E., Methane-derived authigenic carbonates formed by subduction-induced pore-water expulsion along the Oregon/Washington margin. *GSA Bull*. **98**, 147-156 (1987).

Salmi, M. S., Johnson, H. P. & Harris, R. N. Thermal environment of the Southern Washington region of the Cascadia subduction zone. *J. Geophys. Res. Sol. Earth* **122**, 5852-5870 (2017).

Snavely, P. D., Jr. & Wells, R. E. *Cenozoic Evolution of the Continental Margin of Oregon and Washington.* (*USGS Open File Rep.* **91-441B**, 1991).

### 59. Salton Sea (Onshore California)

Svensen, H., et al. Dynamics of hydrothermal seeps from the Salton Sea Geothermal System (California, USA) constrained by temperature monitoring and time series analysis. *J. Geophys. Res.* **114** (2009).

Tran, A., Rudolph, M. L. & Manga, M. Bubble mobility in mud and magmatic volcanoes. *J. Volcan.. Geotherm. Res.* **294**, 11-24 (2015).

### 60. Costa Rica-Nicaragua Accretionary Wedge (Pacific)

Hensen, C., et al. Fluid expulsion related to mud extrusion off Costa Rica—a window to the subducting slab. *Geology* **32**, 201-204 (2004).

Lauer, R. M., Saffer, D. M. & Harris, R. N. Links between clay transformation and earthquakes along the Costa Rican subduction margin. *Geophys. Res. Lett.* **44**, 7725-7732 (2017).

Moerz, T., et al. Styles and productivity of mud diapirism along the middle American margin, Part I: margin evolution, segmentation, dewatering and mud diapirism, in *Mud Volcanoes, Geodynamics and Seismicity* (eds. Martinelli, G. & Panahi, B.) (Springer, 2005a).

Moerz, T., et al. Styles and productivity of mud diapirism along the middle American margin. Part II: Mound Culebra and Mounds 11 and 12, in *Mud Volcanoes, Geodynamics and Seismicity* (eds. Martinelli, G. & Panahi, B.) (Springer, 2005b).

Shipley, T. H., Stoffa, P. L. & Dean, D. F. Underthrust sediments, fluid migration paths, and mud volcanoes associated with the accretionary wedge off Costa Rica: middle America trench. *J. Geophys. Res.* **95**, 8743-8752 (1990).

Talukder, A. R., et al. Tectonic framework of the mud mounds, associated BSRs and submarine landslides, offshore Nicaragua Pacific margin. *J. Geol. Soc.* **165**, 167-176 (2008).

### 61. Tumaco Basin (Pacific Colombia)

Suárez-Rodríguez, M. A. Geological framework of the Pacific coast sedimentary basins, western Colombia. *Geol. Colom.* **32**, 47-62 (2007).

### 62. Esperanza-Guayaquil Basin (Offshore Ecuador)

Benítez, S. B. Évolution géodynamique de la province côtière sud-équatorienne au Crétacé supérieur-Tertiaire. *Géol. Alp.* **71**, 3-163 (1995).

Cobos Mite, L. E. *Estudio Integrado del Golfo de Guayaquil del Mioceno al Reciente* (Escuela Superior Politécnica del Litoral, Guayaquil, Ecuador, 2010).

Witt C., et al. Development of the Gulf of Guayaquil (Ecuador) during the Quaternary as an effect of the North Andean block tectonic escape. *Tectonics* **25**, TC3017 (2006).

### 63. Talara-Tumbes Basin (Offshore Peru)

Brusset, S., et al. Reappraisal of the tectonic style of the Talara-Tumbes forearc basin: Regional insights for hydrocarbon exploration, in *Petroleum Basins and Hydrocarbon Potential of the Andes of Peru and Bolivia* (eds. Zamora, G., McClay, K. R. & Ramos, V. A.) 323–338 (*AAPG Mem.* **117**, 2018).

Espurt, N., et al. Deciphering the Late Cretaceous-Cenozoic structural evolution of the north Peruvian forearc system. *Tectonics* **37**, 251-282 (2018).

Hermoza, W., et al. Structural styles of the offshore Talara and Tumbes forearc basins, in *XIII Congreso Peruano de Geología* (Sociedad Geológica del Perú, Resúmenes Extendidos, 2006).

Vega, M. M. *Architecture Tectonique et Stratigraphique du Bassin d'Avant-Arc de Tumbes (Nord Pérou): Implications pour l'Exploration des Hydrocarbures* (University of Toulouse III-Paul Sabatier, 2009).

### 64. Chile Forearc (Offshore Chile)

Villar-Muñoz, L., et al. Heat flow in the southern Chile forearc controlled by large-scale tectonic processes. *Geo-Mar. Lett.* **34**, 185-98 (2014).

### 65. Serpentinite Mud Volcanoes (Mariana Forearc)

Asafuah, T. K. & Calvert, A. J. Seismic constraints on the structure of the Fantangisña (Celestial) serpentinite mud volcano in the Mariana subduction zone. *Geophys. J. Int.* **218**, 762-772 (2019).

Fryer, P., et al. Significance of serpentine mud volcanism in convergent margins, in *Ophiolites and Oceanic Crust: New Insights from Field Studies and the Ocean Drilling Program* (eds. Dilek, Y., Moores, E. M., Elthon, D. & Nicolas, A.) (*GSA Spec. Paper* **349**, 2000).

Fryer, P., et al. Site U1497, in *Mariana Convergent Margin and South Chamorro Seamount* (eds. Fryer, P., Wheat, C. G., Williams, T. & Expedition 366 Scientists) (*Proc. Int. Ocean Disc. Prog.* **366**, 2018).

Oakley, A. J., et al. Emplacement, growth, and gravitational deformation of serpentinite seamounts on the Mariana forearc. *Geophys. J. Int.* **170**, 615–634 (2007).

Wheat, C. G., Seewald, J. S. & Takai, K. Fluid transport and reaction processes within a serpentinite mud volcano: South Chamorro Seamount. *Geochim. Cosmochim. Acta* **269**, 413-428 (2020).

*(Supplementary Information 2)*

# 2. Composition and Geomechanical−Test Conditions in Shales

*Contents:*

Table s2 | Composition and and geomechanical−test conditions of the various shales compiled for this study …. *(page 37)*

Table s3 | Ternary diagrams for classification of shales …. *(page 42)*

Figure s2 | Stress paths and stress-strain curves for shales (Supplementary to Fig. 3) …. *(page 44)*

Figure s3 | Stress−strain curves for shales under different confining pressures (Supplementary to Fig. 5a) …. *(page 46)*

Figure s4 | Stress−strain curves for shales under different temperature conditions (Suppl. to Fig. 5b) …. *(page 47)*

Figure s5 | Stress−strain curves for shales depending on orientation of fabric (Supplementary to Fig. 5c) …. *(page 48)*

Figure s6 | Shear strength of London Clay measured using Vane test …. *(page 49)*

Supplementary references for geomechanics tests in shales …. *(page 50)*

**Table s2 |** Composition and geomechanical-test conditions of the various shales compiled for this study.

| Shale Sample | Author | Onshore (On)-Offshore (Off) | Sample No. | Age | Sedimentary conditions |
|---|---|---|---|---|---|
| Chengkou Shale (Lujiaping Fm., Yangtze Basin, China) (12) | Wang, D. et al. (2020) | On | n.a. | Lower Cambrian | marine |
| Alum Shale (Denmark) (13) | Rybacki et al. (2015, 2016) | On | ALM | Cambrian | marine |
| Marcellus Shale (Texas) (14) | Wang, Y. et al. (2020) | On | M_V | Middle Devonian | deep marine |
| Barnett Shale (Texas) (15) | Rybacki et al. (2015, 2016) | On | BAR | Carboniferous | marine |
| – (Allenwood, Pennsylvania, USA) | Villamor Lora et al. (2016) | On | n.a. | | marine |
| Tournemire Shale (Massif Central, France) | Niandou et al. (1997), Masri et al. (2014) | On | n.a. | Lower Jurassic | marine |
| – (Tournemine TRL tunnel, Les Fournials, France) (16) | Bonnelye et al. (2017) | On | n.a. | | marine |
| – (Tournemine TRL tunnel, Les Fournials, France) | Abdi et al. (2015) | On | n.a. | | marine |
| Posidonia Shale (Germany) (immature oil shale; Dotternhausen) (17) | Rybacki et al. (2015, 2016) | On | DOT | Lower Jurassic | deep marine |
| – (immature oil shale; Wickensen) | | On | WIC | | deep marine |
| – (peak oil maturity shale; Harderode) | | On | HAR | | deep marine |
| – (overmature gas shale; Haddessen) | | On | HAD1 | | deep marine |
| Opalinus Clay (18) | Amann et al. (2011) | On | n.a. | Lower-Middle Jurassic | marine |
| Kimmeridge Clay (Westbury Quarry, Wiltshire, UK) (19) | Nygård and Gutierrez (2002), Nygård et al. (2004a,b, 2006) | On | KWC (fissile clay) | Upper Jurassic | marine |
| – (Isle of Purbeck, Dorseth, UK) | | On | KBC (laminated clay) | | marine |
| – (Isle of Purbeck, Dorseth, UK) | Gutierrez et al. (2008) | On | KBC (laminated clay) | | marine |
| Draupne Shale (Ling depression, Central North Sea) (20) | Mondol (2019) | Off | n.a. | Upper Jurassic | marine |
| Offshore Australia | Ewy et al. (2020) | Off | E | Lower Cretaceous | marine |
| Eagle Ford Shale (Texas) (21) | Wang, Y. et al. (2020) | On | EF_V | Upper Cretaceous | marine |
| Qingshankou Fm. (Song-liao Basin, China) | Liu et al. (2020) | On | #15 | Upper Cretaceous | lacustrine |
| Pierre-1 (Great Plains, USA) | Islam et al. (2010), Islam and Skalle (2013) | On | #1 | Upper Cretaceous | marine |
| N Gulf of Mexico | Ewy et al. (2020) | Off | A | Cretaceous-Paleocene | marine |
| Tertiary Shale (North Sea) | Horsrud et al. (1988) and Horsrud (2001) | Off | D and TP2 | Paleocene | marine |
| Wilcox Shale (N Gulf of Mexico) (22) | Ibanez and Kronenberg (1993) | Off | n.a. | Middle Paleocene-Lower Eocene | marine |
| Shahejie Fm. (Bohai Bay Basin, China) | Liu et al. (2020) | On | #5 | Oligocene | lacustrine |
| Boom Clay (Belgium) (23) | Yu et al. (2012, 2018) | On | n.a. | Lower Oligocene | marine |
| Offshore Angola (Cabinda) | Ewy et al. (2020) | Off | F | Upper Oligocene | marine |
| Southern Moravia Clays (Vienna Basin; Brno, Czech Republic) (24) | Feda et al. (1995) | On | SM-B and BRN | Miocene | marine |
| – (Western Bohemia Clays; Vienna Basin, Czech Republic) | | On | WB-AZ and SG | Miocene | lacustrine |
| Offshore Angola (Cabinda) | Ewy et al. (2020) | Off | G | Middle Miocene | marine |
| – | | Off | C | Upper Miocene | marine |
| Mudstone (Kuqa Depression, Tarim Basin, China) | Wang, S. et al. (2020) | On | n.a. | unk. | unk. |

**Table s2** (continuation) ❙ Composition and geomechanical-test conditions of the various shales compiled for this study.

| Shale Sample | Depth (m) (1) | $T_{max}$ (°C) (2) | Bulk density ($\times10^3$ kg $m^{-3}$) | Porosity (%) | Geomechanical testing conditions (3) | | | | |
|---|---|---|---|---|---|---|---|---|---|
| | | | | | P (MPa) | T (°C) | Anisotr | Strain rate ($s^{-1}$) (4) | D |
| Chengkou Shale (Lujiaping Fm., Yangtze Basin, China) (12) | outcrop | ~220 | – | 4.4 | 0-65 | room | paral,perp | 0.1 | u |
| Alum Shale (Denmark) (13) | 17 | ~100-150 | 2.65 | 1.3 | 0.1-400 | 20-400 | paral,perp | $5 \times10^{-4}$-$10^{-6}$ | u |
| Marcellus Shale (Texas) (14) | outcrop | ~180-220 | 2.62-2.67 | 1.41-1.79 | 10.3 | room | paral,perp | $1 \times10^{-6}$ | u |
| Barnett Shale (Texas) (15) | 1150 | ~130-140 | – | 0.6 | 0.1-405 | 20-405 | paral,perp | $5 \times10^{-4}$-$10^{-6}$ | u |
| – (Allenwood, Pennsylvania, USA) | -100 | – | 2.58 | 5.0 | 0-70 | room | perp | $1 \times10^{-5}$ | u |
| Tournemire Shale (Massif Central, France) | Tournemire site | 65 (?) | 2.72 | 8.35 | 5-20 | 20-250 | paral,perp | $1.0 \times10^{-6}$ | d |
| – (Tournemine TRL tunnel, Les Fournials, France) (16) | TRL tunnel (-250) | – | ~2.5 | 16-18 | 2.5-80 | room | 0, 45, 90 | $10^{-5}$ and $10^{-7}$ | u-d |
| – (Tournemine TRL tunnel, Les Fournials, France) | TRL tunnel (-250) | – | 2.55 | 9.52 | 0-10 | room | 0, 30, 45, 90 | $3.76 \times10^{-6}$ | u |
| Posidonia Shale (Germany) (immature oil shale; Dotternhausen) (17) | outcrop | ~40-50 | – | 11 | 0.1-401 | 20-401 | paral,perp | $5 \times10^{-4}$-$6.7 \times10^{-5}$ | u |
| – (immature oil shale; Wickensen) | -30-50 | – | – | 6.5-8.0 | 0.1-402 | 20-402 | paral,perp | $5 \times10^{-4}$-$6.7 \times10^{-5}$ | u |
| – (peak oil maturity shale; Harderode) | -30-50 | – | – | 0.7 | 0.1-403 | 20-403 | paral,perp | $5 \times10^{-4}$-$6.7 \times10^{-5}$ | u |
| – (overmature gas shale; Haddessen) | -30-50 | – | – | 8.3 | 0.1-404 | 20-404 | paral,perp | $5 \times10^{-4}$-$6.7 \times10^{-5}$ | u |
| Opalinus Clay (18) | 200-300 | 30-60 | 2.45 | 17-18 | n.a. | room | perp | $2.7$-$3.4 \times10^{-2}$ | n.a. |
| Kimmeridge Clay (Westbury Quarry, Wiltshire, UK) (19) | outcrop | unk. [500m] | 2.65 | 53 | 1-70 | 95 | perp (?) | $0.6$-$1.1 \times10^{-6}$ | u |
| – (Isle of Purbeck, Dorseth, UK) | outcrop | 80-82 [1.7-2 km] | 2.64 | 22 | 1-70 | 95 | perp (?) | $0.6$-$1.1 \times10^{-6}$ | u |
| – (Isle of Purbeck, Dorseth, UK) | outcrop | 80-82 [1.7-2 km] | 2.64 | 22 | 1-58 | room | perp (?) | – | u |
| Draupne Shale (Ling depression, Central North Sea) (20) | 2575-2584 | 80-90 | 2.24 | 7.8 | 0.5-20 | room | 0, 45, 90 | $6.5 \times10^{-5}$ | u |
| Offshore Australia | 1478 | 75 | 2.34 | 21 | – | room | perp | unk. | u |
| Eagle Ford Shale (Texas) (21) | outcrop | ~430-490 | 2.20-2.21 | 9.7-10.04 | 13.8 | room | paral,perp | $1 \times10^{-6}$ | u |
| Qingshankou Fm. (Song-liao Basin, China) | 2413 | 413 | – | – | 30 | room | perp (?) | unk. | u (?) |
| Pierre-1 (Great Plains, USA) | outcrop | 80 | 2.33 | 23.2 | 17-30 | room | 0, 30, 45, 90 | $0.1$-$0.2 \times10^{-6}$ | u |
| N Gulf of Mexico | 1133 | 66 | 2.17 | 31 | – | room | perp | unk. | u |
| Tertiary Shale (North Sea) | 1870 | ~80 | – | 34 | 2.5-15 | room-80 | paral,perp | unk. | u |
| Wilcox Shale (N Gulf of Mexico) (22) | 3955-3960 | 121 | – | 2.5 | 20-400 | 22-200 | 0, 45, 90 | $2.2\times10^{-7}$ - $2.3\times10^{-4}$ | d |
| Shahejie Fm. (Bohai Bay Basin, China) | 2812 | 425 | – | – | 30 | room | perp (?) | unk. | u (?) |
| Boom Clay (Belgium) (23) | 223 | [< 1.4 km] | 2.01-2.04 | 40 | 2.5-4.7 | 22-80 | perp | $4.4 \times10^{-6}$ | u |
| Offshore Angola (Cabinda) | 1816 | 97 | 2.38 | 19 | – | room | perp | unk. | u |
| Southern Moravia Clays (Vienna Basin; Brno, Czech Republic) (24) | -16-22 | [<550 m] | 2.69-2.72 | 50-62 | 0.05-0.06 | room | perp (?) | unk. | u |
| – (Western Bohemia Clays; Vienna Basin, Czech Republic) | -75-103 | – | 2.72-2.73 | 41 | 0.4-1.2 | room | perp (?) | unk. | u |
| Offshore Angola (Cabinda) | 2098 | 110 | 2.42 | 16 | – | room | perp | unk. | u |
| – | 1261 | 77 | 2.21 | 29 | – | room | perp | unk. | u |
| Mudstone (Kuqa Depression, Tarim Basin, China) | unk. | – | – | – | 0-65 | room | perp | unk. | unk. |

**Table s2** (continuation) **|** Composition and geomechanical-test conditions of the various shales compiled for this study.

| Shale Sample | Whole rock mineralogy (wt %) (5) | | | | | | | Ternary components (10) | | |
|---|---|---|---|---|---|---|---|---|---|---|
| | TOC | Qz | KFs (6) | Pl | Cal (7) | Total Clay (8) | Major clays (9) | Q | CAL | CLAY |
| Chengkou Shale (Lujiaping Fm., Yangtze Basin, China) (12) | 1.8-10.4 (av. 5.6) | 57.2 | | 4.9 | 17.1 | 15.1 | Ilt | 65.9 | 18.1 | 16.0 |
| Alum Shale (Denmark) (13) | 16.5 | 24.6 | 2.1 | | 0.7 | 51.0 | unk. | 34.1 | 0.9 | 65.1 |
| Marcellus Shale (Texas) (14) | unk. | 9.0 | 2.0 | | 82.0 | 7.0 | Ilt, Sme | 11.0 | 82.0 | 7.0 |
| Barnett Shale (Texas) (15) | 13.8 | 21.5 | 6.2 | | 5.2 | 52.1 | unk. | 32.6 | 6.1 | 61.3 |
| – (Allenwood, Pennsylvania, USA) | 2.7 | 29.1 | 4.2 | | 3.8 | 57.6 | Ilt, Chl, Kln | 35.2 | 4.0 | 60.8 |
| Tournemire Shale (Massif Central, France) | unk. | 19.0 | 8.0 | | 15.0 | 55.0 | Ilt, Sme, Kln, Chl | 27.8 | 15.5 | 56.7 |
| – (Tournemine TRL tunnel, Les Fournials, France) (16) | 10 | 15.0 | 1.0 | 9.0 | 15.0 | 60.0 | Ilt, Sme, Kln, Chl | 25.0 | 15.0 | 60.0 |
| – (Tournemine TRL tunnel, Les Fournials, France) | unk. | 19.0 | 8.0 | | 15.0 | 55.0 | Ilt, Sme, Kln, Chl | 27.8 | 15.5 | 56.7 |
| Posidonia Shale (Germany) (immature oil shale; Dotternhausen) (17) | 14.9 | 12.7 | 0.0 | | 41.6 | 18.0 | unk. | 17.6 | 57.5 | 24.9 |
| – (immature oil shale; Wickensen) | 17 | 13.2 | 1.6 | | 33.9 | 25.6 | unk. | 19.9 | 45.6 | 34.5 |
| – (peak oil maturity shale; Harderode) | 10.5 | 16.3 | 2.1 | | 39.6 | 28.4 | unk. | 21.3 | 45.8 | 32.9 |
| – (overmature gas shale; Haddessen) | 10.8 | 13.8 | 4.5 | | 27.5 | 32.6 | unk. | 23.3 | 35.1 | 41.6 |
| Opalinus Clay (18) | 0.8 | 13.7 | 1.0 | 1.0 | 16.5 | 66.0 | Kln, Ilt, Sme, Chl | 16.0 | 16.8 | 67.2 |
| Kimmeridge Clay (Westbury Quarry, Wiltshire, UK) (19) | 7-8 | 25.0 | | | 20.0 | 55.0 | Ilt, Sme, Kln | 25.0 | 20.0 | 55.0 |
| – (Isle of Purbeck, Dorseth, UK) | 7-8 | 30.0 | | | 5.0 | 60.0 | Kln, Ilt, Sme | 31.6 | 5.3 | 63.2 |
| – (Isle of Purbeck, Dorseth, UK) | | | | | | | | | | |
| Draupne Shale (Ling depression, Central North Sea) (20) | 6.8 | 22.4 | 17.8 | | 1.9 | 50.7 | Kln, Ilt, Sme | 43.3 | 2.0 | 54.6 |
| Offshore Australia | unk. | 23.0 | 11.0 | | 0.0 | 65.0 | Ilt, Sme, Kln | 34.3 | 0.0 | 65.7 |
| Eagle Ford Shale (Texas) (21) | 5-10 | 22.0 | | | 64.0 | 14.0 | Ilt, Sme | 22.0 | 64.0 | 14.0 |
| Qingshankou Fm. (Song-liao Basin, China) | 3.60 | 43.0 | | 15.0 | 4.0 | 39.0 | unk. | 57.4 | 4.0 | 38.6 |
| Pierre-1 (Great Plains, USA) | 7.16 | 20.1 | 0.7 | 15.7 | 4.3 | 57.4 | Ilt, Sme | 37.2 | 4.4 | 58.5 |
| N Gulf of Mexico | unk. | 27.0 | 9.6 | | 5.0 | 59.0 | Ilt, Sme, Kln | 36.4 | 5.0 | 58.6 |
| Tertiary Shale (North Sea) | unk. | unk. | unk. | | unk. | 56.0 | Sme, Kln, Ilt | – | – | – |
| Wilcox Shale (N Gulf of Mexico) (22) | unk. | 37.0 | 4.0 | | 4.0 | 55.0 | Ilt, Chl, Kln | 41.0 | 4.0 | 55.0 |
| Shahejie Fm. (Bohai Bay Basin, China) | 0.08 | 18.0 | 2.0 | 7.0 | 19.0 | 19.0 | unk. | 41.5 | 29.2 | 29.2 |
| Boom Clay (Belgium) (23) | 1-5 | 15.0 | 4.0 | | 6.0 | 75.0 | Ilt, Sme, Chl | 19.0 | 6.0 | 75.0 |
| Offshore Angola (Cabinda) | unk. | 13.0 | 5.0 | | 4.0 | 76.0 | Ilt, Sme, Kln | 18.4 | 4.1 | 77.6 |
| Southern Moravia Clays (Vienna Basin; Brno, Czech Republic) (24) | unk. | 46.0 | | | | 51.0 | Ilt, Kln | 47.4 | 0.0 | 52.6 |
| – (Western Bohemia Clays; Vienna Basin, Czech Republic) | | | | | | | | | | |
| Offshore Angola (Cabinda) | unk. | 14.0 | 5.0 | | 1.9 | 76.0 | Ilt, Sme, Kln | 19.6 | 2.0 | 78.4 |
| – | unk. | 16.0 | 3.2 | | 2.0 | 78.0 | Ilt, Sme, Kln | 19.4 | 2.0 | 78.6 |
| Mudstone (Kuqa Depression, Tarim Basin, China) | unk. | unk. | unk. | | unk. | unk. | unk. | n.a. | n.a. | n.a. |

**Table s2** (continuation) | Composition and geomechanical-test conditions of the various shales compiled for this study.

| Shale Sample | Shale type (11) |
|---|---|
| Chengkou Shale (Lujiaping Fm., Yangtze Basin, China) (12) | Siliceous shale, Sarl, Black shale |
| Alum Shale (Denmark) (13) | Argillaceous shale, Argillaceous Sarl, Black shale |
| Marcellus Shale (Texas) (14) | Calcareous shale, Carl, Black shale |
| Barnett Shale (Texas) (15) | Argillaceous shale, Argillaceous Sarl, Black shale |
| – (Allenwood, Pennsylvania, USA) | |
| Tournemire Shale (Massif Central, France) | Argillaceous shale, Argillaceous Sarl, Black shale |
| – (Tournemine TRL tunnel, Les Fournials, France) (16) | |
| – (Tournemine TRL tunnel, Les Fournials, France) | |
| Posidonia Shale (Germany) (immature oil shale; Dotternhausen) (17) | Calcareous shale, Carl, Black shale |
| – (immature oil shale; Wickensen) | Mixed shale, Carl, Black shale |
| – (peak oil maturity shale; Harderode) | Mixed shale, Carl, Black shale |
| – (overmature gas shale; Haddessen) | Mixed shale, Carl, Black shale |
| Opalinus Clay (18) | Argillaceous shale, Argillaceous Carl, low-TOC Black shale |
| Kimmeridge Clay (Westbury Quarry, Wiltshire, UK) (19) | Argillaceous shale, Argillaceous Sarl, Black shale |
| – (Isle of Purbeck, Dorseth, UK) | Argillaceous shale, Argillaceous Sarl, Black shale |
| – (Isle of Purbeck, Dorseth, UK) | |
| Draupne Shale (Ling depression, Central North Sea) (20) | Mixed shale, Argillaceous Sarl, n.a. |
| Offshore Australia | Argillaceous shale, Argillaceous Sarl, n.a. |
| Eagle Ford Shale (Texas) (21) | Calcareous shale, Carl, Black shale |
| Qingshankou Fm. (Song-liao Basin, China) | Siliceous shale, Sarl, Black shale |
| Pierre-1 (Great Plains, USA) | Argillaceous shale, Argillaceous Sarl, Black shale |
| N Gulf of Mexico | Mixed shale, Argillaceous Sarl, n.a. |
| Tertiary Shale (North Sea) | unk. |
| Wilcox Shale (N Gulf of Mexico) (22) | Argillaceous shale, Argillaceous Sarl, n.a. |
| Shahejie Fm. (Bohai Bay Basin, China) | Mixed shale, Sarl, Black shale |
| Boom Clay (Belgium) (23) | Argillaceous shale, Bio-siliceous Tarl, Black shale |
| Offshore Angola (Cabinda) | Argillaceous shale, Bio-siliceous Tarl, n.a. |
| Southern Moravia Clays (Vienna Basin; Brno, Czech Republic) (24) | Argillaceous shale, Argillaceous Sarl, n.a. |
| – (Western Bohemia Clays; Vienna Basin, Czech Republic) | |
| Offshore Angola (Cabinda) | Argillaceous shale, Bio-siliceous Tarl, n.a. |
| – | Argillaceous shale, Bio-siliceous Tarl, n.a. |
| Mudstone (Kuqa Depression, Tarim Basin, China) | unk. |

**Table s2** (continuation) **|** Composition and geomechanical−test conditions of the various shales compiled for this study.

**Notes and abbreviations:**

(1) Sample depth, meters below seafloor (when sample comes from borehole) or below surface (in samples cored in onshore wells). Negative values are depths below surface in onshore wells or tunnels.

(2) Maximum temperature achieved by sample or paleo-temperatures (coming from in-situ downhole measurements or according to estimate based on maximum hydrocarbon generation and/or vitrinite reflectance). Maximum burial depths in brackets.

(3) Abbreviations for geomechanical testing conditions (according to triaxial deformation experiments): confining pressure (P, in MPa); temperature (T, in °C); variable orientation of fabric (layering and stratification) with respect to main stresses (Anisotr) (angle between $\sigma_1$ and fabric in degrees, perp= sample compressed perpendicular to fabric, paral= idem. parallel to fabric) constant or variable strain rates (values in $s^{-1}$); drained (d) and/or undrained (u) conditions (D).

(4) When values not provided by authors, strain rate calculated using loading rate of experiment and dimensions of sample (e.g., rate in mm/s divided by length of sample cylinder in mm).

(5) According to XRD determinations. Mineral abbreviations following Kretz (1983) and Whitney and Evans (2010): Cal (calcite), Chl (chlorite), Ilt (illite), Kfs (K-feldspar), Kln (kaolinite), Pl (plagioclase), Qz (quartz), Sme (smectite), TOC (total organic content).

(6) Including plagioclase.

(7) Including other carbonate minerals (e.g., dolomite, siderite, ankerite, aragonite).

(8) Including all clay minerals (e.g., smectite, illite, kaolinite, glauconite) and chlorite.

(9) Main clay minerals in sample, ordered in decreasing abundance.

(10) The Q component includes quartz, K-feldspar, and plagioclase.

(11) According to ternary classifications (Table s3) of Nance and Rowe (2015) [first classification name] and Milliken (2014) [second classification name]. Organic-matter content used to classify shale according to Hay et al. (1984) and Trabucho-Alexandre (2015) [third classification name] (black shale= 0.5-20% TOC; oil shale> 20% TOC).

Limit between black and organic shales also established by Cook and Sherwood (1991) at 10% TOC. In Milliken's classification, we assumed all clay minerals part of *Tarl* component.

More detailed classification of Gamero-Diaz et al. (2012, 2013), which also uses composition of shales, not used in this study (Table s3).

For grain−size classification, we followed scheme of Wentworth (1922) modified by Friedman and Sanders (1978): i.e., clay size (< 2μm), silt size (2−62.5 μm), and sand size (62.5 μm−2 mm).

(12) Approximate maximum burial temperatures taken from general study in basin of Tan et al. (2013).

(13) With additional information from Lecomte et al. (2017) and Schovsbo et al. (2018).

(14) Maximum burial temperatures according to Stolper et al. (2014).

(15) Maximum burial temperatures according to Green et al. (2020).

(16) Composition of Tournemire Shale (using upper Toarcian clay member) according to Tremosa et al. (2012). Porosity values from Zhang et al. (2019).

(17) Maximum burial temperatures according to Hofmann et al. (2001).

(18) Petrophysical properties and other characteristics of Opalinus Clay (shaly−facies member) at Monte Terri Rock Laboratory (Switzerland), complemented with information provided in the project website (https://www.mont-terri.ch/en/homepage.html), Bossart and Thury (2008), Joseph et al. (2013), and Schuster et al. (2021). Experimental conditions correspond to uniaxial tests.

**Table s2** (continuation) ǀ Composition and geomechanical−test conditions of the various shales compiled for this study.

**Notes and abbreviations (cont.):**

(19) With additional information from Scotchman (1991). Paleo-temperature estimate according to Obrador-Prats et al. (2019). Maximum burial depth conditions included in column of paleo-temperatures (using data in Nygård and Gutierrez, 2002).

(20) Maximum paleo-temperatures according to study by Baig et al. (2019), using vitrinite reflectance, borehole temperature measurements, and back-stripping analysis.

(21) Maximum temperatures estimated in core samples by Zhang et al. (2017). Clay mineralogy according to Sone and Zoback (2013a, b). Average TOC content is taken from these authors, using their determinations for total volume of kerogen.

(22) Information about composition of this sample does not differentiate between feldspars and calcite. Organic matter content not included in the study.

(23) With additional information from Wiseall et al. (2015). Burial depths in maximum paleo-temperature column taken from these authors.

(24) Composition and geological information of these claystones are from Herbstová and Herle (2009), although the studied localities sampled by Feda et al. (1995) could be different.

n.a.= not applicable; unk. (and −) = unknown conditions or value; (?)= probable value

**Table s3 |** Ternary diagrams for classification of shales.

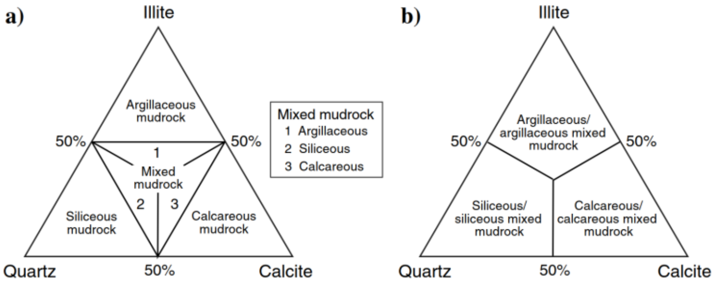


Nance and Rowe (2015)

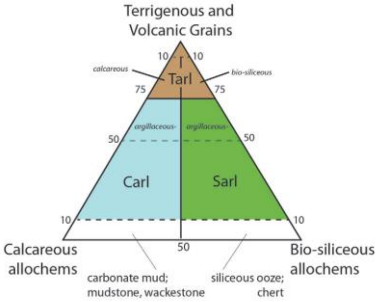


Milliken (2014)

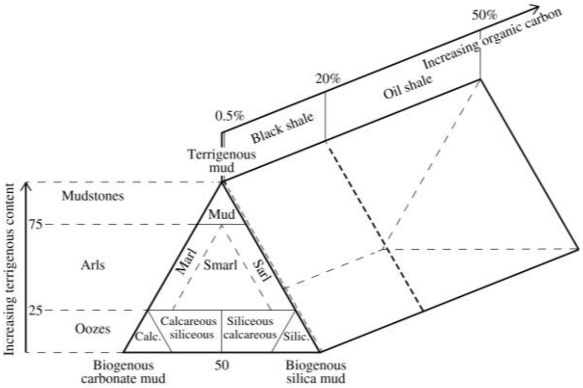


Hay et al. (1984) and Trabucho-Alexandre (2015)

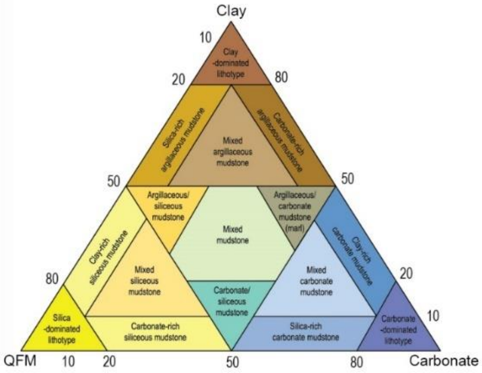


Gamero-Diaz et al. (2012, 2013)

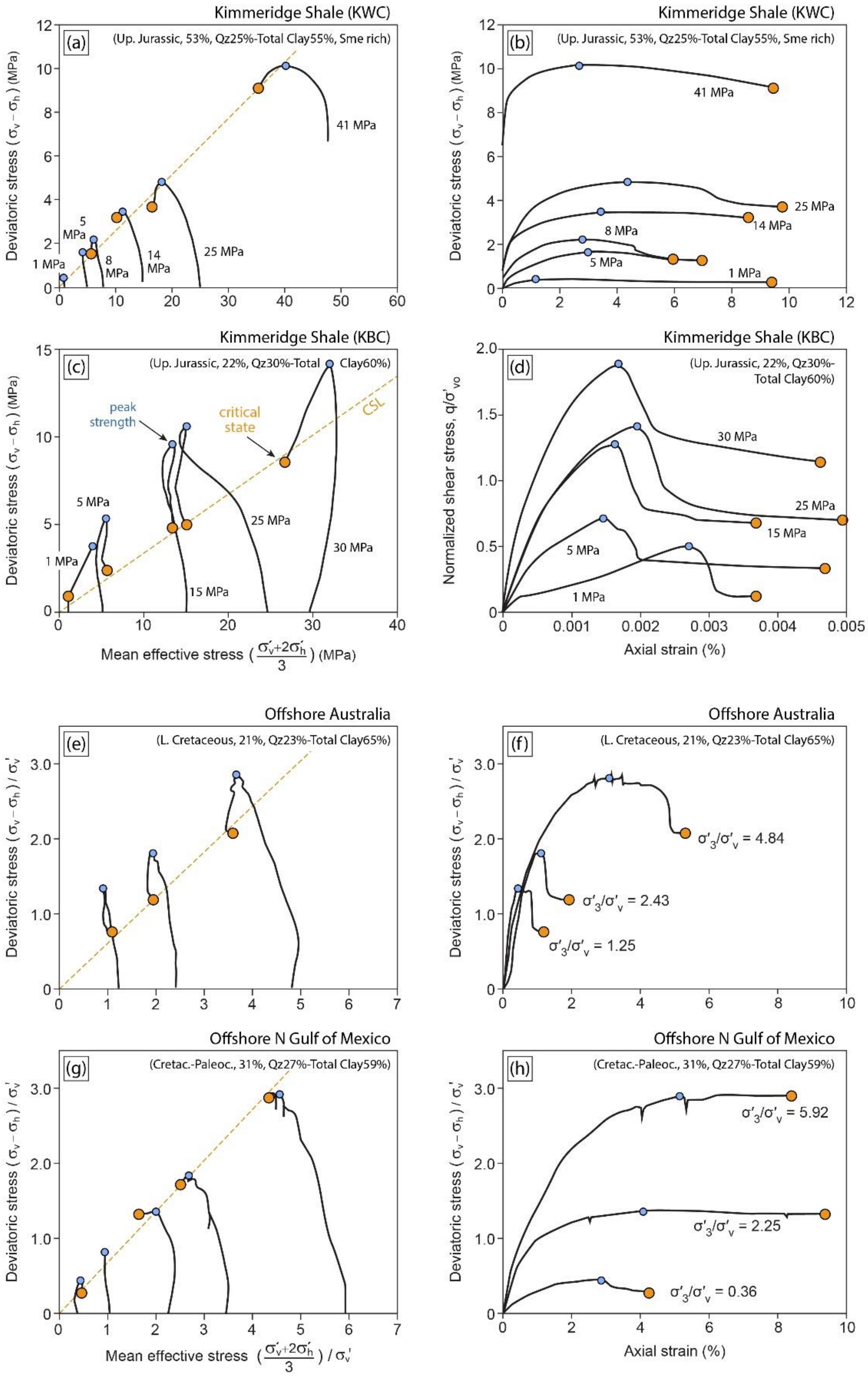


**Fig. s2 | Stress paths and stress-strain curves for shales** (supplementary to Fig. 3). (Complete legend in pages 45−46.)

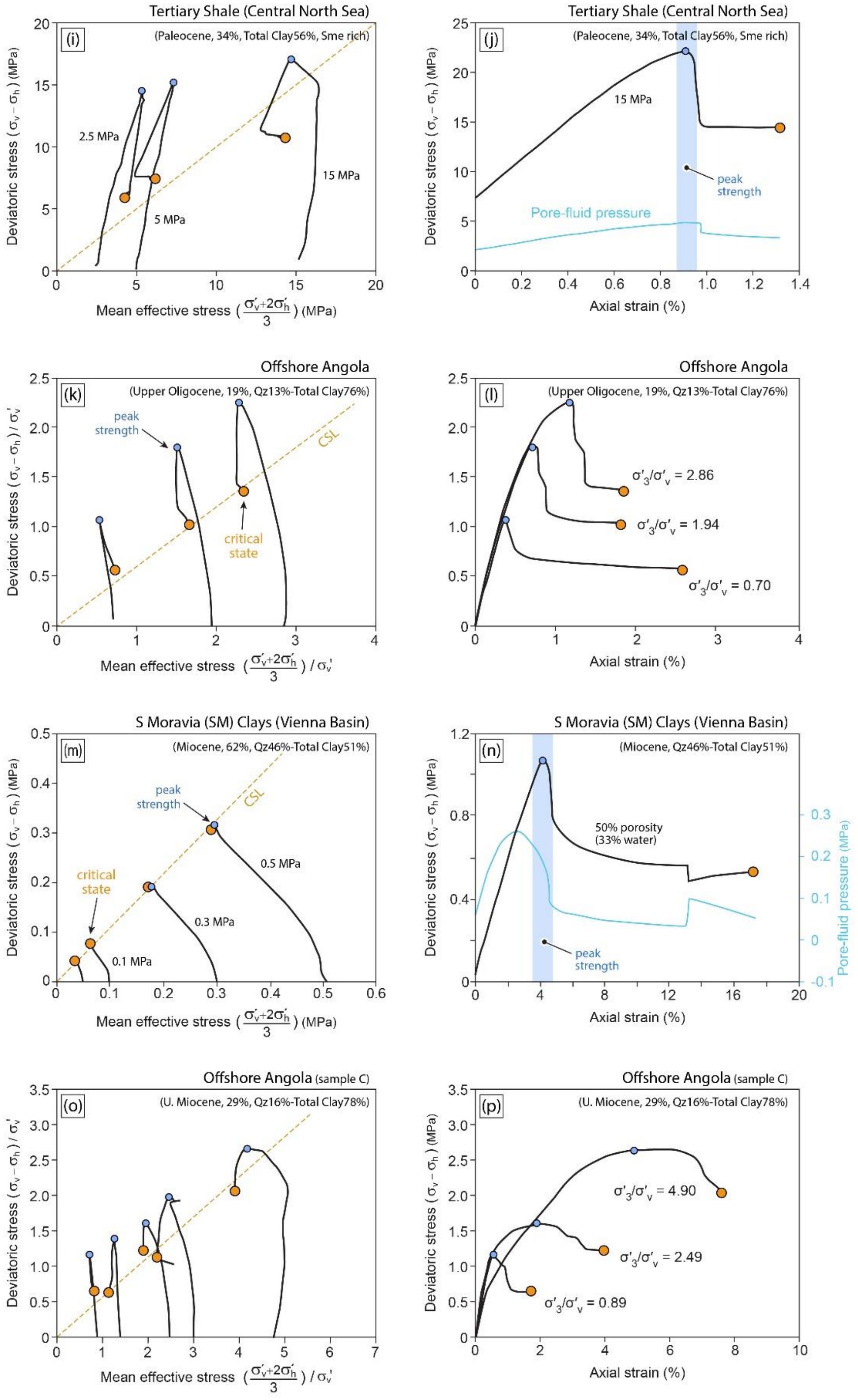


**Fig. s2** (continuation) **| Stress paths and stress-strain curves for shales** (supplementary to Fig. 3). Stress paths always plotted in p′-q diagrams (mean effective stress vs. deviatoric stress, in MPa). Stress-strain curves represented in axial strain (in %) vs. deviatoric stress (in MPa). **(a-b)** Kimmeridge Clay (UK;

low-buried sample, Upper Jurassic) (Nygård and Gutierrez, 2002; Nygård et al., 2004a, b, 2006; Gutierrez et al., 2008). **(c-d)** Kimmeridge Clay (UK; high-buried sample, Upper Jurassic) (Nygård and Gutierrez, 2002; Nygård et al., 2004a, b, 2006; Gutierrez et al., 2008). **(e-f)** Offshore Australia (Lower Cretaceous) (Ewy et al., 2020). **(g-h)** Offshore N Gulf of Mexico (Cretaceous-to-Paleocene) (Ewy et al., 2020). **(i-j)** Tertiary Shale (Central North Sea; Paleocene) (Horsrud et al., 1988; Horsrud, 2001). **(k-l)** Offshore Angola (Ewy et al., 2020). **(m-n)** Southern Moravia marine clays (Czech Republic; Miocene) (Feda et al., 1995). **(o-p)** Offshore Angola (Upper Miocene) (Ewy et al., 2020). Samples ordered by age. Age, porosity (in %), mineral abundance (in %) of quartz (Qz) and total clay fraction included for reference. Details of experimental test conditions and sample composition provided in Table s2. CSL: inferred position of critical state line. Curves in blue show pore-fluid pressure.

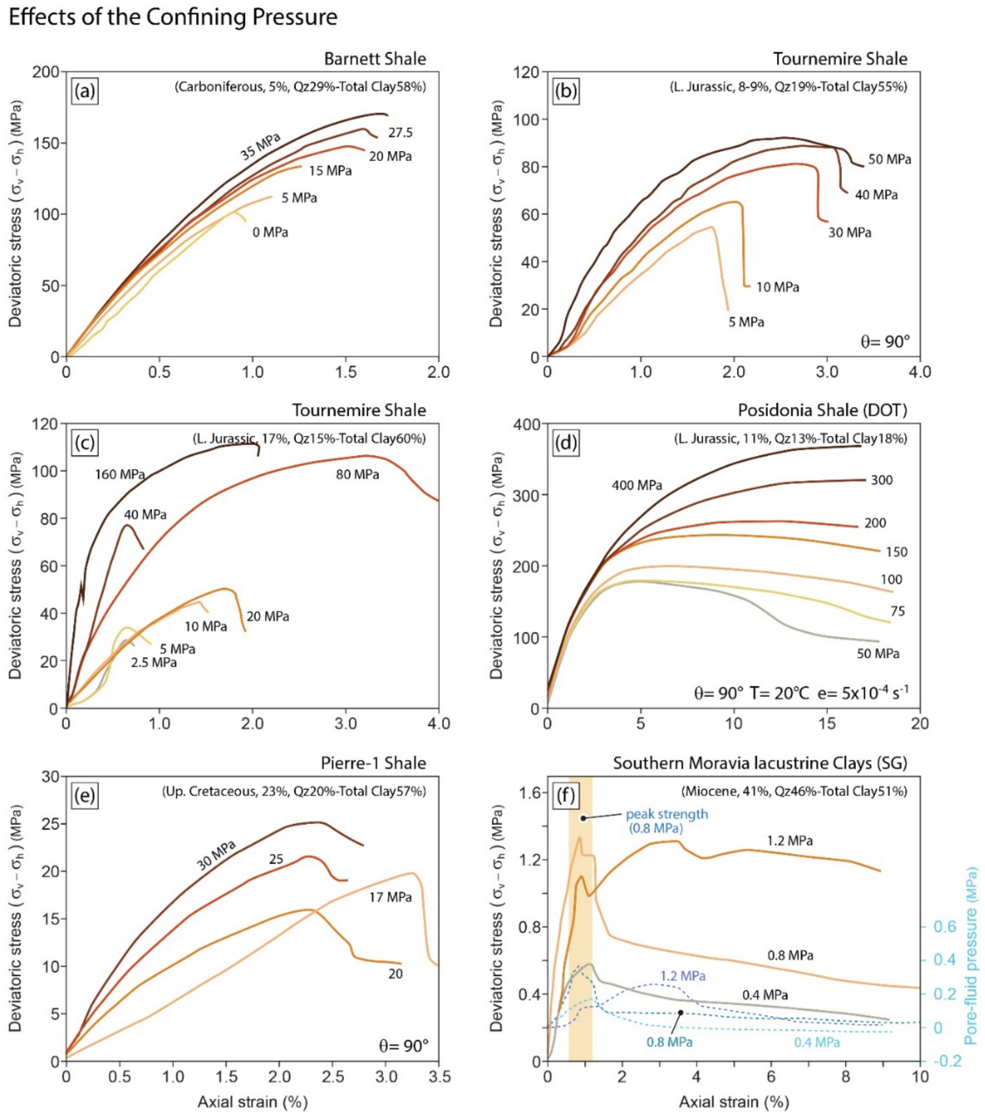


**Fig. s3 | Stress-strain curves for shales under different confining pressures** (supplementary to Fig. 5a). Stress-strain curves represented in axial strain (in %) vs. deviatoric stress (in MPa). **(a)** Barnett Shale (USA, Carboniferous) (Villamor Lora et al., 2016). **(b)** Tournemire Shale (France, Lower Jurassic) (Niandou et al., 1997). **(c)** Tournemire Shale (France, Lower Jurassic) (Bonnelye et al., 2017). **(d)** Posidonia Shale (Germany, DOT sample, Lower Jurassic) (Rybacki et al., 2015, 2016). **(e)** Pierre-1 Shale (USA, Upper Cretaceous) (Islam et al., 2010; Islam and Skalle, 2013). **(f)** Southern Moravia Clays

(Czech Republic, Miocene) (Feda et al., 1995). Samples ordered by age. Age, porosity (in %), mineral abundance (in %) of quartz (Qz) and total clay fraction included for reference. Details of experimental test conditions and sample composition provided in Table s2. Discontinuous curves in blue show pore-fluid pressure.

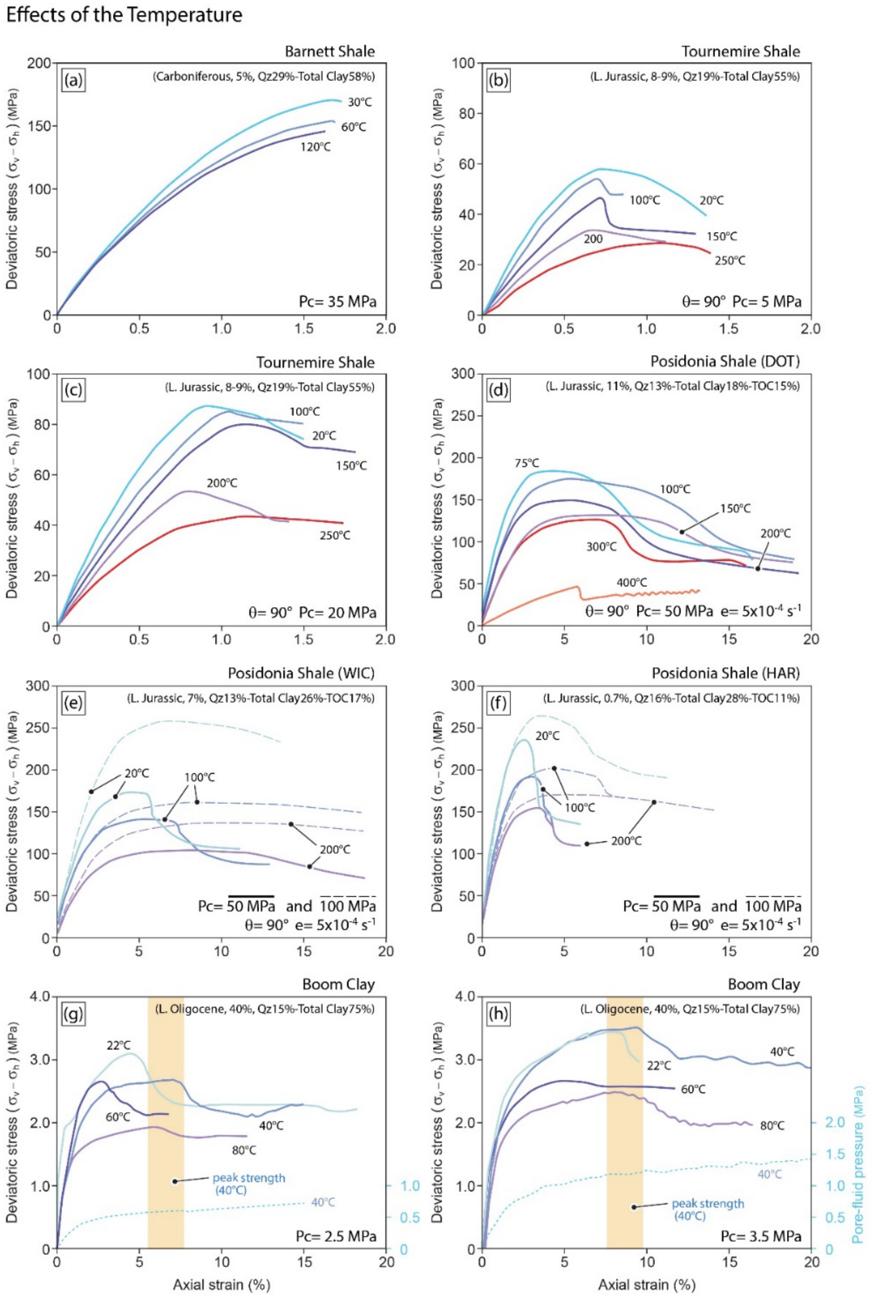


**Fig. s4 | Stress-strain curves for shales under different temperature conditions** (supplementary to Fig. 5b). Stress-strain curves represented in axial strain (in %) vs. deviatoric stress (in MPa). **(a)** Barnett Shale (USA, Carboniferous) (Villamor Lora et al., 2016). **(b-c)** Tournemire Shale (France, Lower Jurassic) (Masri et al., 2014). **(d-f)** Posidonia Shale (Germany, Lower Jurassic) (Rybacki et al., 2015, 2016). These samples are immature oil shales (DOT and WIC) and peak-oil maturity shales (HAR). **(g-h)** Boom Clay (Belgium, Lower Oligocene) (Yu et al., 2012, 2018). Samples ordered by age. Age, porosity (in %) and mineral abundance (in %) of quartz (Qz) and total clay fraction included for

reference. Details of the experiment test conditions and sample composition are provided in Table s2. Dotted curves in blue show pore-fluid pressure.

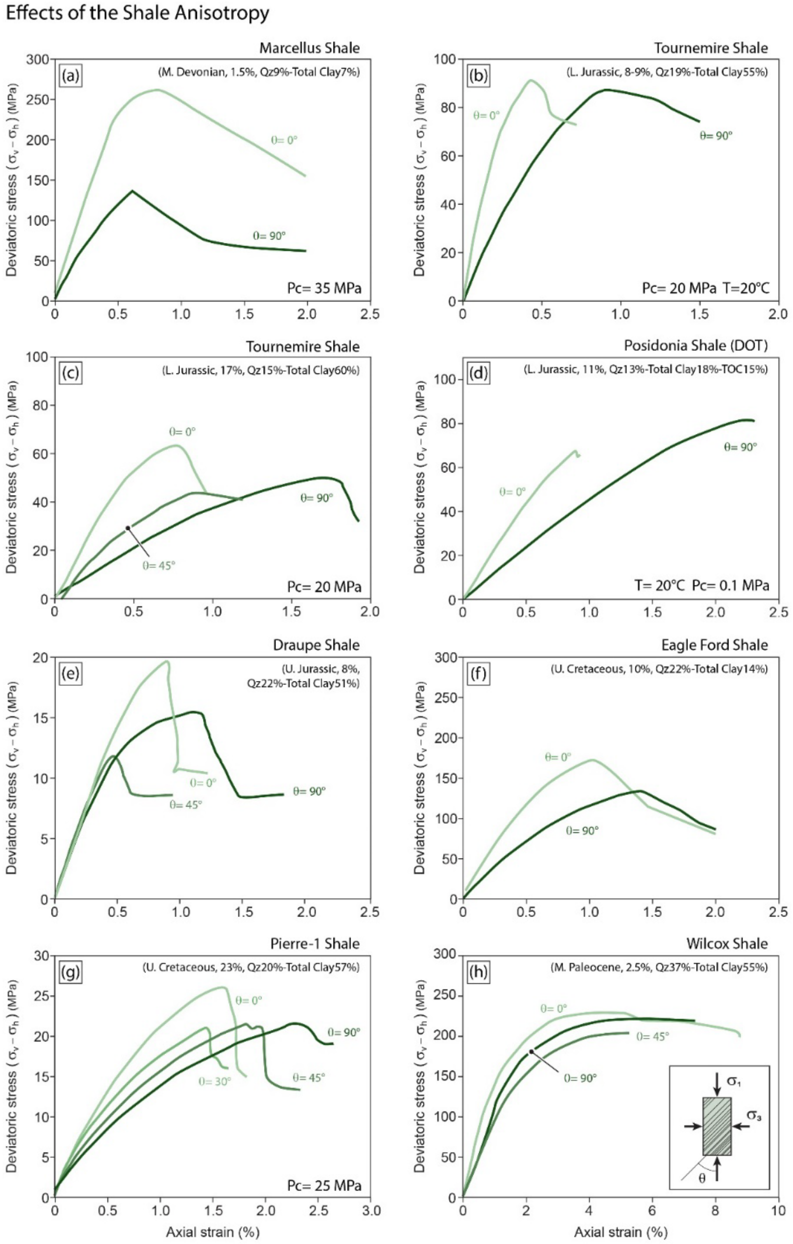


**Fig. s5 | Stress-strain curves for shales depending on orientation of fabric** (supplementary to Fig. 5c). Stress-strain curves are represented in axial strain (in %) vs. deviatoric stress (in MPa). **(a)** Marcellus Shale (USA, Middle Devonian) (Wang et al., 2020). **(b)** Tournemire Shale (France, Lower Jurassic) (Masri et al., 2014). **(c)** Tournemire Shale (France, Lower Jurassic) (Bonnelye et al., 2017). **(d)** Posidonia Shale (Germany, DOT; Lower Jurassic) (Rybacki et al. 2015, 2016). **(e)** Draupe Shale (Central North Sea, Norway; Upper Jurassic) (Mondol, 2019). **(f)** Eagle Ford Shale (USA, Upper Cretaceous) (Wang et al., 2020). **(g)** Pierre-1 Shale (USA, Upper Cretaceous) (Islam et al., 2010; Islam and Skalle, 2013). **(h)** Wilcox Shale (SN Gulf of Mexico, Middle Paleocene-Lower Eocene) (Ibanez and Kronenberg, 1993). Samples ordered by age. Age, porosity (in %), and mineral abundance (in %) of

quartz (Qz) and the total clay fraction included for reference. Details of experiment test conditions and sample composition provided in Table s2. Inset in h illustrates criteria used to define shale anisotropy in experiments as angle (θ) formed between shale fabric (layering or stratification) and main compression.

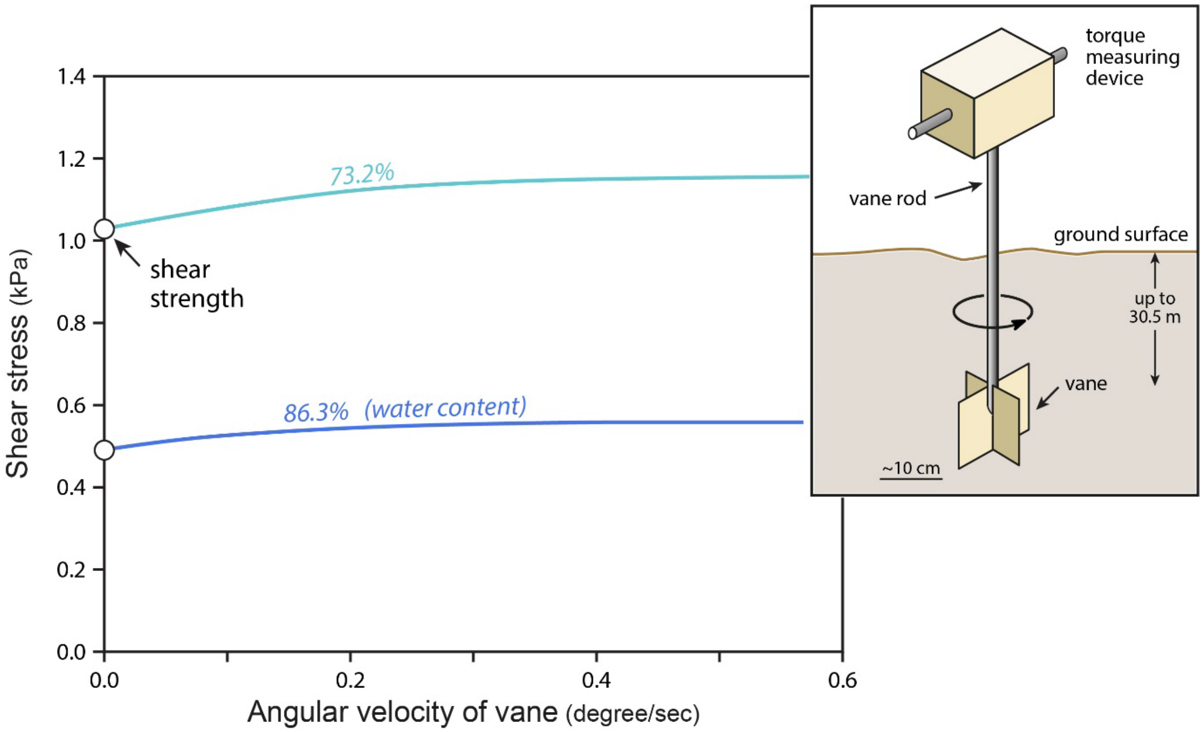


**Fig. s6 | Shear strength of London clay measured using Vane test.** In this test, vertical blades inserted into ground and spun at certain angular velocity, creating cylindrical shear surface (inset schematizes vane apparatus; Skempton, 1948). Soil at this surface undergoes large shear strains and reaches critical state. Measured torque thus represents the critical-state shear strength of soil (Skempton and Northey, 1952). Measurements indicate strength increases slightly with angular velocity (shear strain) rate. Water content (in %), a proxy for porosity, is ratio between weight of pore water to weight of grains in a given volume of soil. Measurements also indicate that soil strength decreases with increase in water content (porosity). Results document that sample behaves like a Herschel-Bulkley fluid (a generalized model for a non-Newtonian fluid). The Herschel-Bulkley equation is:

$$\tau = \tau_0 + K\dot{\gamma}^n \qquad (1)$$

where $\tau$ = shear stress, $\tau_0$ = yield stress, $\dot{\gamma}$ = shear rate, and material constants $K$ and n = consistency index and flow index, respectively (Herschel and Bulkley, 1926).

## Supplementary references for geomechanical tests in shales

References with geomechanical tests in various shales are listed. Characteristics of the shales and experimental studies compiled in this study are summarized in Tables s2 and s3 and in Figures s2 through s6. The mechanical behavior of the different shales is shown in p′-q plots (mean effective stress vs. deviatoric stress) (Fig. s2) and in various strain-stress diagrams according to variable values of confining pressures (Fig. s3), temperatures (Fig. s4), and orientation of fabric (stratification) of the shales with respect to main stresses (Fig. s5). The mechanical behavior of a sample at critical-state conditions is illustrated using results from the Vane test in the London Clay (Fig. s6).

Abdi, H. et al. Laboratory investigation on the mechanical behaviour of Tournemire argillite. *Can. Geotech. J.* **52**, 268–282 (2015).

Amann, F., et al. Experimental study of the brittle behavior of clay shale in rapid unconfined compression. *Rock Mech. Rock Eng.* **44**, 415-430 (2011).

Baig, I., Faleide, J. I., Mondol, N. H. & Jahren, J. Burial and exhumation history controls on shale compaction and thermal maturity along the Norwegian North Sea basin margin areas. *Mar. Petr. Geol.* **104**, 61-85 (2019).

Bonnelye, A., et al. Strength anisotropy of shales deformed under uppermost crustal conditions. *J. Geophys. Res. Sol. Earth* **122**, 110-129 (2017).

Bossart, P. & Thury, M. *Mont Terri Rock Laboratory: Project, Programme 1996 to 2007, and Results* (Wabern: Reports of Swiss Geological Survey **3**, 2008).

Cook, A. C. & Sherwood, N. R. Classification of oil shales, coals and other organic-rich rocks. *Org. Geochem.* **17**, 211-222 (1991).

Ewy, R., Dirkzwager, J. & Bovberg, C. Claystone porosity and mechanical behavior vs. geologic burial stress. *Mar. Petr. Geol.* **121**, 104563 (2020).

Feda, J., Boháč, J. & Herle, I. Shear resistance of fissured Neogene clays. *Eng. Geol.* **39**, 171-184 (1995).

Friedman, G. M. & Sanders, J. E. *Principles of Sedimentology* (Freeman, New York, 1978).

Gamero-Diaz, H., Miller, C. & Lewis, R. Core: a classification scheme for organic mudstones based on bulk mineralogy. *AAPG Search & Disc.* **40951** (2012).

Gamero-Diaz, H., Miller, C., Lewis, R. & Contreras Fuentes, C. Evaluating the impact of mineralogy on reservoir quality and completion quality of organic shale plays. *AAPG Search & Disc.* **41221** (2013).

Green, H., et al. Evaluation of shale source rocks and clay mineral diagenesis in the Permian Basin, USA: Inferences on basin thermal maturity and source rock potential. *Geosciences* **10**, 381 (2020).

Gutierrez, M., Nygård, R., Høeg, K. & Berre, T. Normalized undrained shear strength of clay shales. *Eng. Geol.* **99**, 31-39 (2008).

Hay, W.W., et al. Initial Reports of the Deep-Sea Drilling Project (eds. Hay, W.W., et al.) (*U.S. Gov. Printing Office* **75**, 3-25, 1984).

Herbstová, V. & Herle, I. Structure transitions of clay fills in North-Western Bohemia. *Eng. Geol.* **104**, 157-166 (2009).

Herschel, W. H. & Bulkley, R. Konsistenzmessungen von Gummi-Benzollösungen. *Kolloid Z.* **39**, 291-300 (1926).

Hofmann, P., Leythaeuser, D. & Schwark, L. Organic matter from the Bunte Breccia of the Ries Crater, southern Germany: Investigating possible thermal effects of the impact. *Planet. Space Sci.* **49**, 845-851 (2001).

Horsrud, P. Estimating mechanical properties of shale from empirical correlations. *SPE Drill. Complet.* **16**, 68–73 (2001).

Horsrud, P., Sønstebø, E. F. & Bøe, R. Mechanical and petrophysical properties of North Sea shales. *Int. J. Rock Mech. Min. Sci.* **35**, 1009–1020 (1998).

Ibanez, W. D. & Kronenberg, A. K. Experimental deformation of shale: Mechanical properties and microstructural indicators of mechanisms. *Int. J. Rock Mech. Min. Sci. Geomech. Abs.* **30**, 723-734 (1993).

Islam, M. A. & Skalle, P, An Experimental investigation of shale mechanical properties through drained and undrained test mechanisms. *Rock Mech. Rock Eng.* **46**, 1391-1413 (2013).

Islam, M. A., Skalle, P. & Al-Ajmi, A. M. Stability analysis in shale through deviated boreholes using the Mohr and Mogi-Coulomb failure criteria, in *44th U.S. Rock Mechanics Symposium and 5th U.S.–Canada Rock Mechanics Symposium* (**Paper ARMA10-432**, 2010).

Joseph, C., et al. Diffusion of U(VI) in Opalinus Clay: Influence of temperature and humic acid. *Geochim. Cosmochim. Acta* **109**, 74-89 (2013).

Kretz, R. Symbols of rock-forming minerals. *Amer. Mineral.* **68**, 277-279 (1983).

Lecomte, A., et al. Uranium mineralization in the Alum Shale Formation (Sweden): Evolution of a U-rich marine black shale from sedimentation to metamorphism. *Ore Geol. Rev.* **88**, 71-98 (2017).

Liu, B., et al. Mechanical characteristics and factors controlling brittleness of organic-rich continental shales. *J. Petr. Sci. Eng*. **194**, 107464 (2020).

Masri, M., Sibai, M., Shao, J. F. & Mainguy, M. Experimental investigation of the effect of temperature on the mechanical behavior of Tournemire shale. *Int. J. Rock Mech. Min. Sci.* **70**, 185-191 (2014).

Milliken, K. A compositional classification for grain assemblages in fine-grained sediments and sedimentary rocks. *J. Sed. Res.* **84**, 1185-1199 (2014).

Mondol, N. H. Geomechanical and seismic behaviors of Draupne Shale: A case study from the central North Sea, in *81st EAGE Conference & Exhibition* (**Paper Th_R10_08**, 2019).

Nance, H. S. & Rowe, H. Eustatic controls on stratigraphy, chemostratigraphy, and water mass evolution preserved in a Lower Permian mudrock succession, Delaware Basin, west Texas, USA. *Interpretation* **3**, SH11-SH25 (2015).

Niandou H., Shao J. F., Henry, J. P. & Fourmaintraux, D. Laboratory investigation of the mechanical behavior of Tournemire shale. *Int. J. Rock Mech. Min. Sci.* **34**, 3-16 (1997).

Nygard, R. & Gutierrez, M. Undrained shear behaviour of some UK mudrocks explained by petrology. *J. Can. Petr. Tech.* **41**, 37-46 (2002).

Nygård, R., Gutierrez, M., Bratli, R. F. & Høeg, K. Brittle–ductile transition, shear failure and leakage in shales and mudrocks. *Mar. Petr. Geol.* **23**, 201-212 (2006).

Nygård, R., Gutierrez, M., Gautam, R. & Høeg, K. Compaction behavior of argillaceous sediments as function of diagenesis. *Mar. Petr. Geol.* **21**, 349-362 (2004a).

Nygård, R., Gutierrez, M., Høeg, K. & Bjørlykke, K. Influence of burial history on microstructure and compaction behaviour of Kimmeridge clay. *Petr. Geosci.* **10**, 259-270 (2004b).

Obradors-Prats, J., Rouainia, M., Aplin, A. C. & Crook, A. J. L. A diagenesis model for geomechanical simulations: formulation and implications for pore pressure and development of geological structures. *J. Geophys. Res. Sol. Earth* **124**, 4452-4472 (2019).

Rybacki, E., et al. What controls the mechanical properties of shale rocks? — Part I: strength and Young's modulus. *J. Petr. Sci. Eng.* **135**, 702-722 (2015).

Rybacki, E., Meier, T. & Dresen, G. What controls the mechanical properties of shale rocks? — Part II: brittleness. *J. Petr. Sci. Eng.* **144**, 39–58 (2016).

Schovsbo, N. H., Nielsen, A. T., Harstad, A. O. & Bruton, D. L. Stratigraphy and geochemical composition of the Cambrian Alum Shale Formation in the Porsgrunn core, Skien–Langesund district, southern Norway. *Bull. Geol. Soc. Denmark* **66**, 1-20 (2018).

Schuster, V., et al. Experimental deformation of Opalinus Clay at elevated temperature and pressure conditions: Mechanical properties and the influence of rock fabric. *Rock Mech. Rock Eng.* **54**, 4009–4039.

Scotchman, I. C. Kerogen facies and maturity of the Kimmeridge Clay Formation in southern and eastern England. *Mar. Petr. Geol.* **8**, 278-295 (1991).

Skempton, A. W. Vane tests in the alluvial plain of River Forth near Grangemouth. *Géotechnique* **1**, 111-124 (1948).

Skempton, A. W. & Northey, R. D. The sensitivity of clays. *Géotechnique* **3**, 30-53 (1952).

Sone, H. & Zoback, M. D. Mechanical properties of shale-gas reservoir rocks — Part 1: static and dynamic elastic properties and anisotropy. *Geophysics* **78**, D381–D392 (2013a).

Sone, H. & Zoback, M. D. Mechanical properties of shale-gas reservoir rocks — Part 2: ductile creep, brittle strength, and their relation to the elastic modulus. *Geophysics* **78**, D393–D402 (2013b).

Stolper, D. A., et al. Formation temperatures of thermogenic and biogenic methane. *Science* **344**, 1500-1503 (2014).

Tan, J., et al. Physical properties of petroleum formed during maturation of Lower Cambrian shale in the upper Yangtze Platform, South China, as inferred from PhaseKinetics modelling. *Mar. Petr. Geol.* **48**, 47-56 (2013).

Trabucho-Alexandre, J. Organic matter-rich shale depositional environments in *Fundamentals of Gas Shale Reservoirs* (ed. Rezaee, R.) (Wiley & Sons, 2015).

Tremosa, J., et al. Geochemical characterization and modelling of the Toarcian/Domerian porewater at the Tournemire underground research laboratory. *Appl. Geochem.* **27**, 1417-1431 (2012).

Villamor Lora, R., Ghazanfari, E. & Izquierdo, E. A. Geomechanical characterization of Marcellus Shale. *Rock Mech. Rock Eng.* **49**, 3403-3424 (2016).

Wang, D., Wang, X., Ge, H., Sun, D. & Yu, B. Experimental study on the failure mechanisms in brittle shales. *ACS Omega* **5**, 10382−10394 (2020).

Wang, S., et al. A universal method for quantitatively evaluating rock brittle-ductile transition behaviors. *J. Petr. Sci. Eng.* **195**, 107774 (2020).

Wang, Y., et al. Anisotropic strength and failure behaviors of transversely isotropic shales: An experimental investigation. *Interpretation* **8**, SL59-SL70 (2020).

Wentworth, C. K. A scale of grade and class terms for clastic sediments. *J. Geol.* **30**, 377-392 (1922).

Whitney, D. L. & Evans, B.W. Abbreviations for names of rock-forming minerals. *Amer. Mineral.* **95**, 185-187 (2010).

Wiseall, A., et al. *Properties and Behaviour of the Boom Clay Formation within a Dutch Repository Concept* (Onderzoeks Programma Eindberging Radioactief Afval, **OPERA-PU-BGS615 Report**, 2015).

Yu, H., et al. Influence of temperature on the hydro-mechanical behavior of Boom Clay. *Int. J. Rock Mech. Min. Sci.* **108**, 189-197 (2018).

Yu, H.-D., et al. Experimental study on the hydro-mechanical behavior of Boom clay. *Int. J. Rock Mech. Min. Sci.* **53**, 159-165 (2012).

Zhang, C.-L., Armand, G., Conil, N. & Laurich, B. Investigation on anisotropy of mechanical properties of Callovo-Oxfordian claystone. *Eng. Geol.* **251**, 128-145 (2019).

Zhang, T., et al. Empirical relationship between gas composition and thermal maturity in Eagle Ford Shale, south Texas. *AAPG Bull.* **101**, 1277-1307 (2017).
